# High Dosage Omega-3 Fatty Acids Outperform Existing Pharmacological Options for Migraine Prophylaxis: A Network Meta-Analysis

**DOI:** 10.1016/j.advnut.2023.100163

**Published:** 2023-12-16

**Authors:** Ping-Tao Tseng, Bing-Yan Zeng, Jiann-Jy Chen, Chun-Hsien Kuo, Bing-Syuan Zeng, John S Kuo, Yu-Shian Cheng, Cheuk-Kwan Sun, Yi-Cheng Wu, Yu-Kang Tu, Brendon Stubbs, Andre F Carvalho, Chih-Sung Liang, Tien-Yu Chen, Chih-Wei Hsu, Mein-Woei Suen, Chun-Pai Yang, Shih-Pin Hsu, Yen-Wen Chen, Yow-Ling Shiue, Chao-Ming Hung, Kuan-Pin Su, Pao-Yen Lin

**Affiliations:** 1 Institute of Biomedical Sciences, National Sun Yat-sen University, Kaohsiung, Taiwan; 2 Department of Psychology, Collage of Medical and Health Science, Taichung, Asia University, Taiwan; 3 Prospect Clinic for Otorhinolaryngology & Neurology, Kaohsiung City, Taiwan; 4 Institute of Precision Medicine, National Sun Yat-sen University, Kaohsiung City, Taiwan; 5 Department of Internal Medicine, E-Da Dachang Hospital, I-Shou University, Kaohsiung, Taiwan; 6 Department of Otorhinolaryngology, E-Da Cancer Hospital, I-Shou University, Kaohsiung, Taiwan; 7 Department of Internal Medicine, E-Da Cancer Hospital, I-Shou University, Kaohsiung, Taiwan; 8 Neuroscience and Brain Disease Center and Graduate Institute of Biomedical Sciences, China Medical University, Taichung, Taiwan; 9 Department of Psychiatry, Tsyr-Huey Mental Hospital, Kaohsiung Jen-Ai’s Home, Taiwan; 10 Department of Emergency Medicine, E-Da Hospital, I-Shou University, Kaohsiung, Taiwan; 11 School of Medicine for International Students, College of Medicine, I-Shou University Kaohsiung, Taiwan; 12 Department of Sports Medicine, Landseed International Hospital, Taoyuan, Taiwan; 13 Institute of Epidemiology & Preventive Medicine, College of Public Health, National Taiwan University, Taipei, Taiwan; 14 Department of Dentistry, National Taiwan University Hospital, Taipei, Taiwan; 15 Department of Psychological Medicine, Institute of Psychiatry, Psychology and Neuroscience, King’s College London, London, United Kingdom; 16 Physiotherapy Department, South London and Maudsley NHS Foundation Trust, London, United Kingdom; 17 Positive Ageing Research Institute (PARI), Faculty of Health, Social Care Medicine and Education, Anglia Ruskin University, Chelmsford, United Kingdom; 18 Innovation in Mental and Physical Health and Clinical Treatment (IMPACT) Strategic Research Centre, School of Medicine, Barwon Health, Deakin University, Geelong, VIC, Australia; 19 Department of Psychiatry, Beitou Branch, Tri-Service General Hospital, Taipei, Taiwan; 20 School of Medicine, National Defense Medical Center, Taipei, Taiwan; 21 Graduate Institute of Medical Sciences, National Defense Medical Center, Taipei, Taiwan; 22 Department of Psychiatry, Tri-Service General Hospital, Taipei, Taiwan; 23 School of Medicine, National Defense Medical Center, Taipei, Taiwan; 24 Institute of Brain Science, National Yang Ming Chiao Tung University, Taipei 112, Taiwan; 25 Department of Psychiatry, Kaohsiung Chang Gung Memorial Hospital and Chang Gung University College of Medicine, Kaohsiung, Taiwan; 26 Gender Equality Education and Research Center, Asia University, Taichung, Taiwan; 27 Department of Medical Research, Asia University Hospital, Asia University, Taichung, Taiwan; 28 Department of Medical Research, China Medical University Hospital, China Medical University, Taichung, Taiwan; 29 Department of Neurology, Kuang Tien General Hospital, Taichung, Taiwan; 30 Department of Nutrition, Hungkuang University, Taichung, Taiwan; 31 Department of Neurology, E-Da hospital, I-Shou University, Kaohsiung, Taiwan; 32 School of Medicine, College of Medicine, I-Shou University, Kaohsiung, Taiwan; 33 Division of General Surgery, Department of Surgery, E-Da Cancer Hospital, I-Shou University, Kaohsiung, Taiwan; 34 Mind-Body Interface Research Center (MBI-Lab), China Medical University Hospital, Taichung, Taiwan; 35 College of Medicine, China Medical University, Taichung, Taiwan; 36 An-Nan Hospital, China Medical University, Tainan, Taiwan; 37 Institute for Translational Research in Biomedical Sciences, Kaohsiung Chang Gung Memorial Hospital

**Keywords:** network meta-analysis, EPA, DHA, polyunsaturated fatty acid, migraine, prevention

## Abstract

Migraine is a highly prevalent neurologic disorder with prevalence rates ranging from 9% to 18% worldwide. Current pharmacologic prophylactic strategies for migraine have limited efficacy and acceptability, with relatively low response rates of 40% to 50% and limited safety profiles. Eicosapentaenoic acid (EPA) and docosahexaenoic acid (DHA) are considered promising therapeutic agents for migraine prophylaxis. The aim of this network meta-analysis (NMA) was to compare the efficacy and acceptability of various dosages of EPA/DHA and other current Food and Drug Administration–approved or guideline-recommended prophylactic pharmacologic interventions for migraine. Randomized controlled trials (RCTs) were eligible for inclusion if they enrolled participants with a diagnosis of either episodic or chronic migraine. All NMA procedures were conducted under the frequentist model. The primary outcomes assessed were *1*) changes in migraine frequency and *2*) acceptability (i.e., dropout for any reason). Secondary outcomes included response rates, changes in migraine severity, changes in the frequency of using rescue medications, and frequency of any adverse events. Forty RCTs were included (*N* = 6616; mean age = 35.0 y; 78.9% women). Our analysis showed that supplementation with high dosage EPA/DHA yields the highest decrease in migraine frequency [standardized mean difference (SMD): −1.36; 95% confidence interval (CI): −2.32, −0.39 compared with placebo] and the largest decrease in migraine severity (SMD: −2.23; 95% CI: −3.17, −1.30 compared with placebo) in all studied interventions. Furthermore, supplementation with high dosage EPA/DHA showed the most favorable acceptability rates (odds ratio: 1.00; 95% CI: 0.06, 17.41 compared with placebo) of all examined prophylactic treatments. This study provides compelling evidence that high dosage EPA/DHA supplementation can be considered a first-choice treatment of migraine prophylaxis because this treatment displayed the highest efficacy and highest acceptability of all studied treatments. This study was registered in PROSPERO as CRD42022319577.

**Statement of Significance**

Based on 40 randomized controlled trials and 6616 participants, high dosage prophylactic EPA/DHA supplementation can be considered a first-choice treatment of migraine prophylaxis because this treatment displayed the highest efficacy and highest acceptability of all studied treatments.

## Introduction

Migraine is a highly prevalent neurologic disorder with a prevalence rate of 9.1% to 18.2% [1,2]. Migraine warrants more attention because it causes significant clinical morbidity, diminishes quality of life, and is associated with potential headache medication overuse around the world. Multiple treatment strategies for migraine prophylaxis, which is defined as migraine frequency reduction [[3], [4], [5]], are currently under investigation [[6], [7], [8]]. However, the response rates for many migraine prophylaxis therapies appear modest (i.e., ∼40%–50%) [9]. Because of the limited efficacy, obvious adverse events, and insufficient evidence for the current pharmacologic treatments to manage prevention, there is an unmet need for more effective and highly acceptable agents to prevent migraine. Although a recent large-scale network meta-analysis (NMA) addressed several new approaches to prevent migraine, such as noninvasive neuromodulation strategies [10], use of monoclonal anticalcitonin gene-related peptide (CGRP) antibodies [11], and supplementation with exogenous melatonin [12], the efficacy and acceptability of the aforementioned treatments still were limited. We aimed to gather and evaluate the evidence for EPA and DHA as a potent preventive migraine therapy that is easily tolerated by patients to improve long-term compliance.

Proposed etiologies of migraine include *1*) the neuroinflammation theory [13] (overtly increased microglia activation, neuroinflammation, and neuropathic pain in the brain [14]); *2*) trigeminal nerve-trigeminocervical complex-ventroposteromedial thalamic nucleus cascade (so-called TVGT pathway, which involves nociceptive transmission and migraine-associated symptoms [15]); and *3*) the vasodilation theory (involving the release of CGRP [16] and other vasoactive peptides [17]). EPA and DHA were found to exert beneficial effects through an anti-inflammatory mechanism [18], reduce nociceptive responses [19], and inhibit the vasodilation in migraine patients [20]. These properties would theoretically benefit migraine management.

Although the hypothesized benefits of EPA/DHA in migraine prophylaxis are highly promising [20], the supporting evidence from randomized controlled trials (RCTs) remains unclear [21,22]. This discrepancy might be due to the following issues. First, different doses of EPA/DHA appear to vary in effectiveness in migraine prophylaxis. Second, some RCTs did not use a “placebo-controlled” design. In clinical trials for headache and pain treatment, a placebo effect was found to be as high as 40% to 55% [[23], [24], [25]]. Third, the age-dependent treatment efficacy (i.e., adult compared with child) is another potential confounding issue [21,26]. Although a prior meta-analysis attempted to resolve this controversy, its overall results were inconclusive [27].

As indicated above, the significant challenge of evaluating potential differences in prophylactic effectiveness of various doses of EPA/DHA cannot be simply resolved by the traditional pairwise meta-analysis or single RCT. Rather, NMA is necessary to improve the power of multiple comparisons of treatment efficacy and possible superiority of individual pharmacologic interventions of different dosages, thereby providing potentially significant detailed evidence-based information to guide future clinical practice. The primary aim of this study was to compare the efficacy and safety profile of different dosages of EPA/DHA with Food and Drug Administration (FDA)-approval or guideline-recommended pharmacologic interventions, based on changes in migraine frequency in patients with migraine.

## Methods

### General guidelines applied in the current study

The present NMA followed the PRISMA 2020 guidelines (Supplemental Table 1) and AMSTAR2 (Assessing the Methodological Quality of Systematic Review) guidelines. The current study was approved by the Institutional Review Board of the Tri-Service General Hospital, National Defense Medical Center, Taipei, Taiwan (TSGHIRB No. B-109–29) and was registered in PROSPERO (CRD42022319577).

### Search strategy and selection criteria

In the NMA, our search strategy consisted of 2 stages. In the first stage, we conducted a systematic review of publications retrieved from PubMed, Embase, ProQuest, ScienceDirect, Cochrane CENTRAL, ClinicalKey, Web of Science, and clinicaltrials.gov from inception to 20 March, 2022, to search for RCTs using ω-3 or ω-6 PUFAs in the management of migraine with/without aura. In the second stage, to include studies about the efficacy/safety of the FDA-approved or guideline-recommended [6] oral forms of medications used for management of migraine with/without aura, we performed an additional search to find RCTs using topiramate, valproate, propranolol, timolol, amitriptyline, venlafaxine, lisinopril, frovatriptan, or candesartan in migraine prevention. We focused on oral medications because of the potential difference between the placebo effect of an oral placebo and that of injected placebo (i.e., the injected form exhibited the highest pain-free rate in migraine management compared with other forms of placebo) [28]. From perspective of statistics, the NMA was based on the hypothesis of similarity. To be specific, according to the similarity hypothesis, the placebo effect of the injected form should be similar to that of the oral form of placebo; otherwise, the similarity hypothesis would not be established, and the NMA would be invalid. Therefore, we did not include the injected forms of treatment in the present NMA in order to fulfill the basic similarity hypothesis of NMA. Specifically, we did not include the botulinum toxin (Bot) or CGRP treatments in this NMA. No language restrictions were applied. The detailed search strategy and keywords applied to each database are depicted in Supplemental Table 2. We also conducted manual searches for potentially eligible articles from the reference lists of review articles or pairwise meta-analyses.

### Inclusion and exclusion criteria

The PICO applied in the present NMA was as follows: *1*) Participants: patients with migraine, either episodic, chronic, or nonspecified; *2*) Intervention: EPA/DHA supplementations or FDA-approval/guideline-recommendation medication to manage migraine; *3*) Comparison: placebo control; and *4*) Outcome: changes in migraine frequency or response rate. We chose the target of migraine frequency reduction based on the definition of migraine prevention in the previous guidelines [2,4,5], which define the migraine prevention to be “migraine frequency reduction.” The response was defined as “≥50% improvement from baseline.” To improve the quality of the included articles and to reduce the unwanted impact of a potential placebo effect (in clinical trials for headache and pain treatment, a placebo effect is found to be as high as 40%–55%) [[23], [24], [25]], we only included peer-reviewed published articles reporting RCTs with either placebo-controlled or active-controlled with applied placebo in the study design. The targets for comparison were pharmacologic interventions used for prophylaxis in patients with migraine but not for acute treatment to migraine attack. Therefore, the inclusion criteria were as follows: *1*) RCTs of migraine patients; *2*) trials investigating pharmacologic interventions for migraine prevention; *3*) human studies; and *4*) placebo-controlled studies.

The exclusion criteria were as follows: *1*) studies that were not clinical trials in humans; *2*) studies that were not RCTs; *3*) studies that recruited patients without migraine; and *4*) studies that did not use a placebo. In cases of duplicated data (i.e., different articles based on the same sample), we only included the reports with more information and larger sample sizes.

### Data extraction

Two authors independently screened the studies, extracted relevant data from the articles, and assessed risk of bias among the included studies. Cases of discrepancy were adjudicated by the corresponding author (YLS, CMH, KPS, and PYL). We divided EPA/DHA supplementations into 3 dosage groups: *1*) EPA+DHA <900 mg/d, *2*) 900–1500 mg/d, and *3*) 1500 mg/d or higher. If the data was not available in the manuscript, we contacted the corresponding author or coauthors to obtain the original data.

### Outcome definition

The primary outcomes were *1*) changes in migraine frequency associated with the pharmacologic interventions and *2*) acceptability (i.e., dropout rate), where dropout was defined as patient withdrawal from the study before its end for any reason. The secondary outcomes were *1*) response rate, which was defined as a 50% reduction in baseline frequency of migraine days after pharmacologic interventions; *2*) changes in migraine severity; *3*) changes in frequency of rescue medication use; and *4*) rate of any adverse events. The selected primary outcomes (frequency of migraine attack and acceptability) and secondary outcomes (response rate and frequency of any adverse events) are widely used in various NMAs of migraine management [10,12].

### Cochrane risk-of-bias tool

Two independent authors evaluated risk of bias (interrater reliability, 0.86) for each domain described in the Cochrane risk-of-bias tool.

### Statistical analysis

The NMA was performed using STATA (version 16.0; StataCorp LLC). For continuous data, we calculated summary standardized mean differences (SMDs) with 95% confidence intervals (CIs). For categorical data, we estimated summary odds ratios (ORs) with 95% CIs. For categorical data, we used a 0.5 zero-cell correction during the meta-analysis procedure. However, if in one study both intervention and control arms were 0, we did not apply this correction procedure because of risk of increasing bias [29]. We used the most frequent NMA model to compare the effect sizes among studies with the same interventions. All comparisons were 2-tailed, and a *P* value ≤ 0.05 denoted statistical significance. Heterogeneity among the included studies was evaluated using the tau value, which is the estimated standard deviation of the effect across the included studies. Regarding the meta-analysis procedure applied in the current study, we used mixed comparisons with generalized linear mixed models to analyze the direct and indirect comparisons for NMA. For comparisons among multiple treatment arms, we combined direct and indirect evidence from the included studies. In the present NMA, we used a suite of STATA programs using “mvmeta” for data manipulation [30]. We used the restricted maximum likelihood method to evaluate between-study variance. To increase the clinical application, we calculated relative ranking probabilities between the preventive effects of all treatments studied for the target outcomes. In brief, the surface under the cumulative ranking curve (SUCRA) is the percentage of the mean rank of each pharmacologic intervention relative to an imaginary intervention that is the best without uncertainty [31]. Finally, we evaluated potential inconsistencies between the direct and indirect evidence within the network using the loop-specific approach and local inconsistencies using the node-splitting method. Further, we used the design-by-treatment model to evaluate global inconsistencies throughout the entire NMA. We used comparison-adjusted funnel plots and Egger regression to evaluate potentially small study effects and publication bias. Finally, we performed subgroup analyses dividing RCTs in subgroups of *1*) adults compared with children; *2*) episodic migraine compared with chronic migraine; or *3*) excluding trials with high risk-of-bias items.

## Results

### Eligibility of the retrieved studies and treatment arms

**FIGURE 1:**
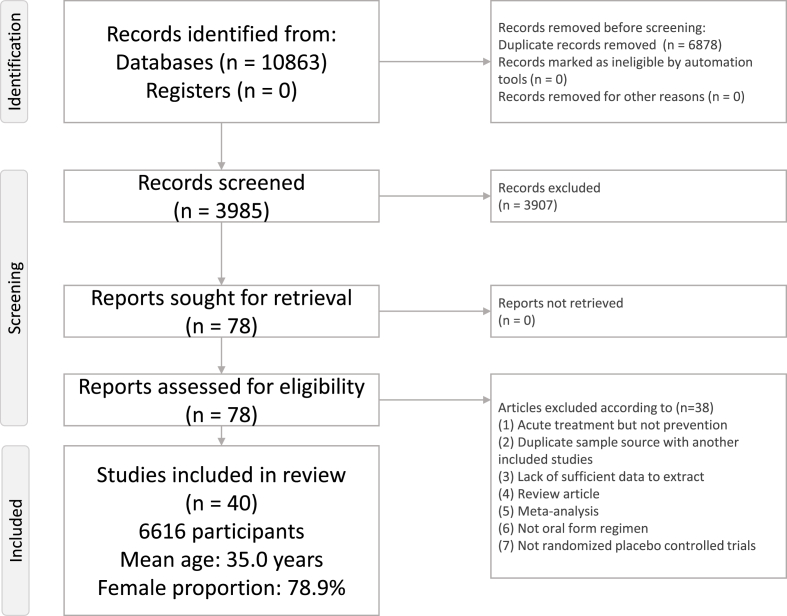
Flowchart of the current network meta-analysis.

**FIGURE 2:**
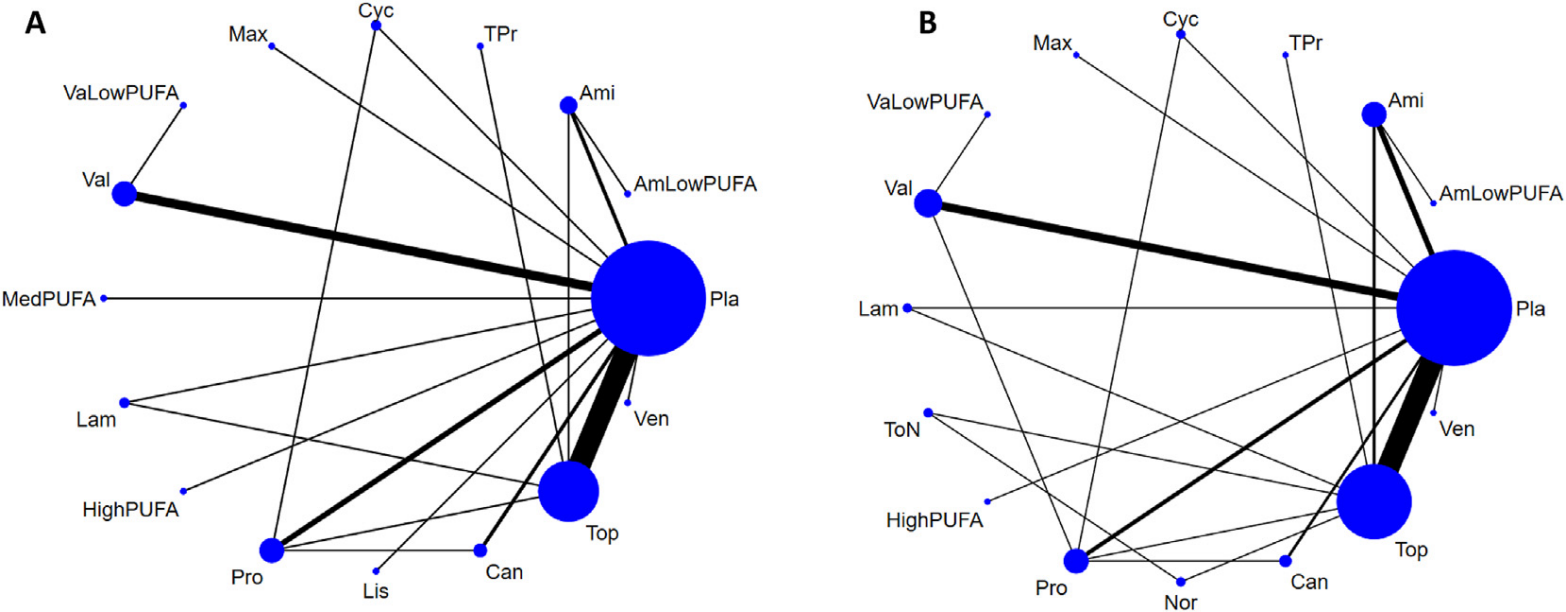
Network structure of the primary outcomes: (A) changes in migraine frequency and (B) acceptability in dropout rate. The lines between nodes represent direct comparisons in various trials, and the size of each circle is proportional to the size of the population involved in each specific treatment. The thickness of the lines is proportional to the number of trials connected to the network. Ami, amitriptyline; AmLowPUFA, low dosage n-3 PUFA + amitriptyline; Can, candesartan; Cyc, cyclandelate; HighPUFA, high dosage n-3 PUFA; Lam, lamotrigine; Lis, lisinopril; Max, Maxepa (ω-3 polyunsaturated fatty acids, eicosapentaenoic acid/docosahexaenoic acid: 180 mg/120 mg × 6 pills); MedPUFA, medium dosage n-3 PUFA; Nor, nortriptyline; Pla, Placebo; Pro, propranolol; PUFA, polyunsaturated fatty acid; ToN, topiramate + nortriptyline; Top, topiramate; TPr, topiramate + propranolol; Val, valproate; VaLowPUFA, low dosage n-3 PUFA + valproate; Ven, venlafaxine

Figure 1 depicts the flowchart of the present NMA. After the initial screening procedure, a total of 78 articles were considered for full-text review, of which 38 were excluded for various reasons (Supplemental Table 3). Finally, 40 RCTs were included in the current study (Table 1) [18,21,22,25,26,[32], [33], [34], [35], [36], [37], [38], [39], [40], [41], [42], [43], [44], [45], [46], [47], [48], [49], [50], [51], [52], [53], [54], [55], [56], [57], [58], [59], [60], [61], [62], [63], [64], [65], [66]]. The overall network structure of the treatment arms is provided in Figure 2A, B.

**TABLE 1:** Characteristics of the included studies

Study name	Disease severity	Diagnosis	Comparison	Number	Mean age, y	Female, %	Treatment duration, wk	Study duration, wk^1^	Result	Country
Trials investigating ω-3 fatty acid supplements										
Abdolahi et al. [18], 2021	≥15 headache d/mo for >3 mo or ≥1 attack/wk	chronic migraine	ω-3 PUFAs (EPA/DHA 600 mg/300 mg) × 2 pills + placebo placebo + placebo	19 19	36.2 ± 1.9 36.5 ± 1.9	20.0 20.0	8	8 + 0	Reduced in Exp group	Iran
Soares et al. [32], 2018	daily headache	chronic migraine	ω-3 PUFAs (EPA/DHA 400 mg /350 mg) + amitriptyline placebo + amitriptyline	27 24	36.9 ± 7.5 34.2 ± 9.9	77.8 62.5	8	8 + 0	Better in Exp group	Brazil
Fayyazi et al. [21], 2016	>1 attack/wk or 3 attacks/mo or 1 d missed school/mo	not specific	ω-3 PUFAs (EPA/DHA 180 mg/120 mg) × 1 pill + valproate placebo + valproate	12 13	10.4 ± 2.9	58.3 53.8	8	8 + 0	No sig. diff.	Iran
Harel et al. [22], 2002	chronic migraine	chronic migraine	ω-3 PUFAs (EPA/DHA 378 mg/249 mg) × 2 pills placebo	14 13	15.0 ± 1.0	70.4	8	8 + 0	No sig. diff.	United States
Pradalier et al. [26], 2001	migraine on 2–6 d/mo	episodic migraine	Maxepa (ω-3 PUFAs, EPA/DHA: 180 mg/120 mg × 6 pills) placebo (olive oil + lactose)	100 96	39.3 ± 11.9 39.2 ± 10.3	82.0 79.0	16	16 + 0	No sig. diff.	France
Trials investigating medication approval for migraine										
Ebrahimi-Monfared et al. [33], 2017	Headache fulfilling criteria for ≥15 d/mo for >3 mo, with ≥1 y history of migraine	chronic migraine	valproate placebo	35 35	38.9 ± 9.2	51.4	8	8 + 0	Reduced in Exp group	Iran
Powers et al. [25], 2017	headache frequency of ≥4 d/mo	not specific	amitriptyline topiramate placebo	144 145 72	14.2 ± 2.4 14.2 ± 2.5 14.2 ± 2.2	67.4 69.7 68.1	24	24 + 6	No sig. diff.	United States
Gonçalves et al. [34], 2016	≥3 migraine headache attacks per month but attacks <15 d/mo	episodic migraine	amitriptyline placebo	59 59	37.2 ± 11.2 36.6 ± 13.7	74.6 76.3	12	12 + 0	No sig. diff.	Brazil
Stovner et al. [35], 2014	≥2 migraine attacks/mo	not specific	candesartan propranolol placebo	72 72 72	37.0 ± 11.0	81.9	12	12 + 0	Better in Exp group	Norway
Krymchantowski et al. [36], 2012	<50% headache frequency improvement at 8 wk relative to baseline by topiramate or nortriptyline	episodic migraine	topiramate + placebo nortriptyline + placebo topiramate + nortriptyline	17 19 44	35.9 ± 7.7 41.2 ± 6.8 36.1 ± 9.5	88.2 84.2 81.8	6	6 + 0	Better in Exp group	Brazil
Silberstein et al. [37], 2012	history of chronic migraine for ≥6 mo	chronic migraine	topiramate + propranolol topiramate + placebo	96 95	39.0 42.0	87.5 92.6	24	24 + 4	No sig. diff.	United States
Couch et al. [38], 2011	≥2 migraine/mo	not specific	amitriptyline placebo	194 197	34.1 35.7	79.4 82.7	16	16 + 0	Better in Exp group	United States
Lipton et al. [39], 2011	≥9 but <15 d/mo	episodic migraine	topiramate placebo	159 171	39.6 ± 10.6 40.9 ± 11.2	86.8 91.2	26	26 + 2	Reduced in Exp group	United States
Dodick et al. [40], 2009	3–12 migraine episodes during the 28-d prospective baseline period, and ≤15 headache days	episodic migraine	topiramate + placebo amitriptyline + placebo	172 159	39.7 ± 10.7 37.9 ± 11.3	86.6 83.0	26	26 + 2	No sig. diff.	United States
Lewis et al. [41], 2009	average of 3–12 migraine episodes on ≤14 headache days	episodic migraine	topiramate placebo	70 33	14.2 ± 1.6 14.4 ± 1.7	60.0 63.6	16	16 + 6	Better in Exp group	Multiple countries
Apostol et al. [42], 2008	≥3 but ≤8 migraine headaches/mo during the 3 mo prior to screening	episodic migraine	valproate placebo	228 71	14.2 ± 1.6 14.2 ± 1.5	55.3 52.1	4	4 + 0	No sig. diff.	United States
Diener et al. [43], 2007	≥15 migraine days/4 wk, at least during the last 3 mo prior to trial entry	chronic migraine	topiramate placebo	32 27	47.8 ± 9.4 44.4 ± 9.6	75.0 74.0	16	16 + 7	Better in Exp group	United States
Gupta et al. [44], 2007	≥4 migraine headache attacks per month and ≤10 attacks/mo	episodic migraine	topiramate lamotrigine placebo	57 57 57	29.4 ± 7.7	78.3	4	4 + 0	Better in topiramate	India
Silberstein et al. [45], 2007	≥15 headache days/28 d	chronic migraine	topiramate placebo	153 153	37.8 ± 12.4 38.6 ± 11.8	83.7 86.9	16	16 + 2	Better in Exp group	United States
Silberstein et al. [46], 2006	average 3–8 migraine episodes/mo (defined as 28 d) for 3 mos (84 d) before screening	episodic migraine	topiramate placebo	138 73	39.9 ± 11.8 41.7 ± 9.4	85.5 86.3	20	20 + 0	No sig. diff.	United States
Winner et al. [47], 2006	3 and 12 migraine attacks and ≤14 headache days/28 d during the 3 mo	episodic migraine	topiramate placebo	37 12	14.0 ± 1.7 15.0 ± 2.0	72.2 75.0	26	26 + 0	Better in Exp group	Multiple countries
Ozyalcin et al. [48], 2005	≥3 and ≤10 attacks/mo and ≤15 headache days/mo	episodic migraine	venlafaxine placebo	41 19	35.8 ± 10.7 38.2 ± 11.2	87.8 94.7	10	10 + 0	Better in Exp group	Turkey
Winner et al. [49], 2005	average 3–10 migraine days/mo for the 3 mo	episodic migraine	topiramate placebo	108 49	11.3 ± 2.5 10.7 ± 2.6	49.1 46.9	20	20 + 0	Better in Exp group	United States
Brandes et al. [50], 2004	average 3–12 migraine episodes/mo (defined as 28 d) for 6 mo	episodic migraine	topiramate placebo	354 114	39.1 ± 12.5 38.3 ± 12.0	88.1 82.5	26	26 + 0	Better in Exp group	United States
Diener et al. [51], 2004	Subjects with 3–12 migraine headaches (periods) and ≤15 headache days (including migraine days)	episodic migraine	topiramate propranolol placebo	282 143 143	41.2 ± 11.2 40.6 ± 11.1 40.4 ± 10.1	79.8 83.2 76.2	26	26 + 0	Better in Exp group	Multiple countries
Mei et al. [52], 2004	frequency of the crises ranging from 2–6/mo	episodic migraine	topiramate placebo	35 37	39.7 ± 12.0 38.7 ± 11.0	54.3 54.1	16	16 + 0	Better in Exp group	Italy
Silberstein et al. [53], 2004	3–12 migraines during the prospective 28-d baseline phase	episodic migraine	topiramate placebo	354 115	40.4 ± 11.3 40.4 ± 11.5	88.4 89.6	26	26 + 0	Better in Exp group	United States
Edwards et al. [54], 2003	migraine ≥1 y with ≥2 attacks/mo	episodic migraine	topiramate placebo	34 36	41.1	97.1	20	20 + 0	Better in Exp group	United States
Silvestrini et al. [55], 2003	history of migraine without aura attacks for ≥10 y	chronic migraine	topiramate placebo	14 14	43.0 44.0	64.3 64.3	9	9 + 0	Better in Exp group	Italy
Tronvik et al. [56], 2003	2–6 attacks/mo	episodic migraine	candesartan placebo	28 29	NA	NA	12	12 + 0	Better in Exp group	Norway
Freitag et al. [57], 2002	migraine headache ≥6 mo before screening and an average of ≥2 migraine headaches/mo during the 3 mo	episodic migraine	valproate placebo	122 115	39.8 ± 11.2 41.3 ± 12.0	79.5 78.3	12	12 + 1	Better in Exp group	United States
Schrader et al. [58], 2001	more than a year, migraine occurring 2–6 times/mo	episodic migraine	lisinopril placebo	30 30	41.4 ± 8.4	80.9	12	12 + 0	Better in Exp group	Norway
Storey et al. [59], 2001	experienced migraine attacks for >1 y at a frequency of ≥2 attacks/mo	episodic migraine	topiramate placebo	19 21	38.3 38.1	100.0 95.2	16	16 + 0	Better in Exp group	United States
Kaniecki [60], 1997	migraine frequency 2–8 times/mo, with a maximum of 15 headache days/mo for >1 y	episodic migraine	valproate propranolol	32 32	NA	81.1	21	21 + 0	Both improved in Exp group	United States
Klapper [61], 1997	migraine for ≥6 mo, average ≥2 migraine/mo	episodic migraine	valproate placebo	132 44	41.0 40.2	88.4 91.0	12	12 + 0	Better in Exp group	United States
Diener et al. [62], 1996	history of migraine for ≥12 mo, 2–10 migraine attacks/mo	episodic migraine	cyclandelate propranolol placebo	67 68 39	39.0 ± 12.0 40.0 ± 13.0 39.0 ± 11.0	81.5 76.9 74.5	12	12 + 2	No sig. diff.	Switzerland
Mathew et al. [63], 1995	≥2 episodes/month for the previous 3 mo	episodic migraine	valproate placebo	58 32	47.0 43.0	80.0 73.0	12	12 + 0	Better in Exp group	United States
Jensen et al. [64], 1994	history of migraine for ≥1 y, 2–10 d with migraine/mo	episodic migraine	valproate placebo	22 21	45.0 47.0	81.8 90.5	12	12 + 0	Better in Exp group	Denmark
Ziegler et al. [65], 1987	more than half of the headaches were severe or disabling, not less than an average of twice a month nor more often than 3 times/wk	episodic migraine	amitriptyline propranolol placebo	30 30	38	73.3	4	4 + 0	Better in Exp group	United States
Couch and Hassanein [66], 1979	≥2 disabling or severe migraine headaches in the month	not specific	amitriptyline placebo	47 53	NA	83.0 84.9	4	4 + 0	Better in Exp group	United States

Abbreviations: Ctr group, control group; DHA, docosahexaenoic acid; EPA, eicosapentaenoic acid; Exp group, experimental group; NA, not available; PUFA, polyunsaturated fatty acid; sig. diff., significant difference.

1 Study duration: treatment duration + posttreatment follow-up duration.

### Characteristics of the included studies

A total of 6616 participants (mean age 35.0 y, range 10.36–46.2 y; mean female proportion 78.9%, range 20.0–97.5) were included. The mean treatment duration was 18.0 wk (from 4.0 to 26.0 wk). The mean overall study duration (i.e., treatment + posttreatment follow-up) was 19.0 wk (from 4.0 to 30.0 wk).

### Primary outcome: *1*) changes in frequency of migraine days

**FIGURE 3:**
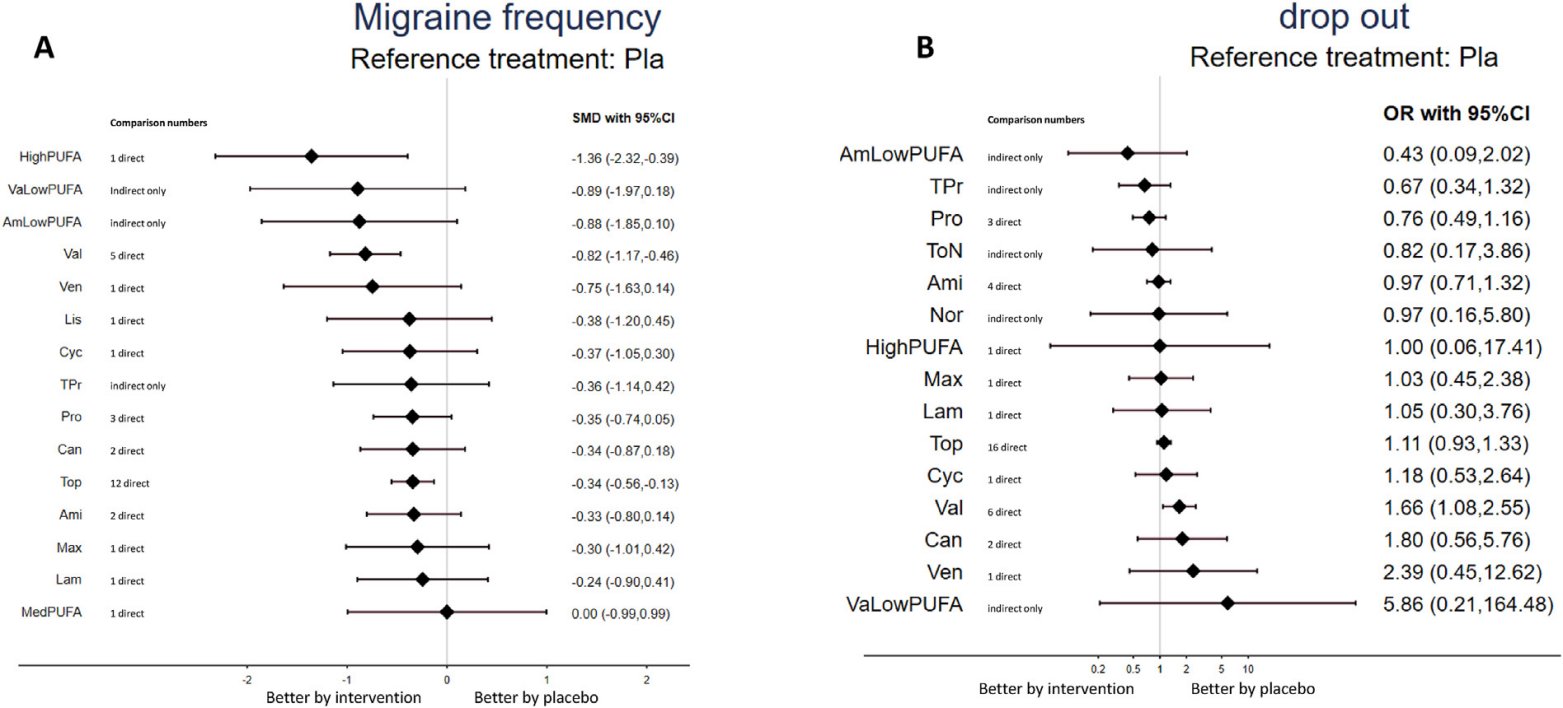
Forest plot of the primary outcomes with reference to placebo: (A) changes in migraine frequency and (B) acceptability in dropout rate. Specific treatments were associated with (A) better improvement in migraine frequency than the placebo if the standardized mean difference was <0 or (B) better acceptability in dropout rate than the placebo if the odds ratio was < 1. Ami, amitriptyline; AmLowPUFA, low dosage n-3 PUFA + amitriptyline; Can, candesartan; Cyc, cyclandelate; HighPUFA, high dosage n-3 PUFA; Lam, lamotrigine; Lis, lisinopril; Max, Maxepa (ω-3 polyunsaturated fatty acids, eicosapentaenoic acid/docosahexaenoic acid: 180 mg/120 mg × 6 pills); MedPUFA, medium dosage n-3 PUFA; Nor, nortriptyline; Pla, Placebo; Pro, propranolol; PUFA, polyunsaturated fatty acid; ToN, topiramate + nortriptyline; Top, topiramate; TPr, topiramate + propranolol; Val, valproate; VaLowPUFA, low dosage n-3 PUFA + valproate; Ven, venlafaxine.

The main result of this NMA revealed that high dosage EPA/DHA supplementation (HighPUFA) (SMD: −1.36; 95% CI: −2.32, −0.39), valproate (SMD: −0.82; 95% CI: −1.17, −0.46), and topiramate (SMD: −0.34; 95% CI: −0.56, −0.13) were associated with significantly better improvements in frequency of migraine days than placebo (Table 2 and Figure 3A). According to the SUCRA, HighPUFA was associated with the greatest improvement in frequency of migraine days among all pharmacologic interventions, followed by valproate and topiramate (Supplemental Table 5A).

**TABLE 2:** League table of changes in frequency of migraine attacks

HighPUFA	—	—	—	—	—	—	—	—	—	—	—	—	—	—	−1.36 (−2.07, −0.64)^1^
−0.54 (−1.56, 0.49)	Val	—	0.08 (−0.71, 0.86)	—	—	—	—	—	—	—	—	—	—	—	−0.86 (−1.49, −0.23)^1^
−0.48 (−1.85, 0.89)	0.06 (−0.98, 1.10)	AmLowPUFA	—	—	—	—	—	—	—	—	−0.54 (−1.11, 0.02)	—	—	—	—
−0.46 (−1.91, 0.98)	0.08 (−0.94, 1.09)	0.02 (−1.44, 1.47)	VaLowPUFA	—	—	—	—	—	—	—	—	—	—	—	—
−0.61 (−1.92, 0.70)	−0.07 (−1.03, 0.88)	−0.13 (−1.45, 1.19)	−0.15 (−1.54, 1.25)	Ven	—	—	—	—	—	—	—	—	—	—	−0.75 (−1.35, −0.14)^1^
−0.98 (−2.25, 0.29)	−0.44 (−1.34, 0.46)	−0.50 (−1.78, 0.78)	−0.52 (−1.87, 0.84)	−0.37 (−1.58, 0.84)	Lis	—	—	—	—	—	—	—	—	—	−0.38 (−0.89, 0.14)
−0.98 (−2.16, 0.19)	−0.45 (−1.21, 0.32)	−0.50 (−1.69, 0.68)	−0.52 (−1.79, 0.75)	−0.37 (−1.49, 0.74)	−0.00 (−1.07, 1.06)	Cyc	—	—	−0.03 (−0.37, 0.30)	—	—	—	—	—	−0.36 (−0.76, 0.04)
−1.01 (−2.11, 0.08)	−0.47 (−1.11, 0.16)	−0.53 (−1.64, 0.58)	−0.55 (−1.75, 0.65)	−0.40 (−1.43, 0.63)	−0.03 (−1.01, 0.94)	−0.03 (−0.85, 0.79)	Can	—	0.02 (−0.35, 0.38)	—	—	—	—	—	−0.32 (−0.62, −0.02)^1^
−1.00 (−2.24, 0.24)	−0.46 (−1.31, 0.39)	−0.52 (−1.76, 0.72)	−0.54 (−1.86, 0.79)	−0.39 (−1.57, 0.79)	−0.02 (−1.15, 1.12)	−0.01 (−1.04, 1.01)	0.01 (−0.92, 0.95)	TPr	—	−0.02 (−0.39, 0.36)	—	—	—	—	—
−1.01 (−2.05, 0.03)	−0.47 (−1.00, 0.06)	−0.53 (−1.58, 0.52)	−0.55 (−1.69, 0.60)	−0.40 (−1.37, 0.57)	−0.03 (−0.94, 0.88)	−0.03 (−0.69, 0.64)	0.00 (−0.57, 0.58)	−0.01 (−0.87, 0.85)	Pro	−0.10 (−0.30, 0.10)	—	—	—	—	−0.31 (−0.48, −0.13)^1^
−1.01 (−2.00, −0.03)^1^	−0.47 (−0.89, −0.06)^1^	−0.53 (−1.52, 0.45)	−0.55 (−1.65, 0.55)	−0.40 (−1.32, 0.51)	−0.03 (−0.88, 0.82)	−0.03 (−0.73, 0.67)	−0.00 (−0.56, 0.56)	−0.02 (−0.76, 0.73)	−0.00 (−0.43, 0.42)	Top	0.00 (−0.23, 0.23)	—	−0.30 (−0.67, 0.07)	—	−0.31 (−0.46, −0.17)^1^
−1.02 (−2.10, 0.05)	−0.49 (−1.07, 0.10)	−0.54 (−1.40, 0.31)	−0.56 (−1.74, 0.61)	−0.41 (−1.42, 0.59)	−0.04 (−0.99, 0.91)	−0.04 (−0.86, 0.78)	−0.01 (−0.72, 0.69)	−0.03 (−0.92, 0.87)	−0.01 (−0.62, 0.59)	−0.01 (−0.50, 0.48)	Ami	—	—	—	−0.28 (−0.63, 0.07)
−1.06 (−2.26, 0.14)	−0.52 (−1.32, 0.28)	−0.58 (−1.79, 0.63)	−0.60 (−1.89, 0.69)	−0.45 (−1.59, 0.69)	−0.08 (−1.17, 1.01)	−0.08 (−1.06, 0.91)	−0.05 (−0.93, 0.84)	−0.06 (−1.12, 0.99)	−0.05 (−0.87, 0.77)	−0.05 (−0.79, 0.70)	−0.04 (−0.89, 0.82)	Max	—	—	−0.30 (−0.60, 0.01)
−1.11 (−2.27, 0.05)	−0.57 (−1.32, 0.17)	−0.63 (−1.80, 0.54)	−0.65 (−1.91, 0.61)	−0.50 (−1.60, 0.60)	−0.13 (−1.18, 0.92)	−0.13 (−1.06, 0.81)	−0.10 (−0.93, 0.74)	−0.11 (−1.11, 0.88)	−0.10 (−0.86, 0.65)	−0.10 (−0.75, 0.55)	−0.09 (−0.88, 0.71)	−0.05 (−1.02, 0.92)	Lam	—	−0.45 (−0.82, −0.08)^1^
−1.36 (−2.74, 0.03)	−0.82 (−1.87, 0.24)	−0.88 (−2.27, 0.52)	−0.89 (−2.36, 0.57)	−0.75 (−2.08, 0.59)	−0.38 (−1.67, 0.92)	−0.37 (−1.57, 0.83)	−0.34 (−1.47, 0.78)	−0.36 (−1.62, 0.90)	−0.35 (−1.41, 0.72)	−0.34 (−1.36, 0.67)	−0.33 (−1.43, 0.77)	−0.30 (−1.52, 0.93)	−0.24 (−1.43, 0.95)	MedPUFA	0.00 (−0.76, 0.76)
−1.36 (−2.32, −0.39)^1^	−0.82 (−1.17, −0.46)^1^	−0.88 (−1.85, 0.10)	−0.89 (−1.97, 0.18)	−0.75 (−1.63, 0.14)	−0.38 (−1.20, 0.45)	−0.37 (−1.05, 0.30)	−0.34 (−0.87, 0.18)	−0.36 (−1.14, 0.42)	−0.35 (−0.74, 0.05)	∗−0.34 (−0.56, −0.13)	−0.33 (−0.80, 0.14)	−0.30 (−1.01, 0.42)	−0.24 (−0.90, 0.41)	0.00 (−0.99, 0.99)	Pla

Pairwise (upper-right portion) and network (lower-left portion) meta-analysis results are presented as estimate effect sizes for the outcome of improvement in frequency of migraine attack. Interventions are reported in order of mean ranking of treatment effect, and outcomes are expressed as SMD (95% confidence interval). For the pairwise meta-analyses, SMD <0 indicates the treatment specified in the row showed better improvement in migraine attack frequency than that specified in the column. For the network meta-analysis, SMD < 0 indicates the treatment specified in the column showed better improvement in migraine attack frequency than that specified in the row.

Abbreviation: Ami, amitriptyline; AmLowPUFA, low-dose n-3 PUFA + amitriptyline; Can, candesartan; Cyc, cyclandelate; HighPUFA, high dosage n-3 PUFA; Lam, lamotrigine; Lis, lisinopril; Max: Maxepa (ω-3 polyunsaturated fatty acids, eicosapentaenoic acid/docosahexaenoic acid: 180 mg/120 mg × 6 pills); MedPUFA, medium dosage n-3 PUFA; Pla, placebo; Pro, propranolol; PUFA, polyunsaturated fatty acid; SMD, standardized mean difference; Top, topiramate; TPr, topiramate + propranolol; Val, valproate; VaLowPUFA, low-dose n-3 PUFA + valproate; Ven, venlafaxine.

1 Indicates statistically significant results.

#### Subgroup of adults compared with children.

When focusing on RCTs with adult participants, HighPUFA (SMD: −1.36; 95% CI: −2.07, −0.64), valproate (SMD: −1.09; 95% CI: −1.32, −0.85), low dosage EPA/DHA + amitriptyline (AmLowPUFA) (SMD: −1.02; 95% CI: −1.69, −0.35), venlafaxine (SMD: −0.75; 95% CI: −1.35, −0.14), amitriptyline (SMD: −0.48; 95% CI: −0.84, −0.11), cyclandelate (SMD: −0.39; 95% CI: −0.71, −0.06), propranolol (SMD: −0.37; 95% CI: −0.52, −0.21), candesartan (SMD: −0.34; 95% CI: −0.62, −0.06), and topiramate (SMD: −0.34; 95% CI: −0.43, −0.25) were associated with significantly better improvements in frequency of migraine days than placebo (Supplemental Table 4A, Supplemental Figure 1A, and Supplemental Figure 2A). According to the SUCRA, HighPUFA was associated with the greatest reduction in frequency of migraine days of all the pharmacologic interventions, followed by valproate and AmLowPUFA (Supplemental Table 5B).

When focusing on RCTs that enrolled children, none of the investigated treatments were associated with significant differences in the frequency of migraine days compared with the placebo (Supplemental Table 4B, Supplemental Figure 1B, Supplemental Figure 2B, and Supplemental Table 5C).

#### Subgroup of episodic compared with chronic migraine.

When focusing on RCTs of participants with episodic migraine, only valproate (SMD: −0.76; 95% CI: −1.18, −0.33) was associated with significantly better improvements in the frequency of episodic migraine days than placebo (Supplemental Table 4C, Supplemental Figure 1C, and Supplemental Figure 2C). According to the SUCRA, valproate was associated with the greatest improvement in the frequency of episodic migraine days of all the pharmacologic interventions (Supplemental Table 5D).

When focusing on RCTs with chronic migraine participants, none of the investigated treatments were associated with significantly different changes in the frequency of chronic migraine days compared with the placebo (Supplemental Table 4D, Supplemental Figure 1D, Supplemental Figure 2D, and Supplemental Table 5E).

#### Subgroup excluding RCTs with high risk-of-bias items.

When focusing on RCTs without high risk-of-bias items, the main results remained unchanged that HighPUFA (SMD: −1.36; 95% CI: −2.18, −0.53), low dosage EPA/DHA + valproate (SMD: −1.20; 95% CI: −2.31, −0.10), valproate (SMD: −1.13; 95% CI: −1.78, −0.47), AmLowPUFA (SMD: −0.91; 95% CI: −1.69, −0.13), venlafaxine (SMD: −0.75; 95% CI: −1.48, −0.01), topiramate (SMD: −0.45; 95% CI: −0.71, −0.18), and amitriptyline (SMD: −0.36; 95% CI: −0.71, −0.02) were associated with significantly better improvements in frequency of migraine days than placebo (Supplemental Figure 1E and Supplemental Figure 2E).

### Primary outcome: *2*) acceptability of dropout rates

We also evaluated the acceptability of the investigated pharmacologic interventions using the NMA. In brief, only valproate (OR: 1.66; 95% CI: 1.08, 2.55) was associated with significantly higher dropout rates than the placebo (Table 3 and Figure 3B). According to the SUCRA, AmLowPUFA (OR: 0.43; 95% CI: 0.09, 2.02 compared with placebo) was associated with the lowest dropout rate (Supplemental Table 5F).

**TABLE 3:** League table of dropout rates

AmLowPUFA	—	—	—	0.44 (0.10, 1.97)	—	—	—	—	—	—	—	—	—	—	—
0.64 (0.12, 3.43)	TPr	—	—	—	—	—	—	—	—	0.60 (0.34, 1.09)	—	—	—	—	—
0.57 (0.11, 2.81)	0.89 (0.41, 1.93)	Pro	—	—	0.98 (0.63, 1.51)	—	—	—	—	**0.52 (0.34, 0.81)**^**1**^	0.74 (0.32, 1.68)	0.58 (0.13, 2.53)	—	0.23 (0.02, 2.16)	—
0.53 (0.06, 4.66)	0.82 (0.15, 4.36)	0.93 (0.19, 4.58)	ToN	—	—	0.84 (0.19, 3.79)	—	—	—	0.74 (0.16, 3.36)	—	—	—	—	—
0.44 (0.10, 2.02)	0.69 (0.34, 1.42)	0.78 (0.47, 1.30)	0.85 (0.18, 4.07)	Ami	0.87 (0.62, 1.21)	—	—	—	—	0.98 (0.67, 1.43)	—	—	—	—	—
0.43 (0.09, 2.02)	0.67 (0.34, 1.32)	0.76 (0.49, 1.16)	0.82 (0.17, 3.86)	0.97 (0.71, 1.32)	Pla	—	0.97 (0.44, 2.13)	1.00 (0.24, 4.21)	1.00 (0.06, 17.18)	0.89 (0.73, 1.08)	0.69 (0.27, 1.75)	0.41 (0.10, 1.64)	0.42 (0.08, 2.16)	**0.62 (0.41, 0.94)**^**1**^	—
0.44 (0.04, 4.66)	0.69 (0.10, 4.57)	0.78 (0.13, 4.84)	0.84 (0.18, 3.88)	1.00 (0.16, 6.04)	1.03 (0.17, 6.11)	Nor	—	—	—	0.87 (0.15, 5.05)	—	—	—	—	—
0.42 (0.07, 2.42)	0.65 (0.22, 1.90)	0.73 (0.29, 1.88)	0.79 (0.14, 4.62)	0.94 (0.39, 2.28)	0.97 (0.42, 2.23)	0.94 (0.13, 6.76)	Max	—	—	—	—	—	—	—	—
0.41 (0.06, 3.01)	0.64 (0.15, 2.65)	0.72 (0.19, 2.73)	0.78 (0.11, 5.72)	0.92 (0.25, 3.38)	0.95 (0.27, 3.38)	0.92 (0.10, 8.19)	0.98 (0.21, 4.48)	Lam	—	1.00 (0.24, 4.21)	—	—	—	—	—
0.43 (0.02, 11.10)	0.67 (0.04, 12.66)	0.76 (0.04, 13.63)	0.82 (0.03, 21.16)	0.97 (0.05, 17.16)	1.00 (0.06, 17.41)	0.97 (0.03, 28.27)	1.03 (0.05, 20.28)	1.05 (0.05, 24.05)	HighPUFA	—	—	—	—	—	—
0.39 (0.08, 1.82)	0.60 (0.32, 1.15)	0.68 (0.44, 1.05)	0.74 (0.16, 3.44)	0.87 (0.64, 1.19)	0.90 (0.75, 1.08)	0.88 (0.15, 5.16)	0.93 (0.40, 2.18)	0.95 (0.27, 3.38)	0.90 (0.05, 15.73)	Top	—	—	—	—	—
0.36 (0.06, 2.08)	0.57 (0.20, 1.61)	0.64 (0.29, 1.40)	0.69 (0.12, 3.96)	0.82 (0.35, 1.93)	0.85 (0.38, 1.89)	0.82 (0.12, 5.81)	0.87 (0.27, 2.79)	0.89 (0.20, 4.00)	0.85 (0.04, 16.47)	0.94 (0.42, 2.12)	Cyc	—	—	—	—
0.24 (0.03, 1.66)	0.37 (0.10, 1.43)	0.42 (0.13, 1.36)	0.46 (0.07, 3.16)	0.54 (0.16, 1.80)	0.56 (0.17, 1.79)	0.54 (0.06, 4.55)	0.58 (0.14, 2.41)	0.59 (0.10, 3.29)	0.56 (0.03, 12.18)	0.62 (0.19, 2.00)	0.66 (0.17, 2.61)	Can	—	—	—
0.18 (0.02, 1.75)	0.28 (0.05, 1.69)	0.32 (0.06, 1.77)	0.34 (0.04, 3.33)	0.41 (0.07, 2.20)	0.42 (0.08, 2.21)	0.41 (0.04, 4.67)	0.43 (0.07, 2.78)	0.44 (0.05, 3.58)	0.42 (0.02, 11.41)	0.47 (0.09, 2.48)	0.49 (0.08, 3.14)	0.75 (0.10, 5.73)	Ven	—	—
0.26 (0.05, 1.29)	**0.40 (0.18, 0.90)**^**1**^	**0.46 (0.25, 0.82)**^**1**^	0.49 (0.10, 2.46)	**0.58 (0.34, 0.99)**^**1**^	**0.60 (0.39, 0.92)**^**1**^	0.59 (0.09, 3.67)	0.62 (0.24, 1.59)	0.63 (0.17, 2.42)	0.60 (0.03, 10.80)	0.67 (0.42, 1.06)	0.71 (0.29, 1.76)	1.08 (0.31, 3.72)	1.44 (0.26, 8.01)	Val	0.28 (0.01, 7.67)
0.07 (0.00, 2.90)	0.11 (0.00, 3.45)	0.13 (0.00, 3.73)	0.14 (0.00, 5.53)	0.17 (0.01, 4.71)	0.17 (0.01, 4.79)	0.17 (0.00, 7.30)	0.18 (0.01, 5.49)	0.18 (0.01, 6.39)	0.17 (0.00, 13.78)	0.19 (0.01, 5.36)	0.20 (0.01, 6.22)	0.31 (0.01, 10.48)	0.41 (0.01, 16.96)	0.28 (0.01, 7.75)	VaLowPUFA

Pairwise (upper-right portion) and network (lower-left portion) meta-analysis results are presented as estimate effect sizes for the outcome of dropout rate. Interventions are reported in order of mean ranking of tolerability, and outcomes are expressed as OR (95% confidence interval). For the pairwise meta-analyses, OR < 1 indicates the treatment specified in the row had a lower dropout rate than that specified in the column. For the network meta-analysis, OR < 1 indicates the treatment specified in the column had a lower dropout rate than that specified in the row. ^1 1^Indicates statistically significant results.

Abbreviations: Ami, amitriptyline; AmLowPUFA, low dosage n-3 PUFA + amitriptyline; Can, candesartan; Cyc, cyclandelate; HighPUFA, high dosage n-3 PUFA; Lam, lamotrigine; Max, Maxepa (ω-3 PUFAs, eicosapentaenoic acid/docosahexaenoic acid: 180 mg/120 mg × 6 pills); Nor, nortriptyline; OR, odds ratio; Pla, placebo; Pro, propranolol; PUFA, polyunsaturated fatty acid; ToN, topiramate + nortriptyline; Top, topiramate; TPr, topiramate + propranolol; Val, valproate; VaLowPUFA, low-dose n-3 PUFA + valproate; Ven, venlafaxine.

### Secondary outcome: response rate

The NMA results showed that AmLowPUFA (OR: 51.72; 95% CI: 8.10, 330.29), topiramate + nortriptyline (ToN) (OR: 9.26; 95% CI: 2.01, 42.56), candesartan (OR: 4.27; 95% CI: 1.71, 10.66), topiramate (OR: 2.42; 95% CI: 1.72, 3.40), propranolol (OR: 2.41; 95% CI: 1.43, 4.07), valproate (OR: 2.18; 95% CI: 1.34, 3.52), and amitriptyline (OR: 1.68; 95% CI: 1.04, 2.70) were associated with significantly higher response rates than placebo (Supplemental Table 4E, Supplemental Figure 1F, and Supplemental Figure 2F). According to the SUCRA, AmLowPUFA was associated with the highest response rate of all the pharmacologic interventions, followed by ToN and candesartan (Supplemental Table 5G).

### Secondary outcome: changes in severity of migraine attack

The NMA results revealed that HighPUFA (SMD: −2.23; 95% CI: −3.17, −1.30), medium dosage n-3 PUFA (MedPUFA) (SMD: −1.28; 95% CI: −2.23, −0.33), valproate (SMD: −1.00; 95% CI: −1.67, −0.34), and topiramate (SMD: −0.32; 95% CI: −0.58, −0.07) were associated with significantly better improvements in severity of migraine attack than placebo (Supplemental Table 4F, Supplemental Figure 1G, and Supplemental Figure 2G). According to the SUCRA, HighPUFA was associated with the greatest improvement in the frequency of migraine days of all the pharmacologic interventions, followed by MedPUFA and valproate (Supplemental Table 5H).

### Secondary outcome: frequency of any adverse event

Venlafaxine (OR: 36.00; 95% CI: 3.27, 396.39), ToN (OR: 8.18; 95% CI: 1.68, 39.80), amitriptyline (OR: 4.35; 95% CI: 2.16, 8.76), topiramate + propranolol (OR: 4.25; 95% CI: 1.24, 14.54), and topiramate (OR: 2.96; 95% CI: 1.95, 4.50) were associated with a significantly higher frequency of any adverse event during the pharmacologic intervention than placebo (Supplemental Table 4G, Supplemental Figure 1H, and Supplemental Figure 2H). According to the SUCRA, the placebo was associated with the lowest frequency of any adverse event of all the investigated treatment arms (Supplemental Table 5I).

### Risk of bias and publication bias

We found that 63.6% (178/280 items), 27.5% (77/280 items), and 8.9% (25/280 items) of the included studies had an overall low, unclear, and high risk of bias, respectively. The funding sources and concealing procedure after randomization mainly contributed to the high and unclear risk of bias, respectively (Supplemental Figure 3A, B).

Funnel plots of publication bias and Egger test across the included studies (Supplemental Figure 4A–J) revealed general symmetry and no significance among the recruited studies in this NMA. The inconsistency test revealed nonsignificant inconsistency in the present NMA (Supplemental Tables 6–8). The results of GRADE evaluation are listed in Supplemental Table 9. In brief, the overall quality of evidence ranged from low to medium.

## Discussion

To the best of our knowledge, the present NMA is the first to investigate associations between the effects of migraine prophylaxis with EPA/DHA supplements compared with other FDA-approved/guideline-recommended medications. The results of this study demonstrated that EPA/DHA supplements conferred noninferior prophylactic effects compared with other FDA-approved/guideline-recommended medications in migraine patients in both efficacy and acceptability. To be specific, migraine prophylaxis with HighPUFA was associated with the greatest improvements in migraine frequency and migraine severity of all the investigated treatments. However, EPA/DHA supplements had similar acceptability compared with other pharmacologic treatments and placebo. The combination of FDA-approval/guideline-recommended medications and EPA/DHA supplements (i.e., amitriptyline plus EPA/DHA) was also associated with the highest response rate of all of the studied treatments. Finally, treatment with EPA/DHA supplementation was associated with a favorable low frequency of adverse events compared with placebo and other pharmacologic interventions.

The most important result of the present NMA is that EPA/DHA supplementation was associated with a superior prophylactic effect on migraine frequency/severity compared with other FDA-approved/guideline-recommended medications. As mentioned previously, the most widely accepted etiologies of migraine are *1*) the neuroinflammation theory; *2*) TVGT pathway; and *3*) the vasodilation theory. The effects of prescription of EPA/DHA supplements contradicts these etiologies. Specifically, EPA/DHA reduced the expression of tumor necrosis factor α (TNF-α) [67], cyclooxygenase-2/NO synthase induction [68], and IL-1β concentrations [69], which are believed to contribute to neuroinflammation and neurogenic pain in the central nervous system. Furthermore, the addition of EPA/DHA was found to have a beneficial effect on reducing nociceptive responses in patients with neuropathic pain, which might occur through activation of the opioid system [19,70,71]. In addition, EPA/DHA supplements helped restore serotonin and dopamine concentrations, which play an important role in the TVGT nociceptive pathway [72]. Finally, dietary EPA/DHA can also inhibit TNF-α expression [67], which may lead to reduced cerebral vasodilation.

In summary, the above evidence supports the hypothesis that EPA/DHA supplementation ameliorates dysregulation of many pathways underlying migraine-related pathophysiology and confers potent migraine prophylaxis. In addition to high efficacy, patient acceptance and compliance with EPA/DHA supplementation is revealed by this NMA and a prior large-scale meta-analysis [73]. All these studies also show the clinically significant finding of higher rates of adverse events with traditional pharmacologic regimens for migraine prophylaxis such as valproate, which limits clinical options for managing migraine [74]. Therefore, the evidence from this NMA provides a sound rationale for future large-scale RCTs to investigate the potential role and optimal dosing of EPA/DHA supplementation in migraine prophylaxis. However, the interpretation of our NMA findings require caution before gathering any supporting or opposing data from head-to-head RCTs. To the contrary, although we recognize that there are currently several RCTs of dietary ω-3 fatty acid supplements addressing migraine prevention [20,75], we did not include them in our NMA because they did not include adequate placebo control in their study design [20] or recruited patients with chronic headache but not migraine [75]. As addressed in methods section, a potential biased placebo effect (in clinical trials for headache and pain treatment, a placebo effect is found to be high as high as 40%–55%) [[23], [24], [25]] might impose bias in the present NMA, so we excluded “studies that did not use placebo” in our study design. Also, the design of control groups in dietary ω-3 fatty acid RCTs often uses concurrent prophylactic pharmacy, which would result in a difference between the control groups in other RCTs that might violate the similarity hypothesis of NMA. In addition, the inclusion of patients with a different diagnosis might also violate the basic similarity hypothesis of NMA.

### Strengths and limitations

This pilot NMA has several strengths. First, due to the large numbers of included RCTs and participants (40 RCTs and 6616 participants), the present NMA could provide more information and higher evidence than RCTs and traditional meta-analyses. Second, we only included RCTs and trials using placebo to increase the reliability of the present study. As addressed before, in pain and headache research, the placebo effect has been reported to account for 40% to 55% of the treatment effect [25]. Third, to be more clinically applicable, we performed additional searches to include RCTs of FDA-approved/guideline-recommended regimens in the present study, which could help clinicians to make relevant comparisons with traditional pharmacologic interventions.

There are also several limitations to the present NMA. First, some analyses in this study were limited by underpowered statistics, including heterogeneity in the characteristics of the participants (e.g., underlying diseases, different concomitant medications, wide variety of ages, lack of uniform diagnostic criteria for migraine, and trial duration) and the small number of trials in some treatment arms. Second, although there was more and more evidence addressing the efficacy of different ratios of EPA/DHA, we could not further classify studies due to the limited information regarding EPA/DHA ratio. Third, in order to fulfill the basic similarity hypothesis of NMA, we did not include the injected forms of treatment, such as Bot and CGRP treatment, in the present NMA. Although statistically, this is a necessary strategy, this exclusion might limit the clinical application of this NMA. Fourth, some of the included RCTs have potential quality concerns in their methodology. Therefore, readers should use caution when interpreting the results of the present NMA. Finally, although our study is strengthened by multiple comparisons of different treatments via NMA, generalization of our results is still limited by the potential bias resulting from the funding sources within the included RCTs. Similarly, the main findings regarding the efficacy of EPA/DHA in migraine prophylaxis primarily came from the few RCTs using EPA/DHA products [18,21,22,26,32]. Therefore, clinicians should avoid overinterpretation of the findings in the present NMA and apply them in a relatively conservative way.

## Conclusions

This NMA suggests that prophylactic EPA/DHA supplementations are associated with better reduction of the frequency and severity of migraine episodes and fair acceptability. In addition, these beneficial effects were not inferior to those of current pharmacologic regimens approved by the FDA or treatment guidelines. Our findings provide a rationale for designing future large-scale RCTs to investigate optimal dosing of EPA/DHA supplementation in migraine patients. However, because of the numbers of RCTs using EPA/DHA products, clinicians should avoid overinterpretation of the findings of the present NMA and apply them in a relatively conservative way.

### Author contributions

The authors’ responsibilities were as follows – P-TT, B-YZ, J-JC, C-HK, B-SZ: contributed equally as first authors, performed the literature search, article screening and selection, data extraction, data analysis, and manuscript drafting; JSK, Y-SC, C-KS, Y-CW, Y-KT, BS, AFC, C-SL, T-YC, C-WH, M-WS, C-PY, S-PH, Y-WC: contributed to the study design, concept formation, data extraction, literature screen, data curation, and manuscript revision; Y-LS, C-MH, K-PS, P-YL: contributed equally as senior corresponding authors, took responsibility for manuscript revision, data curation, data analysis, concept formation, and manuscript submission; P-TT, B-SZ: had full access to all the data and take responsibility for the integrity of the data and the accuracy of the data analysis; and all authors: read and approved the final version.

### Conflict of interest

The authors report no conflicts of interest.

### Funding

The authors were supported by the following grants: Brendon Stubbs is supported by a Clinical Lectureship (ICA-CL-2017-03-001) jointly funded by Health Education England and the National Institute for Health Research (NIHR). Brendon Stubbs is part funded by the NIHR Biomedical Research Centre at South London and Maudsley NHS Foundation Trust. Brendon Stubbs is also supported by the Maudsley Charity, King’s College London. Kuan-Pin Su is supported by the MOST 109-2320-B-038-057-MY3, 109-2320-B-039-066, 110-2321-B-006-004, 110-2811-B-039-507, 110-2320-B-039-048-MY2, and 110-2320-B-039-047-MY3 from the Ministry of Science and Technology, Taiwan; ANHRF 109-31, 109-40, 110-13, 110-26, 110-44, and 110-45 from An-Nan Hospital, China Medical University, Tainan, Taiwan; CMRC-CMA-2 from Higher Education Sprout Project by the Ministry of Education (MOE), Taiwan; CMU 110-AWARD-02, CMU108-SR-106 from the China Medical University, Taichung, Taiwan; and CRS-108-048, DMR-102-076, DMR-103-084, DMR-106-225, DMR-107-204, DMR-108-216, DMR-109-102, DMR-109-244, DMR-HHC-109-11, DMR-HHC-109-12, DMR-HHC-110-10, and DMR-110-124 from the China Medical University Hospital, Taichung, Taiwan. John S. Kuo is partly supported by a Yu Shan Scholar award from the MOE, Taiwan. This article presents independent research. The views expressed in this publication are those of the authors and not necessarily those of the acknowledged institutions. None of the above funders played any roles in the design and conduct of the study, collection, management, analysis, and interpretation of the data, preparation, review, or approval of the manuscript, and decision to submit the manuscript for publication.

## Appendix A Supplementary data

Multimedia component1 Multimedia component1
