# Supplementary material for: High Dosage Omega-3 Fatty Acids Outperform Existing Pharmacological Options for Migraine Prophylaxis: A Network Meta-Analysis
Source: Adv Nutr. 2023 Dec 16;15(2):100163. doi: 10.1016/j.advnut.2023.100163 (PMC10808921; doi:10.1016/j.advnut.2023.100163)
Supplement: Multimedia component1 [file mmc1.pdf]

Supplement tables and figures

Efficacy of high dosage anti-inflammatory EPA/DHA for migraine prophylaxis: a network meta-analysis

Ping-Tao Tseng

eTable 1: PRISMA 2020 checklist of the current network meta-analysis

Section and Topic	Item #	Checklist item	Page where item is reported
**TITLE**			
Title	1	Identify the report as a systematic review.	1
**ABSTRACT**			
Abstract	2	See the PRISMA 2020 for Abstracts checklist.	7-8
**INTRODUCTION**			
Rationale	3	Describe the rationale for the review in the context of existing knowledge.	9-10
Objectives	4	Provide an explicit statement of the objective(s) or question(s) the review addresses.	9-10
**METHODS**			
Eligibility criteria	5	Specify the inclusion and exclusion criteria for the review and how studies were grouped for the syntheses.	11-12
Information sources	6	Specify all databases, registers, websites, organisations, reference lists and other sources searched or consulted to identify studies. Specify the date when each source was last searched or consulted.	11-12
Search strategy	7	Present the full search strategies for all databases, registers and websites, including any filters and limits used.	11-12
Selection process	8	Specify the methods used to decide whether a study met the inclusion criteria of the review, including how many reviewers screened each record and each report retrieved, whether they worked independently, and if applicable, details of automation tools used in the process.	11-12
Data collection process	9	Specify the methods used to collect data from reports, including how many reviewers collected data from each report, whether they worked independently, any processes for obtaining or confirming data from study investigators, and if applicable, details of automation tools used in the process.	11-12
Data items	10a	List and define all outcomes for which data were sought. Specify whether all results that were compatible with each outcome domain in each study were sought (e.g. for all measures, time points, analyses), and if not, the methods used to decide which results to collect.	12-13
	10b	List and define all other variables for which data were sought (e.g. participant and intervention characteristics, funding sources). Describe any assumptions made about any missing or unclear information.	12-13
Study risk of bias assessment	11	Specify the methods used to assess risk of bias in the included studies, including details of the tool(s) used, how many reviewers assessed each study and whether they worked independently, and if applicable, details of automation tools used in the process.	12-13
Effect measures	12	Specify for each outcome the effect measure(s) (e.g. risk ratio, mean difference) used in the synthesis or presentation of results.	12-13
Synthesis methods	13a	Describe the processes used to decide which studies were eligible for each synthesis (e.g. tabulating the study intervention characteristics and comparing against the planned groups for each synthesis (item #5)).	12-13
	13b	Describe any methods required to prepare the data for presentation or synthesis, such as handling of missing summary statistics, or data conversions.	13-14
	13c	Describe any methods used to tabulate or visually display results of individual studies and syntheses.	13-14
	13d	Describe any methods used to synthesize results and provide a rationale for the choice(s). If meta-analysis was performed, describe the model(s), method(s) to identify the presence and extent of statistical heterogeneity, and software package(s) used.	13-14
	13e	Describe any methods used to explore possible causes of heterogeneity among study results (e.g. subgroup analysis, meta-regression).	13-14
	13f	Describe any sensitivity analyses conducted to assess robustness of the synthesized results.	13-14
Reporting bias assessment	14	Describe any methods used to assess risk of bias due to missing results in a synthesis (arising from reporting biases).	13-14
Certainty assessment	15	Describe any methods used to assess certainty (or confidence) in the body of evidence for an outcome.	13-14
**RESULTS**			
Study selection	16a	Describe the results of the search and selection process, from the number of records identified in the search to the number of studies included in the review, ideally using a flow diagram.	15-16, Fig 1
	16b	Cite studies that might appear to meet the inclusion criteria, but which were excluded, and explain why they were excluded.	15-16, eTab 2

Section and Topic	Item #	Checklist item	Page where item is reported
Study characteristics	17	Cite each included study and present its characteristics.	15-16, eTab 3
Risk of bias in studies	18	Present assessments of risk of bias for each included study.	15-16, eFig 3
Results of individual studies	19	For all outcomes, present, for each study: (a) summary statistics for each group (where appropriate) and (b) an effect estimate and its precision (e.g. confidence/credible interval), ideally using structured tables or plots.	15-16, eTab 3
Results of syntheses	20a	For each synthesis, briefly summarise the characteristics and risk of bias among contributing studies.	16-17, eFig 4
	20b	Present results of all statistical syntheses conducted. If meta-analysis was done, present for each the summary estimate and its precision (e.g. confidence/credible interval) and measures of statistical heterogeneity. If comparing groups, describe the direction of the effect.	16-17, Fig 3, eFig 3
	20c	Present results of all investigations of possible causes of heterogeneity among study results.	16-17, eTab 6-7
	20d	Present results of all sensitivity analyses conducted to assess the robustness of the synthesized results.	16-17
Reporting biases	21	Present assessments of risk of bias due to missing results (arising from reporting biases) for each synthesis assessed.	16-17, eFig 4
Certainty of evidence	22	Present assessments of certainty (or confidence) in the body of evidence for each outcome assessed.	17-18, eTab 6-7
**DISCUSSION**			
Discussion	23a	Provide a general interpretation of the results in the context of other evidence.	19-22
	23b	Discuss any limitations of the evidence included in the review.	23
	23c	Discuss any limitations of the review processes used.	23
	23d	Discuss implications of the results for practice, policy, and future research.	24
**OTHER INFORMATION**			
Registration and protocol	24a	Provide registration information for the review, including register name and registration number, or state that the review was not registered.	8
	24b	Indicate where the review protocol can be accessed, or state that a protocol was not prepared.	8
	24c	Describe and explain any amendments to information provided at registration or in the protocol.	8
Support	25	Describe sources of financial or non-financial support for the review, and the role of the funders or sponsors in the review.	25
Competing interests	26	Declare any competing interests of review authors.	25
Availability of data, code and other materials	27	Report which of the following are publicly available and where they can be found: template data collection forms; data extracted from included studies; data used for all analyses; analytic code; any other materials used in the review.	25

The current checklist followed the latest PRISMA 2020 guideline.(1)

eTable 2: Keyword in each database and search result

Part of omega-3 fatty acid

Database	Keyword	Filter	Date	Result
PubMed	(omega 3 OR essential fatty acid OR eicosapentaenoic acid OR EPA OR DHA OR docosahexaenoic acid OR omega 6 OR fatty acid OR PUFA OR polyunsaturated fatty acid OR linoleic acid OR arachidonic acid OR gamma-linolenic acid OR dihomo-gamma-linolenic acid OR stearidonic acid OR heneicosapentaenoic acid OR docosapentaenoic acid OR clupanodonic acid OR hypogeic acid OR oleic acid OR elaidic acid OR gondoic acid OR omega 3 fatty acid[MeSH Terms] OR omega 6 fatty acid[MeSH Terms] OR omega 9 fatty acid[MeSH Terms]) AND (migraine OR migrain* OR migraine disorder) AND (random OR randomized OR randomised)	N/A	March 20th, 2022	141
Embase	(omega 3 OR essential fatty acid OR eicosapentaenoic acid OR EPA OR DHA OR docosahexaenoic acid OR omega 6 OR fatty acid OR PUFA OR polyunsaturated fatty acid OR linoleic acid OR arachidonic acid OR gamma-linolenic acid OR dihomo-gamma-linolenic acid OR stearidonic acid OR heneicosapentaenoic acid OR docosapentaenoic acid OR clupanodonic acid OR hypogeic acid OR oleic acid OR elaidic acid OR gondoic acid OR omega 3 fatty acid OR omega 6 fatty acid OR omega 9 fatty acid) AND (migraine OR migrain* OR migraine disorder) AND (random OR randomized OR randomised)	N/A	March 20th, 2022	140
ProQuest	(omega 3 OR essential fatty acid OR eicosapentaenoic acid OR EPA OR DHA OR	N/A	March 20th, 2022	3677

	docosahexaenoic acid OR omega 6 OR fatty acid OR PUFA OR polyunsaturated fatty acid OR linoleic acid OR arachidonic acid OR gamma-linolenic acid OR dihomo-gamma-linolenic acid OR stearidonic acid OR heneicosapentaenoic acid OR docosapentaenoic acid OR clupanodonic acid OR hypogeic acid OR oleic acid OR elaidic acid OR gondoic acid OR omega 3 fatty acid OR omega 6 fatty acid OR omega 9 fatty acid) AND (migraine OR migrain* OR migraine disorder) AND (random OR randomized OR randomised)			
ClinicalKey	(omega 3 OR essential fatty acid OR eicosapentaenoic acid OR EPA OR DHA OR docosahexaenoic acid OR omega 6 OR fatty acid OR PUFA OR polyunsaturated fatty acid OR linoleic acid OR arachidonic acid OR gamma-linolenic acid OR dihomo-gamma-linolenic acid OR stearidonic acid OR heneicosapentaenoic acid OR docosapentaenoic acid OR clupanodonic acid OR hypogeic acid OR oleic acid OR elaidic acid OR gondoic acid OR omega 3 fatty acid OR omega 6 fatty acid OR omega 9 fatty acid) AND (migraine OR migrain* OR migraine disorder) AND (random OR randomized OR randomised)	N/A	March 20th, 2022	1
Cochrane CENTRAL	(omega 3 OR essential fatty acid OR eicosapentaenoic acid OR EPA OR DHA OR docosahexaenoic acid OR omega 6 OR fatty acid OR PUFA OR polyunsaturated fatty acid OR linoleic acid OR arachidonic acid OR gamma-linolenic acid OR dihomo-gamma-linolenic acid OR stearidonic acid OR heneicosapentaenoic acid OR docosapentaenoic acid OR clupanodonic acid OR hypogeic acid OR oleic acid OR elaidic acid OR gondoic acid OR omega 3 fatty acid OR omega 6 fatty acid OR omega 9 fatty acid) AND (migraine OR migrain* OR migraine	N/A	March 20th, 2022	47

	disorder) AND (random OR randomized OR randomised)			
ScienceDirect	(omega 3 fatty acid OR omega 6 fatty acid OR omega 9 fatty acid) AND (migraine OR migraine disorder) AND (random OR randomized OR randomised)	N/A	March 20th, 2022	607
Web of Science	(omega 3 fatty acid OR omega 6 fatty acid OR omega 9 fatty acid) AND (migraine OR migraine disorder) AND (random OR randomized OR randomised)	N/A	March 20th, 2022	17
ClinicalTrials.gov	(omega 3 fatty acid OR omega 6 fatty acid OR omega 9 fatty acid) AND (migraine OR migraine disorder) AND (random OR randomized OR randomised)	N/A	March 20th, 2022	1

Part of approval oral medication

Database	Keyword	Filter	Date	Result
PubMed	(topiramate OR topamax OR valproate OR valproic acid OR propranolol OR timolol OR amitriptyline OR venlafaxine OR lisinopril OR frovatriptan OR candesartan) AND (migraine) AND (placebo) AND (random OR randomized OR randomised)	Randomized controlled trials	March 20th, 2022	122
Embase	(topiramate OR topamax OR valproate OR valproic acid OR propranolol OR timolol OR amitriptyline OR venlafaxine OR lisinopril OR frovatriptan OR candesartan) AND (migraine) AND (placebo) AND (random OR randomized OR randomised)	N/A	March 20th, 2022	709
ProQuest	(topiramate OR topamax OR valproate OR valproic acid OR propranolol OR timolol OR amitriptyline OR venlafaxine OR lisinopril OR frovatriptan OR candesartan) AND (migraine) AND (placebo)	N/A	March 20th, 2022	3

	AND (random OR randomized OR randomised)			
ClinicalKey	(topiramate OR topamax OR valproate OR valproic acid OR propranolol OR timolol OR amitriptyline OR venlafaxine OR lisinopril OR frovatriptan OR candesartan) AND (migraine) AND (placebo) AND (random OR randomized OR randomised)	N/A	March 20th, 2022	321
Cochrane CENTRAL	(topiramate OR topamax OR valproate OR valproic acid OR propranolol OR timolol OR amitriptyline OR venlafaxine OR lisinopril OR frovatriptan OR candesartan) AND (migraine) AND (placebo) AND (random OR randomized OR randomised)	N/A	March 20th, 2022	1310
ScienceDirect	(migraine) AND (placebo) AND (random OR randomized OR randomised)	Research article	March 20th, 2022	3353
Web of Science	(topiramate OR topamax OR valproate OR valproic acid OR propranolol OR timolol OR amitriptyline OR venlafaxine OR lisinopril OR frovatriptan OR candesartan) AND (migraine) AND (placebo) AND (random OR randomized OR randomised) randomised)	N/A	March 20th, 2022	403
ClinicalTrials.gov	(topiramate OR topamax OR valproate OR valproic acid OR propranolol OR timolol OR amitriptyline OR venlafaxine OR lisinopril OR frovatriptan OR candesartan) AND (migraine) AND (placebo) AND (random OR randomized OR randomised)	N/A	March 20th, 2022	11

eTable 3: Excluded studies and reason

Reason	Numbers	References
Acute treatment but not prevention	4	(2-6)
Chronic headache but not migraine	1	(7)
Duplicate sample source with another included studies	5	(8-12)
Focusing fasting-induced migraine, which is completely different from the other migraines	1	(13)
Focusing menstrual migraine, which is completely different from the other migraines	5	(14-18)
Lack of sufficient data to extract	5	(19-23)
Low lipid diet but not specific to omega 3 or omega 6	1	(24)
Meta-analysis	2	(25, 26)
Not oral form regimen	4	(27-30)
Not placebo control	2	(31, 32)
Not polyunsaturated fatty acid (alpha-lipoic acid is not a polyunsaturated fatty acid)	3	(33-35)
Not randomized controlled trial	1	(36)
Not report target outcome	1	(37)
Study protocol but not study result	2	(38, 39)

eTable 4A: League table of changes of frequency of migraine attack: adult patients

HighPUFA													***-1.36 (-2.07,-0.64)**
-0.27 (-1.02,0.48)	Val												***-1.10 (-1.48,-0.72)**
-0.33 (-1.31,0.64)	-0.06 (-0.77,0.64)	AmLowPUFA		-0.54 (-1.11,0.02)									
-0.61 (-1.55,0.33)	-0.34 (-0.99,0.31)	-0.28 (-1.18,0.63)	Ven										***-0.75 (-1.35,-0.14)**
***-0.88 (-1.68,-0.08)**	***-0.61 (-1.04,-0.18)**	-0.54 (-1.11,0.02)	-0.27 (-0.98,0.44)	Ami									***-0.48 (-0.84,-0.11)**
***-0.97 (-1.75,-0.19)**	***-0.70 (-1.10,-0.30)**	-0.63 (-1.38,0.11)	-0.36 (-1.05,0.33)	-0.09 (-0.58,0.40)	Cyc		-0.03 (-0.37,0.30)						-0.36 (-0.76,0.04)
***-0.98 (-1.86,-0.10)**	***-0.71 (-1.27,-0.15)**	-0.65 (-1.49,0.20)	-0.37 (-1.16,0.42)	-0.10 (-0.73,0.53)	-0.01 (-0.62,0.59)	Lis							-0.38 (-0.89,0.14)
***-0.99 (-1.72,-0.26)**	***-0.72 (-1.00,-0.44)**	-0.65 (-1.34,0.03)	-0.38 (-1.01,0.25)	-0.11 (-0.51,0.29)	-0.02 (-0.33,0.29)	-0.01 (-0.54,0.53)	Pro		-0.02 (-0.38,0.35)	-0.10 (-0.30,0.10)			***-0.31 (-0.48,-0.13)**
***-1.00 (-1.81,-0.19)**	***-0.73 (-1.18,-0.28)**	-0.67 (-1.44,0.10)	-0.39 (-1.11,0.33)	-0.12 (-0.66,0.41)	-0.03 (-0.53,0.46)	-0.02 (-0.66,0.62)	-0.01 (-0.42,0.39)	TPr		-0.02 (-0.39,0.36)			
***-1.02 (-1.78,-0.25)**	***-0.75 (-1.11,-0.39)**	-0.68 (-1.41,0.04)	-0.41 (-1.08,0.26)	-0.14 (-0.60,0.32)	-0.05 (-0.46,0.36)	-0.04 (-0.62,0.54)	-0.03 (-0.32,0.26)	-0.02 (-0.49,0.46)	Can				***-0.32 (-0.62,-0.02)**
***-1.02 (-1.74,-0.30)**	***-0.75 (-1.00,-0.50)**	***-0.68 (-1.36,-0.01)**	-0.41 (-1.02,0.21)	-0.14 (-0.52,0.24)	-0.05 (-0.38,0.28)	-0.04 (-0.56,0.48)	-0.03 (-0.19,0.13)	-0.02 (-0.39,0.36)	-0.00 (-0.29,0.29)	Top		-0.30 (-0.67,0.07)	***-0.39 (-0.54,-0.24)**
***-1.06 (-1.83,-0.29)**	***-0.79 (-1.17,-0.41)**	-0.73 (-1.46,0.01)	-0.45 (-1.13,0.23)	-0.18 (-0.66,0.29)	-0.09 (-0.54,0.35)	-0.08 (-0.68,0.51)	-0.07 (-0.41,0.27)	-0.06 (-0.55,0.43)	-0.04 (-0.45,0.37)	-0.04 (-0.36,0.28)	Max		-0.30 (-0.60,0.01)
***-1.12 (-1.90,-0.33)**	***-0.85 (-1.24,-0.45)**	***-0.78 (-1.52,-0.04)**	-0.51 (-1.19,0.18)	-0.24 (-0.72,0.25)	-0.15 (-0.60,0.31)	-0.14 (-0.74,0.47)	-0.13 (-0.48,0.23)	-0.11 (-0.61,0.38)	-0.10 (-0.52,0.33)	-0.10 (-0.42,0.22)	-0.06 (-0.50,0.39)	Lam	***-0.45 (-0.82,-0.08)**
***-1.36 (-2.07,-0.64)**	***-1.09 (-1.32,-0.85)**	***-1.02 (-1.69,-0.35)**	***-0.75 (-1.35,-0.14)**	***-0.48 (-0.84,-0.11)**	***-0.39 (-0.71,-0.06)**	-0.38 (-0.89,0.14)	***-0.37 (-0.52,-0.21)**	-0.35 (-0.74,0.03)	***-0.34 (-0.62,-0.06)**	***-0.34 (-0.43,-0.25)**	-0.30 (-0.60,0.01)	-0.24 (-0.56,0.08)	Pla

Pairwise (upper-right portion) and network (lower-left portion) meta-analysis results are presented as estimate effect sizes for the outcome of improvement in frequency of migraine attack. Interventions are reported in order of mean ranking of treatment effect, and outcomes are expressed as standardized mean difference (SMD) (95% confidence intervals). For the pairwise meta-analyses, SMD of less than 0 indicate that the treatment specified in the row got better improvement in frequency of migraine attack than that specified in the column. For the network meta-analysis (NMA), SMD of less than 0 indicate that the treatment specified in the column got better improvement in frequency of migraine attack than that specified in the row. Bold results marked with * indicate statistical significance.

eTable 4B: League table of changes of frequency of migraine attack: child patients

Ami	0.00 (-0.23,0.23)			-0.12 (-0.40,0.16)	
-0.04 (-0.44,0.36)	Top			-0.03 (-0.32,0.25)	
-0.03 (-1.08,1.03)	0.01 (-1.00,1.02)	VaLowPUFA			-0.08 (-0.86,0.71)
-0.07 (-1.00,0.86)	-0.03 (-0.92,0.85)	-0.05 (-1.33,1.23)	MedPUFA	0.00 (-0.76,0.76)	
-0.07 (-0.48,0.34)	-0.03 (-0.32,0.26)	-0.05 (-1.02,0.92)	0.00 (-0.83,0.83)	Pla	-0.03 (-0.30,0.24)
-0.10 (-0.71,0.50)	-0.06 (-0.60,0.47)	-0.08 (-0.94,0.79)	-0.03 (-0.98,0.92)	-0.03 (-0.48,0.41)	Val

Pairwise (upper-right portion) and network (lower-left portion) meta-analysis results are presented as estimate effect sizes for the outcome of improvement in frequency of migraine attack. Interventions are reported in order of mean ranking of treatment effect, and outcomes are expressed as standardized mean difference (SMD) (95% confidence intervals). For the pairwise meta-analyses, SMD of less than 0 indicate that the treatment specified in the row got better improvement in frequency of migraine attack than that specified in the column. For the network meta-analysis (NMA), SMD of less than 0 indicate that the treatment specified in the column got better improvement in frequency of migraine attack than that specified in the row. Bold results marked with * indicate statistical significance.

eTable 4C: League table of changes of frequency of migraine attack: episodic migraine

Val										***-0.79 (-1.54,-0.05)**
-0.01 (-1.05,1.03)	Ven									***-0.75 (-1.35,-0.14)**
-0.28 (-1.20,0.64)	-0.27 (-1.52,0.98)	Ami								***-0.48 (-0.84,-0.11)**
-0.34 (-1.33,0.66)	-0.32 (-1.63,0.98)	-0.05 (-1.27,1.16)	Can							-0.42 (-0.95,0.10)
-0.38 (-1.37,0.60)	-0.37 (-1.67,0.93)	-0.10 (-1.31,1.11)	-0.05 (-1.31,1.22)	Lis						-0.38 (-0.89,0.14)
-0.39 (-1.26,0.48)	-0.38 (-1.59,0.83)	-0.11 (-1.22,1.00)	-0.06 (-1.23,1.12)	-0.01 (-1.18,1.16)	Cyc	-0.03 (-0.37,0.30)				-0.36 (-0.76,0.04)
-0.42 (-1.10,0.25)	-0.41 (-1.50,0.67)	-0.14 (-1.11,0.83)	-0.09 (-1.13,0.95)	-0.04 (-1.07,0.99)	-0.03 (-0.78,0.71)	Pro		-0.10 (-0.30,0.10)		***-0.32 (-0.52,-0.12)**
-0.46 (-1.36,0.44)	-0.45 (-1.69,0.79)	-0.18 (-1.32,0.96)	-0.13 (-1.33,1.07)	-0.08 (-1.27,1.11)	-0.07 (-1.17,1.02)	-0.04 (-0.99,0.91)	Max			-0.30 (-0.60,0.01)
-0.49 (-1.00,0.02)	-0.47 (-1.47,0.52)	-0.21 (-1.07,0.66)	-0.15 (-1.09,0.79)	-0.10 (-1.04,0.83)	-0.09 (-0.89,0.70)	-0.06 (-0.62,0.49)	-0.02 (-0.86,0.82)	Top	-0.30 (-0.67,0.07)	***-0.28 (-0.43,-0.14)**
-0.55 (-1.39,0.29)	-0.54 (-1.73,0.66)	-0.27 (-1.36,0.82)	-0.21 (-1.37,0.94)	-0.17 (-1.31,0.98)	-0.16 (-1.20,0.88)	-0.13 (-1.01,0.75)	-0.09 (-1.16,0.98)	-0.06 (-0.78,0.66)	Lam	***-0.45 (-0.82,-0.08)**
***-0.76 (-1.18,-0.33)**	-0.75 (-1.70,0.20)	-0.48 (-1.29,0.34)	-0.42 (-1.32,0.48)	-0.38 (-1.27,0.52)	-0.37 (-1.12,0.39)	-0.34 (-0.86,0.19)	-0.30 (-1.09,0.50)	-0.27 (-0.55,0.01)	-0.21 (-0.93,0.51)	Pla

Pairwise (upper-right portion) and network (lower-left portion) meta-analysis results are presented as estimate effect sizes for the outcome of improvement in frequency of migraine attack. Interventions are reported in order of mean ranking of treatment effect, and outcomes are expressed as standardized mean difference (SMD) (95% confidence intervals). For the pairwise meta-analyses, SMD of less than 0 indicate that the treatment specified in the row got better improvement in frequency of migraine attack than that specified in the column. For the network meta-analysis (NMA), SMD of less than 0 indicate that the treatment specified in the column got better improvement in frequency of migraine attack than that specified in the row. Bold results marked with * indicate statistical significance.

eTable 4D: League table of changes of frequency of migraine attack: chronic migraine

HighPUFA					***-1.36 (-2.07,-0.64)**
-0.23 (-2.34,1.89)	Val				***-1.13 (-1.63,-0.62)**
-0.54 (-2.31,1.23)	-0.31 (-2.01,1.38)	Top	0.02 (-0.36,0.39)		***-0.78 (-1.50,-0.06)**
-0.53 (-2.79,1.74)	-0.30 (-2.51,1.91)	0.02 (-1.40,1.43)	TPr		
-1.36 (-3.54,0.83)	-1.13 (-3.26,1.00)	-0.81 (-2.60,0.97)	-0.83 (-3.11,1.45)	MedPUFA	0.00 (-0.76,0.76)
-1.36 (-2.89,0.18)	-1.13 (-2.58,0.33)	-0.81 (-1.69,0.06)	-0.83 (-2.49,0.83)	-0.00 (-1.56,1.56)	Pla

Pairwise (upper-right portion) and network (lower-left portion) meta-analysis results are presented as estimate effect sizes for the outcome of improvement in frequency of migraine attack. Interventions are reported in order of mean ranking of treatment effect, and outcomes are expressed as standardized mean difference (SMD) (95% confidence intervals). For the pairwise meta-analyses, SMD of less than 0 indicate that the treatment specified in the row got better improvement in frequency of migraine attack than that specified in the column. For the network meta-analysis (NMA), SMD of less than 0 indicate that the treatment specified in the column got better improvement in frequency of migraine attack than that specified in the row. Bold results marked with * indicate statistical significance.

eTable 4E: League table of response rate

AmLowPUFA										***31.25 (6.54142.86)**		
5.59 (0.52,60.50)	ToN			***3.83 (1.17,12.50)**				***5.81 (1.81,18.87)**				
***12.12 (1.55,94.90)**	2.17 (0.37,12.70)	Can			1.13 (0.54,2.36)							6.10 (0.85,43.72)
***18.58 (2.12,163.06)**	3.33 (0.52,21.39)	1.53 (0.35,6.67)	TPr	1.15 (0.59,2.24)								
***21.36 (3.32,137.46)**	3.82 (0.86,16.92)	1.76 (0.68,4.58)	1.15 (0.38,3.52)	Top	0.75 (0.50,1.13)			1.52 (0.40,5.78)		1.28 (0.93,1.76)	2.04 (0.97,4.32)	***2.47 (1.69,3.61)**
***21.43 (3.19,144.12)**	3.84 (0.78,18.90)	1.77 (0.69,4.54)	1.15 (0.33,4.06)	1.00 (0.56,1.79)	Pro		0.87 (0.31,2.43)		1.25 (0.66,2.36)	1.33 (0.47,3.82)		***2.26 (1.56,3.27)**
***22.40 (2.62,191.53)**	4.01 (0.62,26.00)	1.85 (0.45,7.62)	1.21 (0.25,5.93)	1.05 (0.34,3.26)	1.05 (0.31,3.47)	Max						***2.31 (1.27,4.21)**
***23.77 (3.51,160.79)**	4.26 (0.87,20.93)	1.96 (0.71,5.39)	1.28 (0.36,4.50)	1.11 (0.63,1.97)	1.11 (0.57,2.15)	1.06 (0.33,3.47)	Val					***2.22 (1.20,4.11)**
***32.56 (2.78,380.76)**	***5.83 (1.33,25.45)**	2.69 (0.41,17.41)	1.75 (0.25,12.42)	1.52 (0.31,7.60)	1.52 (0.28,8.38)	1.45 (0.20,10.38)	1.37 (0.25,7.54)	Nor				
***31.84 (3.89,260.26)**	5.70 (0.92,35.22)	2.63 (0.70,9.81)	1.71 (0.37,7.96)	1.49 (0.52,4.27)	1.49 (0.55,4.03)	1.42 (0.32,6.24)	1.34 (0.44,4.03)	0.98 (0.14,6.67)	Cyc			1.32 (0.64,2.72)
***30.80 (5.13,184.91)**	***5.51 (1.15,26.49)**	2.54 (0.92,6.98)	1.66 (0.49,5.65)	1.44 (0.87,2.38)	1.44 (0.75,2.75)	1.38 (0.42,4.48)	1.30 (0.67,2.52)	0.95 (0.18,5.10)	0.97 (0.32,2.90)	Ami		1.44 (0.78,2.68)
***35.37 (4.29,291.61)**	***6.33 (1.04,38.58)**	2.92 (0.74,11.46)	1.90 (0.42,8.70)	1.66 (0.59,4.62)	1.65 (0.53,5.16)	1.58 (0.36,7.02)	1.49 (0.48,4.61)	1.09 (0.16,7.32)	1.11 (0.26,4.67)	1.15 (0.38,3.49)	Lam	1.82 (0.85,3.90)
***51.72 (8.10,330.29)**	***9.26 (2.01,42.56)**	***4.27 (1.71,10.66)**	2.78 (0.86,8.97)	***2.42 (1.72,3.40)**	***2.41 (1.43,4.07)**	2.31 (0.78,6.80)	***2.18 (1.34,3.52)**	1.59 (0.31,8.21)	1.62 (0.59,4.46)	***1.68 (1.04,2.70)**	1.46 (0.52,4.09)	Pla

Pairwise (upper-right portion) and network (lower-left portion) meta-analysis results are presented as estimate effect sizes for the outcome of response rate. Interventions are reported in order of mean ranking of treatment effect, and outcomes are expressed as odds ratio (OR) (95% confidence intervals). For the pairwise meta-analyses, OR of more than 1 indicate that the treatment specified in the row got better response rate than that specified in the column. For the network meta-analysis (NMA), OR of more than 1 indicate that the treatment specified in the column got better response rate than that specified in the row. Bold results marked with * indicate statistical significance.

eTable 4F: League table of changes of severity of migraine

HighPUFA													***-2.23 (-3.06,-1.41)**
-0.95 (-2.28,0.38)	MedPUFA												***-1.28 (-2.12,-0.44)**
***-1.23 (-2.38,-0.08)**	-0.28 (-1.43,0.88)	Val	-0.02 (-0.80,0.77)										***-1.00 (-1.50,-0.51)**
-1.25 (-2.70,0.21)	-0.29 (-1.76,1.17)	-0.02 (-0.91,0.88)	VaLowPUFA										
***-1.52 (-2.72,-0.32)**	-0.57 (-1.77,0.64)	-0.29 (-1.29,0.71)	-0.27 (-1.62,1.07)	Ven									***-0.71 (-1.32,-0.11)**
***-1.91 (-2.88,-0.94)**	-0.95 (-1.94,0.03)	-0.68 (-1.39,0.03)	-0.66 (-1.81,0.48)	-0.39 (-1.18,0.40)	Top	-0.01 (-0.41,0.39)		-0.14 (-0.37,0.09)	-0.36 (-0.73,0.01)				***-0.31 (-0.58,-0.03)**
***-1.92 (-3.05,-0.78)**	-0.97 (-2.11,0.18)	-0.69 (-1.61,0.24)	-0.67 (-1.96,0.62)	-0.40 (-1.39,0.59)	-0.01 (-0.60,0.58)	TPr							
***-2.01 (-3.16,-0.86)**	-1.05 (-2.21,0.11)	-0.78 (-1.72,0.17)	-0.76 (-2.06,0.54)	-0.49 (-1.49,0.52)	-0.10 (-0.82,0.62)	-0.09 (-1.02,0.84)	Lis						-0.23 (-0.74,0.28)
***-2.03 (-3.03,-1.03)**	***-1.08 (-2.09,-0.06)**	***-0.80 (-1.55,-0.05)**	-0.78 (-1.96,0.39)	-0.51 (-1.34,0.32)	-0.12 (-0.51,0.26)	-0.11 (-0.82,0.59)	-0.02 (-0.78,0.74)	Ami					-0.17 (-0.54,0.20)
***-2.06 (-3.13,-1.00)**	***-1.11 (-2.19,-0.04)**	-0.83 (-1.67,0.00)	-0.82 (-2.05,0.41)	-0.54 (-1.45,0.36)	-0.16 (-0.67,0.35)	-0.15 (-0.93,0.64)	-0.06 (-0.90,0.78)	-0.03 (-0.64,0.57)	Lam				***-0.37 (-0.74,-0.00)**
***-2.08 (-3.11,-1.05)**	***-1.13 (-2.17,-0.08)**	***-0.85 (-1.65,-0.06)**	-0.83 (-2.03,0.36)	-0.56 (-1.43,0.31)	-0.17 (-0.68,0.33)	-0.16 (-0.94,0.62)	-0.07 (-0.87,0.73)	-0.05 (-0.61,0.51)	-0.02 (-0.69,0.66)	Can		0.00 (-0.36,0.36)	-0.14 (-0.49,0.22)
***-2.13 (-3.20,-1.06)**	***-1.17 (-2.25,-0.09)**	***-0.90 (-1.74,-0.06)**	-0.88 (-2.11,0.35)	-0.61 (-1.52,0.30)	-0.22 (-0.80,0.36)	-0.21 (-1.04,0.62)	-0.12 (-0.97,0.73)	-0.10 (-0.73,0.53)	-0.06 (-0.79,0.67)	-0.05 (-0.72,0.63)	Max		-0.11 (-0.39,0.17)
***-2.16 (-3.24,-1.08)**	***-1.20 (-2.29,-0.12)**	***-0.93 (-1.78,-0.08)**	-0.91 (-2.15,0.33)	-0.64 (-1.56,0.28)	-0.25 (-0.84,0.34)	-0.24 (-1.08,0.60)	-0.15 (-1.01,0.71)	-0.13 (-0.77,0.52)	-0.09 (-0.83,0.65)	-0.08 (-0.62,0.46)	-0.03 (-0.78,0.71)	Pro	0.00 (-0.36,0.36)
***-2.23 (-3.17,-1.30)**	***-1.28 (-2.23,-0.33)**	***-1.00 (-1.67,-0.34)**	-0.99 (-2.10,0.13)	-0.71 (-1.46,0.03)	***-0.32 (-0.58,-0.07)**	-0.31 (-0.96,0.33)	-0.23 (-0.90,0.44)	-0.20 (-0.56,0.15)	-0.17 (-0.68,0.34)	-0.15 (-0.59,0.29)	-0.11 (-0.63,0.41)	-0.08 (-0.61,0.46)	Pla

Pairwise (upper-right portion) and network (lower-left portion) meta-analysis results are presented as estimate effect sizes for the outcome of improvement in severity of migraine. Interventions are reported in order of mean ranking of treatment effect, and outcomes are expressed as standardized mean difference (SMD) (95% confidence intervals). For the pairwise meta-analyses, SMD of less than 0 indicate that the treatment specified in the row got better improvement in severity of migraine than that specified in the column. For the network meta-analysis (NMA), SMD of less than 0 indicate that the treatment specified in the column got better improvement in severity of migraine than that specified in the row. Bold results marked with * indicate statistical significance.

eTable 4G: League table of rate of any adverse event

Pla	0.74 (0.35,1.55)	0.83 (0.39,1.75)	0.52 (0.18,1.56)	0.64 (0.17,2.41)	***0.38 (0.24,0.59)**		***0.33 (0.23,0.47)**			***0.27 (0.09,0.81)**		***0.03 (0.00,0.25)**
0.80 (0.35,1.83)	Can				0.71 (0.34,1.49)							
0.74 (0.42,1.31)	0.94 (0.35,2.52)	Val			1.91 (0.70,5.21)			0.28 (0.01,7.67)				
0.77 (0.23,2.63)	0.97 (0.23,4.07)	1.04 (0.28,3.88)	Cyc		0.59 (0.27,1.30)							
0.58 (0.15,2.28)	0.73 (0.15,3.61)	0.78 (0.18,3.42)	0.75 (0.12,4.69)	Lam			0.63 (0.21,1.89)					
0.57 (0.31,1.07)	0.72 (0.28,1.82)	0.77 (0.36,1.66)	0.74 (0.23,2.42)	0.99 (0.22,4.42)	Pro		0.45 (0.28,0.72)					
0.41 (0.07,2.28)	0.51 (0.08,3.45)	0.54 (0.09,3.34)	0.53 (0.06,4.32)	0.70 (0.08,6.09)	0.71 (0.12,4.35)	MedPUFA	0.83 (0.22,3.19)				***0.30 (0.10,0.93)**	
***0.34 (0.22,0.51)**	0.42 (0.17,1.07)	***0.45 (0.23,0.91)**	0.44 (0.12,1.57)	0.59 (0.15,2.29)	0.59 (0.29,1.19)	0.83 (0.16,4.45)	Top		0.70 (0.39,1.24)	0.46 (0.20,1.03)	0.36 (0.12,1.14)	
0.21 (0.01,6.94)	0.27 (0.01,9.58)	0.28 (0.01,8.90)	0.27 (0.01,10.96)	0.37 (0.01,15.60)	0.37 (0.01,12.61)	0.52 (0.01,25.60)	0.63 (0.02,21.03)	VaLowPUFA				
***0.24 (0.07,0.80)**	0.30 (0.07,1.30)	0.32 (0.08,1.22)	0.30 (0.05,1.71)	0.41 (0.07,2.43)	0.41 (0.11,1.59)	0.58 (0.08,4.45)	0.70 (0.22,2.21)	1.11 (0.03,45.01)	TPr			
***0.23 (0.11,0.46)**	***0.29 (0.10,0.86)**	***0.31 (0.13,0.76)**	0.30 (0.07,1.22)	0.40 (0.09,1.83)	0.40 (0.16,1.01)	0.57 (0.09,3.56)	0.68 (0.32,1.44)	1.09 (0.03,38.23)	0.98 (0.25,3.88)	Ami		
***0.12 (0.03,0.59)**	***0.15 (0.03,0.91)**	***0.16 (0.03,0.88)**	0.16 (0.02,1.16)	0.21 (0.03,1.64)	0.21 (0.04,1.14)	0.30 (0.07,1.36)	0.36 (0.08,1.66)	0.58 (0.01,26.67)	0.52 (0.08,3.52)	0.53 (0.10,2.91)	ToN	
***0.03 (0.00,0.31)**	***0.03 (0.00,0.44)**	***0.04 (0.00,0.44)**	***0.04 (0.00,0.53)**	***0.05 (0.00,0.76)**	***0.05 (0.00,0.58)**	0.07 (0.00,1.32)	***0.08 (0.01,0.94)**	0.13 (0.00,9.09)	0.12 (0.01,1.75)	0.12 (0.01,1.47)	0.23 (0.01,4.02)	Ven

Pairwise (upper-right portion) and network (lower-left portion) meta-analysis results are presented as estimate effect sizes for the outcome of rate of any adverse event. Interventions are reported in order of mean ranking of tolerability, and outcomes are expressed as odds ratio (OR) (95% confidence intervals). For the pairwise meta-analyses, OR of less than 1 indicate that the treatment specified in the row got less rate of any adverse event than that specified in the column. For the network meta-analysis (NMA), OR of less than 1 indicate that the treatment specified in the column got less rate of any adverse event than that specified in the row. Bold results marked with * indicate statistical significance.

Abbreviation: 95%CI: 95% confidence interval; Ami: amitriptyline; AmLowPUFA: low dosage n3PUFA + amitriptyline; AMSTAR: assessing the methodological quality of systematic review; Bot: Botox-A; Can: candesartan; CGRP: calcitonin gene-related peptide; Cyc: cyclandelate; DHA: docosahexaenoic acid; EPA: eicosapentaenoic acid; ES: effect size; HighPUFA: high dosage n3PUFA; Lam: lamotrigine; Lis: lisinopril; Max: Maxepa (omega-3 polyunsaturated fatty acids, EPA/DHA: 180mg/120mg x 6 pills); MedPUFA: medium dosage n3PUFA; Mem: memantine; NAM: network meta-analysis; Nor: nortriptyline; OR: odds ratio; Pla: Placebo; PRISMA: Preferred Reporting Items for Systematic Reviews and Meta-Analyses; Pro: propranolol; PUFA: polyunsaturated fatty acid; RCT: randomized controlled trial; SMD: standardized mean difference; SUCRA: surface under the cumulative ranking curve; ToN: topiramate + nortriptyline; Top: topiramate; TPr: topiramate + propranolol; TVGT: trigeminal nerve-trigeminocervical complex-ventroposteromedial thalamic nucleus; Val: valproate; VaLowPUFA: low dosage n3PUFA + valproate; Ven: venlafaxine

eTable 5A: SUCRA of the frequency of migraine attack

Treatment	SUCRA
HighPUFA	8.4
Val	21.4
AmLowPUFA	25.6
VaLowPUFA	27.4
Ven	31.0
Lis	54.1
Cyc	55.6
Can	55.8
TPr	56.3
Pro	57.3
Top	57.8
Ami	58.8
Max	60.6
Lam	64.9
MedPUFA	76.9
Pla	88.1

Sorted by order of mean rank of changes of frequency of migraine attack (the former, the better improvement in frequency of migraine attack)

eTable 5B: SUCRA of the frequency of migraine attack: adult patients

Treatment	SUCRA
HighPUFA	5.4
Val	10.5
AmLowPUFA	14.4
Ven	28.1
Ami	46.6
Cyc	54.8
Lis	57.7
Pro	58.0
TPr	59.8
Can	62.3
Top	63.2
Max	68.1
Lam	72.7
Pla	98.3

Sorted by order of mean rank of changes of frequency of migraine attack (the former, the better improvement in frequency of migraine attack)

eTable 5C: SUCRA of the frequency of migraine attack: child patients

Treatment	SUCRA
Ami	41.9
Top	46.7
VaLowPUFA	48.7
MedPUFA	50.3
Pla	54.7
Val	57.6

Sorted by order of mean rank of changes of frequency of migraine attack (the former, the better improvement in frequency of migraine attack)

eTable 5D: SUCRA of the frequency of migraine attack: episodic migraine

Treatment	SUCRA
Val	18.7
Ven	25.6
Ami	42.1
Can	47.5
Lis	47.8
Cyc	49.9
Pro	54.3
Max	56.1
Top	58.8
Lam	62.9
Pla	86.4

Sorted by order of mean rank of changes of frequency of migraine attack (the former, the better improvement in frequency of migraine attack)

eTable 5E: SUCRA of the frequency of migraine attack: chronic migraine

Treatment	SUCRA
HighPUFA	24.2
Val	31.6
Top	41.5
TPr	41.5
MedPUFA	76.9
Pla	84.2

Sorted by order of mean rank of changes of frequency of migraine attack (the former, the better improvement in frequency of migraine attack)

eTable 5F: SUCRA of the drop-out rate

Treatment	SUCRA
AmLowPUFA	16.6
TPr	23.0
Pro	26.5
ToN	39.2
Ami	42.3
Pla	44.6
Nor	46.1
Max	47.4
Lam	48.7
HighPUFA	50.7
Top	55.9
Cyc	56.0
Can	70.0
Ven	75.3
Val	76.0
VaLowPUFA	81.9

Sorted by order of mean rank of drop-out rate (the former, the less drop-out rate)

eTable 5G: SUCRA of the response rate

Treatment	SUCRA
AmLowPUFA	1.0
ToN	12.7
Can	23.7
TPr	42.6
Top	45.9
Pro	47.5
Max	50.6
Val	54.1
Nor	67.4
Cyc	68.9
Ami	69.7
Lam	73.1
Pla	92.9

Sorted by order of mean rank of response rate (the former, the better response rate)

eTable 5H: SUCRA of the severity of migraine

Treatment	SUCRA
HighPUFA	1.2
MedPUFA	15.0
Val	21.0
VaLowPUFA	25.4
Ven	32.4
Top	52.0
TPr	56.0
Lis	62.5
Ami	65.7
Lam	67.0
Can	68.6
Max	72.1
Pro	75.9
Pla	85.4

Sorted by order of mean rank of improvement of severity of migraine (the former, the better improvement of severity of migraine)

eTable 5I: SUCRA of the rate of any adverse event

Treatment	SUCRA
Pla	11.8
Can	24.1
Val	25.6
Cyc	27.4
Lam	36.4
Pro	37.6
MedPUFA	49.3
Top	59.3
VaLowPUFA	60.4
TPr	68.1
Ami	71.5
ToN	83.5
Ven	95.1

Sorted by order of mean rank of rate of any adverse event (the former, the less rate of any adverse event)

Abbreviation: 95%CI: 95% confidence interval; Ami: amitriptyline; AmLowPUFA: low dosage n3PUFA + amitriptyline; AMSTAR: assessing the methodological quality of systematic review; Bot: Botox-A; Can: candesartan; CGRP: calcitonin gene-related peptide; Cyc: cyclandelate; DHA: docosahexaenoic acid; EPA: eicosapentaenoic acid; ES: effect size; HighPUFA: high dosage n3PUFA; Lam: lamotrigine; Lis: lisinopril; Max: Maxepa (omega-3 polyunsaturated fatty acids, EPA/DHA: 180mg/120mg x 6 pills); MedPUFA: medium dosage n3PUFA; Mem: memantine; NAM: network meta-analysis; Nor: nortriptyline; OR: odds ratio; Pla: Placebo; PRISMA: Preferred Reporting Items for Systematic Reviews and Meta-Analyses; Pro: propranolol; PUFA: polyunsaturated fatty acid; RCT: randomized controlled trial; SMD: standardized mean difference; SUCRA: surface under the cumulative ranking curve; ToN: topiramate + nortriptyline; Top: topiramate; TPr: topiramate + propranolol; TVGT: trigeminal nerve-trigeminocervical complex-ventroposteromedial thalamic nucleus; Val: valproate; VaLowPUFA: low dosage n3PUFA + valproate; Ven: venlafaxine

eTable 6: Inconsistency of different intervention: design-by-treatment and loop inconsistency

Inconsistency	chi2	Prob>chi2
Changes of frequency of migraine attack		
design-by-treatment	1.20	0.9910
loop	0.02	0.8832
Drop-out rate		
design-by-treatment	8.11	0.5226
loop	1.20	0.5489
Response rate		
design-by-treatment	9.02	0.5301
loop	0.15	0.9849
Changes of severity of migraine		
design-by-treatment	4.82	0.3061
loop	0.00	0.9716
Frequency of any adverse event		
design-by-treatment	4.21	0.7551
loop	2.97	0.2267

eTable 7: Inconsistency of different intervention: side-splitting inconsistency

changes of frequency of migraine attack

Side	nosymmetric		symmetric		Treatments used	
	P>z	tau	P>z	tau		
A C *	0.998	0.33014	0.682	0.345848	A:	Pla
A E	.	.	0.956	0.345739	B:	AmLowPUFA
A F	.	.	.	.	C:	Ami
A H *	0.998	0.330139	0.998	0.330139	D:	TPr
A I	.	.	.	.	E:	Cyc
A J	.	.	0.259	0.322965	F:	Max
A K	.	.	.	.	G:	VaLowPUFA
A L	.	.	0.623	0.344455	H:	Val
A M	.	.	.	.	I:	MedPUFA
A N	.	.	0.923	0.346034	J:	Lam
A O *	0.999	0.330139	0.845	0.346046	K:	HighPUFA
A P	.	.	.	.	L:	Pro
B C *	0.998	0.330139	0.998	0.330139	M:	Lis
C O	0.682	0.345849	0.962	0.347315	N:	Can
D O *	0.999	0.330139	0.999	0.330139	O:	Top
E L *	0.956	0.345738	0.956	0.345738	P:	Ven
G H *	0.998	0.330139	0.998	0.330139		

J O *	0.259	0.322965	0.259	0.322965
L N	0.814	0.3431	0.951	0.345044
L O	0.678	0.346615	0.728	0.346489

Drop-out rate

Side	nosymmetric		symmetric		Treatments used	
	P>z	tau	P>z	tau		
A C	0.365	0.135732	0.428	0.136979	A:	Pla
A E	.	.	0.464	0.138842	B:	AmLowPUFA
A F	.	.	.	.	C:	Ami
A H	0.536	0.143866	0.536	0.143866	D:	TPr
A I	.	.	0.885	0.143389	E:	Cyc
A K	.	.	.	.	F:	Max
A L *	0.536	0.143866	0.01	1.61E-07	G:	VaLowPUFA
A N	.	.	0.447	0.138117	H:	Val
A O *	0.365	0.135732	0.58	0.14543	I:	Lam
A P	.	.	.	.	J:	ToN
B C *	1	0.140162	1	0.140162	K:	HighPUFA
C O	0.566	0.140821	0.492	0.138751	L:	Pro
D O *	1	0.140162	1	0.140162	M:	Nor
E L *	0.464	0.138842	0.464	0.138842	N:	Can

G H *	0.999	0.140162	0.999	0.140162	O:	Top
H L	0.536	0.143866	0.536	0.143866	P:	Ven
I O *	0.885	0.143389	0.885	0.143389		
J M	.	.	.	.		
J O *	1	0.140162	1	0.140162		
L N	0.898	0.141415	0.536	0.139015		
L O	0.012	3.13E-08	0.017	1.16E-08		
M O *	1	0.140162	1	0.140162		

Response rate

Side	nosymmetric		symmetric		Treatments used	
	P>z	tau	P>z	tau		
A C	0.981	0.48086	0.397	0.469959	A:	Pla
A E	.	.	0.468	0.469818	B:	AmLowPUFA
A F	.	.	.	.	C:	Ami
A G	.	.	0.465	0.460557	D:	TPr
A H	0.7	0.469643	0.7	0.469643	E:	Cyc
A K *	0.953	0.48333	0.957	0.480947	F:	Max
A L *	0.701	0.471067	0.612	0.477057	G:	Lam
A M	.	.	0.642	0.475354	H:	Val
B C *	0.997	0.458148	0.997	0.458148	I:	ToN

C K	0.173	0.453558	0.615	0.479263	J:	Nor
C L	0.907	0.473779	0.907	0.47378	K:	Top
D K *	0.999	0.458149	0.999	0.458148	L:	Pro
E L *	0.468	0.46982	0.468	0.469818	M:	Can
G K *	0.465	0.460557	0.465	0.460556		
H L	0.7	0.469643	0.7	0.469643		
I J	.	.	.	.		
I K *	0.998	0.458148	0.998	0.458148		
J K *	0.998	0.458148	0.998	0.458148		
K L	0.57	0.473296	0.487	0.47458		
L M	0.049	0.428059	0.205	0.460985		

Severity of migraine

Side	nosymmetric		symmetric		Treatments used	
	P>z	tau	P>z	tau		
A C	.	.	0.679	0.258002	A:	Pla
A D	.	.	0.041	0.131521	B:	TPr
A E	.	.	.	.	C:	Ami
A F	.	.	.	.	D:	Lam
A H *	0.997	0.222787	0.997	0.222787	E:	Ven
A I	.	.	.	.	F:	Max

A J *	0.999	0.222787	0.455	0.237437	G:	VaLowPUFA
A K	.	.	.	.	H:	Val
A L	.	.	0.422	0.232234	I:	MedPUFA
A M	.	.	.	.	J:	Top
A N	.	.	.	.	K:	HighPUFA
B J *	0.999	0.222787	0.999	0.222787	L:	Pro
C J	0.679	0.258002	0.928	0.261571	M:	Lis
D J *	0.041	0.131521	0.041	0.131522	N:	Can
G H *	0.997	0.222787	0.997	0.222787		
L N *	0.422	0.232234	0.422	0.232234		

Frequency of any adverse event

Side	nosymmetric		symmetric		Treatments used	
	P>z	tau	P>z	tau		
A C	0.463	0.524601	0.463	0.524601	A:	Pla
A D	.	.	0.364	0.514576	B:	TPr
A E	.	.	0.824	0.529529	C:	Ami
A H	0.122	0.478485	0.122	0.478485	D:	Cyc
A J	.	.	.	.	E:	Lam
A K *	0.463	0.524603	0.559	0.531847	F:	ToN
A L *	0.122	0.478485	0.012	0.381365	G:	VaLowPUFA

A M	.	.	0.592	0.528868	H:	Val
B K *	0.998	0.511772	0.998	0.511773	I:	MedPUFA
C K	0.463	0.524603	0.463	0.524602	J:	Ven
D L *	0.364	0.514576	0.364	0.514577	K:	Top
E K *	0.824	0.529529	0.824	0.529529	L:	Pro
F I	.	.	.	.	M:	Can
F K *	0.998	0.511773	0.998	0.511773		
G H *	0.999	0.511773	0.999	0.511773		
H L	0.122	0.478484	0.122	0.478486		
I K *	0.998	0.511773	0.998	0.511773		
K L	0.924	0.540728	0.517	0.526274		
L M	0.533	0.530016	0.989	0.540066		

eTable 8: Estimated between-studies standard deviations of different outcome

Outcome	Estimated between-studies standard deviation
Changes of frequency of migraine attack	0.33013918
Drop-out rate	0.14016212
Response rate	0.45814828
Severity of migraine	0.2227866
Frequency of any adverse event	0.5117725

eTable 9: Quality of evidence for primary outcome: changes of frequency of migraine attack and response rate

Changes of frequency of migraine attack

Comparisons	Direct evidence		Indirect evidence		Network meta-analysis	
	Standardized mean difference (95% CI)	The final rating of direct evidence	Co-efficiency (Standard error)	The final rating of indirect evidence	Standardized mean difference (95% CI)	Overall quality of evidence
HighPUFA vs Val					-0.54 (-1.56,0.49)	⊕◯◯◯Very low
HighPUFA vs AmLowPUFA					-0.48 (-1.85,0.89)	⊕◯◯◯Very low
HighPUFA vs VaLowPUFA					-0.46 (-1.91,0.98)	⊕◯◯◯Very low
HighPUFA vs Ven					-0.61 (-1.92,0.70)	⊕◯◯◯Very low
HighPUFA vs Lis					-0.98 (-2.25,0.29)	⊕◯◯◯Very low
HighPUFA vs Cyc					-0.98 (-2.16,0.19)	⊕◯◯◯Very low
HighPUFA vs Can					-1.01 (-2.11,0.08)	⊕◯◯◯Very low
HighPUFA vs TPr					-1.00 (-2.24,0.24)	⊕◯◯◯Very low
HighPUFA vs Pro					-1.01 (-2.05,0.03)	⊕◯◯◯Very low
HighPUFA vs Top					***-1.01 (-2.00,-0.03)**	⊕⊕⊕◯Medium
HighPUFA vs Ami					-1.02 (-2.10,0.05)	⊕◯◯◯Very low
HighPUFA vs Max					-1.06 (-2.26,0.14)	⊕◯◯◯Very low
HighPUFA vs Lam					-1.11 (-2.27,0.05)	⊕◯◯◯Very low
HighPUFA vs MedPUFA					-1.36 (-2.74,0.03)	⊕◯◯◯Very low
HighPUFA vs Pla	***-1.36 (-2.07,-0.64)**	⊕⊕⊕◯Medium			***-1.36 (-2.32,-0.39)**	⊕⊕⊕◯Medium
Val vs AmLowPUFA					0.06 (-0.98,1.10)	⊕◯◯◯Very low
Val vs VaLowPUFA	0.08 (-0.71,0.86)	⊕⊕◯◯Low	-1.63 (630.25)	⊕◯◯◯Very low	0.08 (-0.94,1.09)	⊕⊕⊕◯Medium

Val vs Ven					-0.07 (-1.03,0.88)	⊕⊕◯◯Low
Val vs Lis					-0.44 (-1.34,0.46)	⊕⊕◯◯Low
Val vs Cyc					-0.45 (-1.21,0.32)	⊕⊕◯◯Low
Val vs Can					-0.47 (-1.11,0.16)	⊕⊕◯◯Low
Val vs TPr					-0.46 (-1.31,0.39)	⊕⊕◯◯Low
Val vs Pro					-0.47 (-1.00,0.06)	⊕⊕◯◯Low
Val vs Top					***-0.47 (-0.89,-0.06)**	⊕⊕⊕◯Medium
Val vs Ami					-0.49 (-1.07,0.10)	⊕⊕◯◯Low
Val vs Max					-0.52 (-1.32,0.28)	⊕⊕◯◯Low
Val vs Lam					-0.57 (-1.32,0.17)	⊕⊕◯◯Low
Val vs MedPUFA					-0.82 (-1.87,0.24)	⊕◯◯◯Very low
Val vs Pla	***-0.86 (-1.49,-0.23)**	⊕⊕⊕◯Medium	0.03 (313.53)	⊕◯◯◯Very low	***-0.82 (-1.17,-0.46)**	⊕⊕⊕⊕High
AmLowPUFA vs VaLowPUFA					0.02 (-1.44,1.47)	⊕⊕◯◯Low
AmLowPUFA vs Ven					-0.13 (-1.45,1.19)	⊕⊕◯◯Low
AmLowPUFA vs Lis					-0.50 (-1.78,0.78)	⊕◯◯◯Very low
AmLowPUFA vs Cyc					-0.50 (-1.69,0.68)	⊕◯◯◯Very low
AmLowPUFA vs Can					-0.53 (-1.64,0.58)	⊕◯◯◯Very low
AmLowPUFA vs TPr					-0.52 (-1.76,0.72)	⊕◯◯◯Very low
AmLowPUFA vs Pro					-0.53 (-1.58,0.52)	⊕◯◯◯Very low
AmLowPUFA vs Top					-0.53 (-1.52,0.45)	⊕⊕◯◯Low
AmLowPUFA vs Ami	-0.54 (-1.11,0.02)	⊕⊕◯◯Low	-0.70 (642.77)	⊕◯◯◯Very low	-0.54 (-1.40,0.31)	⊕⊕⊕◯Medium
AmLowPUFA vs Max					-0.58 (-1.79,0.63)	⊕◯◯◯Very low
AmLowPUFA vs Lam					-0.63 (-1.80,0.54)	⊕◯◯◯Very low
AmLowPUFA vs MedPUFA					-0.88 (-2.27,0.52)	⊕◯◯◯Very low
AmLowPUFA vs Pla					-0.88 (-1.85,0.10)	⊕⊕◯◯Low
VaLowPUFA vs Ven					-0.15 (-1.54,1.25)	⊕◯◯◯Very low
VaLowPUFA vs Lis					-0.52 (-1.87,0.84)	⊕◯◯◯Very low
VaLowPUFA vs Cyc					-0.52 (-1.79,0.75)	⊕◯◯◯Very low
VaLowPUFA vs Can					-0.55 (-1.75,0.65)	⊕◯◯◯Very low

VaLowPUFA vs TPr			-0.54 (-1.86,0.79)	⊕◯◯◯Very low
VaLowPUFA vs Pro			-0.55 (-1.69,0.60)	⊕◯◯◯Very low
VaLowPUFA vs Top			-0.55 (-1.65,0.55)	⊕◯◯◯Very low
VaLowPUFA vs Ami			-0.56 (-1.74,0.61)	⊕◯◯◯Very low
VaLowPUFA vs Max			-0.60 (-1.89,0.69)	⊕◯◯◯Very low
VaLowPUFA vs Lam			-0.65 (-1.91,0.61)	⊕◯◯◯Very low
VaLowPUFA vs MedPUFA			-0.89 (-2.36,0.57)	⊕◯◯◯Very low
VaLowPUFA vs Pla			-0.89 (-1.97,0.18)	⊕◯◯◯Very low
Ven vs Lis			-0.37 (-1.58,0.84)	⊕◯◯◯Very low
Ven vs Cyc			-0.37 (-1.49,0.74)	⊕◯◯◯Very low
Ven vs Can			-0.40 (-1.43,0.63)	⊕◯◯◯Very low
Ven vs TPr			-0.39 (-1.57,0.79)	⊕◯◯◯Very low
Ven vs Pro			-0.40 (-1.37,0.57)	⊕⊕◯◯Low
Ven vs Top			-0.40 (-1.32,0.51)	⊕⊕◯◯Low
Ven vs Ami			-0.41 (-1.42,0.59)	⊕◯◯◯Very low
Ven vs Max			-0.45 (-1.59,0.69)	⊕◯◯◯Very low
Ven vs Lam			-0.50 (-1.60,0.60)	⊕◯◯◯Very low
Ven vs MedPUFA			-0.75 (-2.08,0.59)	⊕◯◯◯Very low
Ven vs Pla	***-0.75 (-1.35,-0.14)**	⊕⊕⊕◯Medium	-0.75 (-1.63,0.14)	⊕⊕◯◯Low
Lis vs Cyc			-0.00 (-1.07,1.06)	⊕◯◯◯Very low
Lis vs Can			-0.03 (-1.01,0.94)	⊕⊕◯◯Low
Lis vs TPr			-0.02 (-1.15,1.12)	⊕◯◯◯Very low
Lis vs Pro			-0.03 (-0.94,0.88)	⊕⊕◯◯Low
Lis vs Top			-0.03 (-0.88,0.82)	⊕⊕◯◯Low
Lis vs Ami			-0.04 (-0.99,0.91)	⊕⊕◯◯Low
Lis vs Max			-0.08 (-1.17,1.01)	⊕◯◯◯Very low
Lis vs Lam			-0.13 (-1.18,0.92)	⊕◯◯◯Very low
Lis vs MedPUFA			-0.38 (-1.67,0.92)	⊕◯◯◯Very low
Lis vs Pla	-0.38 (-0.89,0.14)	⊕⊕◯◯Low	-0.38 (-1.20,0.45)	⊕⊕⊕◯Medium

Cyc vs Can					-0.03 (-0.85,0.79)	⊕⊕◯◯Low
Cyc vs TPr					-0.01 (-1.04,1.01)	⊕◯◯◯Very low
Cyc vs Pro	-0.03 (-0.37,0.30)	⊕⊕◯◯Low	-0.02 (0.85)	⊕⊕◯◯Low	-0.03 (-0.69,0.64)	⊕⊕⊕◯Medium
Cyc vs Top					-0.03 (-0.73,0.67)	⊕⊕◯◯Low
Cyc vs Ami					-0.04 (-0.86,0.78)	⊕⊕◯◯Low
Cyc vs Max					-0.08 (-1.06,0.91)	⊕⊕◯◯Low
Cyc vs Lam					-0.13 (-1.06,0.81)	⊕⊕◯◯Low
Cyc vs MedPUFA					-0.37 (-1.57,0.83)	⊕◯◯◯Very low
Cyc vs Pla	-0.36 (-0.76,0.04)	⊕⊕◯◯Low	-0.41 (0.83)	⊕⊕◯◯Low	-0.37 (-1.05,0.30)	⊕⊕⊕◯Medium
Can vs TPr					0.01 (-0.92,0.95)	⊕⊕◯◯Low
Can vs Pro	0.02 (-0.35,0.38)	⊕⊕◯◯Low	-0.02 (0.48)	⊕⊕◯◯Low	0.00 (-0.57,0.58)	⊕⊕⊕◯Medium
Can vs Top					-0.00 (-0.56,0.56)	⊕⊕◯◯Low
Can vs Ami					-0.01 (-0.72,0.69)	⊕⊕◯◯Low
Can vs Max					-0.05 (-0.93,0.84)	⊕⊕◯◯Low
Can vs Lam					-0.10 (-0.93,0.74)	⊕⊕◯◯Low
Can vs MedPUFA					-0.34 (-1.47,0.78)	⊕◯◯◯Very low
Can vs Pla	***-0.32 (-0.62,-0.02)**	⊕⊕⊕◯Medium	-0.42 (0.84)	⊕⊕◯◯Low	-0.34 (-0.87,0.18)	⊕⊕⊕◯Medium
TPr vs Pro					-0.01 (-0.87,0.85)	⊕⊕◯◯Low
TPr vs Top	-0.02 (-0.39,0.36)	⊕⊕◯◯Low	-0.69 (617.30)	⊕◯◯◯Very low	-0.02 (-0.76,0.73)	⊕⊕⊕◯Medium
TPr vs Ami					-0.03 (-0.92,0.87)	⊕⊕◯◯Low
TPr vs Max					-0.06 (-1.12,0.99)	⊕◯◯◯Very low
TPr vs Lam					-0.11 (-1.11,0.88)	⊕◯◯◯Very low
TPr vs MedPUFA					-0.36 (-1.62,0.90)	⊕◯◯◯Very low
TPr vs Pla					-0.36 (-1.14,0.42)	⊕◯◯◯Very low
Pro vs Top	-0.10 (-0.30,0.10)	⊕⊕◯◯Low	-0.06 (0.28)	⊕⊕◯◯Low	-0.00 (-0.43,0.42)	⊕⊕⊕◯Medium
Pro vs Ami					-0.01 (-0.62,0.59)	⊕⊕◯◯Low
Pro vs Max					-0.05 (-0.87,0.77)	⊕⊕◯◯Low
Pro vs Lam					-0.10 (-0.86,0.65)	⊕⊕◯◯Low
Pro vs MedPUFA					-0.35 (-1.41,0.72)	⊕◯◯◯Very low
Pro vs Pla	***-0.31 (-0.48,-0.13)**	⊕⊕⊕◯Medium	-0.61 (0.57)	⊕⊕◯◯Low	-0.35 (-0.74,0.05)	⊕⊕⊕◯Medium

Top vs Ami	0.00 (-0.23,0.23)	⊕⊕◯◯Low	-0.02 (0.37)	⊕⊕◯◯Low	-0.01 (-0.50,0.48)	⊕⊕⊕◯Medium
Top vs Max					-0.05 (-0.79,0.70)	⊕⊕◯◯Low
Top vs Lam	-0.30 (-0.67,0.07)	⊕⊕◯◯Low	0.58 (0.69)	⊕⊕◯◯Low	-0.10 (-0.75,0.55)	⊕⊕⊕◯Medium
Top vs MedPUFA					-0.34 (-1.36,0.67)	⊕◯◯◯Very low
Top vs Pla	***-0.31 (-0.46,-0.17)**	⊕⊕⊕◯Medium	-0.47 (0.64)	⊕⊕◯◯Low	***-0.34 (-0.56,-0.13)**	⊕⊕⊕⊕High
Ami vs Max					-0.04 (-0.89,0.82)	⊕⊕◯◯Low
Ami vs Lam					-0.09 (-0.88,0.71)	⊕⊕◯◯Low
Ami vs MedPUFA					-0.33 (-1.43,0.77)	⊕⊕◯◯Low
Ami vs Pla	-0.28 (-0.63,0.07)	⊕⊕◯◯Low	-0.59 (0.68)	⊕⊕◯◯Low	-0.33 (-0.80,0.14)	⊕⊕⊕◯Medium
Max vs Lam					-0.05 (-1.02,0.92)	⊕⊕◯◯Low
Max vs MedPUFA					-0.30 (-1.52,0.93)	⊕◯◯◯Very low
Max vs Pla	-0.30 (-0.60,0.01)	⊕⊕◯◯Low			-0.30 (-1.01,0.42)	⊕⊕◯◯Low
Lam vs MedPUFA					-0.24 (-1.43,0.95)	⊕◯◯◯Very low
Lam vs Pla	***-0.45 (-0.82,-0.08)**	⊕⊕⊕◯Medium	0.44 (0.69)	⊕⊕◯◯Low	-0.24 (-0.90,0.41)	⊕⊕⊕◯Medium
MedPUFA vs Pla	0.00 (-0.76,0.76)	⊕⊕◯◯Low			0.00 (-0.99,0.99)	⊕⊕◯◯Low

Acceptability in aspect of drop-out rate

	Direct evidence		Indirect evidence		Network meta-analysis	
Comparisons	Odds ratio (95% CI)	The final rating of direct evidence	Co-efficiency (Standard error)	The final rating of indirect evidence	Odds ratio (95% CI)	Overall quality of evidence
AmLowPUFA vs TPr					0.64 (0.12,3.43)	⊕◯◯◯Very low
AmLowPUFA vs Pro					0.57 (0.11,2.81)	⊕◯◯◯Very low
AmLowPUFA vs ToN					0.53 (0.06,4.66)	⊕◯◯◯Very low
AmLowPUFA vs Ami	0.44 (0.10,1.97)	⊕◯◯◯Very low	0.05 (1811.82)	⊕◯◯◯Very low	0.44 (0.10,2.02)	⊕⊕⊕◯Medium
AmLowPUFA vs Pla					0.43 (0.09,2.02)	⊕◯◯◯Very low

AmLowPUFA vs Nor					0.44 (0.04,4.66)	⊕◯◯◯Very low
AmLowPUFA vs Max					0.42 (0.07,2.42)	⊕◯◯◯Very low
AmLowPUFA vs Lam					0.41 (0.06,3.01)	⊕◯◯◯Very low
AmLowPUFA vs HighPUFA					0.43 (0.02,11.10)	⊕◯◯◯Very low
AmLowPUFA vs Top					0.39 (0.08,1.82)	⊕⊕◯◯Low
AmLowPUFA vs Cyc					0.36 (0.06,2.08)	⊕◯◯◯Very low
AmLowPUFA vs Can					0.24 (0.03,1.66)	⊕⊕◯◯Low
AmLowPUFA vs Ven					0.18 (0.02,1.75)	⊕⊕◯◯Low
AmLowPUFA vs Val					0.26 (0.05,1.29)	⊕⊕◯◯Low
AmLowPUFA vs VaLowPUFA					0.07 (0.00,2.90)	⊕◯◯◯Very low
TPr vs Pro					0.89 (0.41,1.93)	⊕⊕◯◯Low
TPr vs ToN					0.82 (0.15,4.36)	⊕◯◯◯Very low
TPr vs Ami					0.69 (0.34,1.42)	⊕⊕◯◯Low
TPr vs Pla					0.67 (0.34,1.32)	⊕⊕◯◯Low
TPr vs Nor					0.69 (0.10,4.57)	⊕◯◯◯Very low
TPr vs Max					0.65 (0.22,1.90)	⊕⊕◯◯Low
TPr vs Lam					0.64 (0.15,2.65)	⊕◯◯◯Very low
TPr vs HighPUFA					0.67 (0.04,12.66)	⊕◯◯◯Very low
TPr vs Top	0.60 (0.34,1.09)	⊕⊕◯◯Low	0.23 (1249.85)	⊕◯◯◯Very low	0.60 (0.32,1.15)	⊕⊕⊕◯Medium
TPr vs Cyc					0.57 (0.20,1.61)	⊕⊕◯◯Low
TPr vs Can					0.37 (0.10,1.43)	⊕⊕◯◯Low
TPr vs Ven					0.28 (0.05,1.69)	⊕⊕◯◯Low
TPr vs Val					***0.40 (0.18,0.90)**	⊕⊕⊕◯Medium
TPr vs VaLowPUFA					0.11 (0.00,3.45)	⊕◯◯◯Very low
Pro vs ToN					0.93 (0.19,4.58)	⊕◯◯◯Very low
Pro vs Ami					0.78 (0.47,1.30)	⊕⊕◯◯Low
Pro vs Pla	0.98 (0.63,1.51)	⊕⊕◯◯Low	-1.13 (0.37)	⊕⊕◯◯Low	0.76 (0.49,1.16)	⊕⊕⊕◯Medium
Pro vs Nor					0.78 (0.13,4.84)	⊕◯◯◯Very low
Pro vs Max					0.73 (0.29,1.88)	⊕⊕◯◯Low

Pro vs Lam					0.72 (0.19,2.73)	⊕◯◯◯Very low
Pro vs HighPUFA					0.76 (0.04,13.63)	⊕◯◯◯Very low
Pro vs Top	***0.52 (0.34,0.81)**	⊕⊕⊕◯Medium	-0.19 (0.32)	⊕⊕◯◯Low	0.68 (0.44,1.05)	⊕⊕⊕◯Medium
Pro vs Cyc	0.74 (0.32,1.68)	⊕◯◯◯Very low	-1.13 (1.02)	⊕⊕◯◯Low	0.64 (0.29,1.40)	⊕⊕⊕◯Medium
Pro vs Can	0.58 (0.13,2.53)	⊕◯◯◯Very low	1.39 (1.04)	⊕⊕◯◯Low	0.42 (0.13,1.36)	⊕⊕⊕◯Medium
Pro vs Ven					0.32 (0.06,1.77)	⊕⊕◯◯Low
Pro vs Val	0.23 (0.02,2.16)	⊕◯◯◯Very low	-0.73 (0.32)	⊕⊕◯◯Low	***0.46 (0.25,0.82)**	⊕⊕⊕⊕High
Pro vs VaLowPUFA					0.13 (0.00,3.73)	⊕◯◯◯Very low
ToN vs Ami					0.85 (0.18,4.07)	⊕◯◯◯Very low
ToN vs Pla					0.82 (0.17,3.86)	⊕◯◯◯Very low
ToN vs Nor	0.84 (0.19,3.79)	⊕◯◯◯Very low			0.84 (0.18,3.88)	⊕⊕◯◯Low
ToN vs Max					0.79 (0.14,4.62)	⊕◯◯◯Very low
ToN vs Lam					0.78 (0.11,5.72)	⊕◯◯◯Very low
ToN vs HighPUFA					0.82 (0.03,21.16)	⊕◯◯◯Very low
ToN vs Top	0.74 (0.16,3.36)	⊕◯◯◯Very low	0.08 (1779.72)	⊕◯◯◯Very low	0.74 (0.16,3.44)	⊕⊕⊕◯Medium
ToN vs Cyc					0.69 (0.12,3.96)	⊕◯◯◯Very low
ToN vs Can					0.46 (0.07,3.16)	⊕◯◯◯Very low
ToN vs Ven					0.34 (0.04,3.33)	⊕◯◯◯Very low
ToN vs Val					0.49 (0.10,2.46)	⊕◯◯◯Very low
ToN vs VaLowPUFA					0.14 (0.00,5.53)	⊕◯◯◯Very low
Ami vs Pla	0.87 (0.62,1.21)	⊕⊕◯◯Low	0.14 (0.27)	⊕⊕◯◯Low	0.97 (0.71,1.32)	⊕⊕⊕◯Medium
Ami vs Nor					1.00 (0.16,6.04)	⊕◯◯◯Very low
Ami vs Max					0.94 (0.39,2.28)	⊕◯◯◯Very low
Ami vs Lam					0.92 (0.25,3.38)	⊕◯◯◯Very low
Ami vs HighPUFA					0.97 (0.05,17.16)	⊕◯◯◯Very low
Ami vs Top	0.98 (0.67,1.43)	⊕◯◯◯Very low	0.25 (0.23)	⊕⊕◯◯Low	0.87 (0.64,1.19)	⊕⊕⊕◯Medium
Ami vs Cyc					0.82 (0.35,1.93)	⊕⊕◯◯Low
Ami vs Can					0.54 (0.16,1.80)	⊕⊕◯◯Low
Ami vs Ven					0.41 (0.07,2.20)	⊕◯◯◯Very low
Ami vs Val					***0.58 (0.34,0.99)**	⊕⊕⊕◯Medium

Ami vs VaLowPUFA					0.17 (0.01,4.71)	⊕◯◯◯Very low
Pla vs Nor					1.03 (0.17,6.11)	⊕◯◯◯Very low
Pla vs Max	0.97 (0.44,2.13)	⊕◯◯◯Very low			0.97 (0.42,2.23)	⊕⊕◯◯Low
Pla vs Lam	1.00 (0.24,4.21)	⊕◯◯◯Very low	0.22 (1.31)	⊕⊕◯◯Low	0.95 (0.27,3.38)	⊕⊕⊕◯Medium
Pla vs HighPUFA	1.00 (0.06,17.18)	⊕◯◯◯Very low			1.00 (0.06,17.41)	⊕◯◯◯Very low
Pla vs Top	0.89 (0.73,1.08)	⊕⊕◯◯Low	-0.06 (0.32)	⊕⊕◯◯Low	0.90 (0.75,1.08)	⊕⊕⊕◯Medium
Pla vs Cyc	0.69 (0.27,1.75)	⊕⊕◯◯Low	-0.46 (0.95)	⊕⊕◯◯Low	0.85 (0.38,1.89)	⊕⊕⊕◯Medium
Pla vs Can	0.41 (0.10,1.64)	⊕⊕◯◯Low	-0.49 (1.53)	⊕⊕◯◯Low	0.56 (0.17,1.79)	⊕⊕⊕◯Medium
Pla vs Ven	0.42 (0.08,2.16)	⊕◯◯◯Very low			0.42 (0.08,2.21)	⊕◯◯◯Very low
Pla vs Val	***0.62 (0.41,0.94)**	⊕◯◯◯Very low	1.22 (1.17)	⊕⊕◯◯Low	***0.60 (0.39,0.92)**	⊕⊕⊕⊕High
Pla vs VaLowPUFA					0.17 (0.01,4.79)	⊕◯◯◯Very low
Nor vs Max					0.94 (0.13,6.76)	⊕◯◯◯Very low
Nor vs Lam					0.92 (0.10,8.19)	⊕◯◯◯Very low
Nor vs HighPUFA					0.97 (0.03,28.27)	⊕◯◯◯Very low
Nor vs Top	0.87 (0.15,5.05)	⊕◯◯◯Very low	-0.05 (1793.18)	⊕◯◯◯Very low	0.88 (0.15,5.16)	⊕⊕⊕◯Medium
Nor vs Cyc					0.82 (0.12,5.81)	⊕◯◯◯Very low
Nor vs Can					0.54 (0.06,4.55)	⊕◯◯◯Very low
Nor vs Ven					0.41 (0.04,4.67)	⊕◯◯◯Very low
Nor vs Val					0.59 (0.09,3.67)	⊕◯◯◯Very low
Nor vs VaLowPUFA					0.17 (0.00,7.30)	⊕◯◯◯Very low
Max vs Lam					0.98 (0.21,4.48)	⊕◯◯◯Very low
Max vs HighPUFA					1.03 (0.05,20.28)	⊕◯◯◯Very low
Max vs Top					0.93 (0.40,2.18)	⊕◯◯◯Very low
Max vs Cyc					0.87 (0.27,2.79)	⊕◯◯◯Very low
Max vs Can					0.58 (0.14,2.41)	⊕◯◯◯Very low
Max vs Ven					0.43 (0.07,2.78)	⊕◯◯◯Very low
Max vs Val					0.62 (0.24,1.59)	⊕⊕◯◯Low
Max vs VaLowPUFA					0.18 (0.01,5.49)	⊕◯◯◯Very low
Lam vs HighPUFA					1.05 (0.05,24.05)	⊕◯◯◯Very low
Lam vs Top	1.00 (0.24,4.21)	⊕◯◯◯Very low	0.22 (1.31)	⊕⊕◯◯Low	0.95 (0.27,3.38)	⊕⊕⊕◯Medium

Lam vs Cyc					0.89 (0.20,4.00)	⊕◯◯◯Very low
Lam vs Can					0.59 (0.10,3.29)	⊕◯◯◯Very low
Lam vs Ven					0.44 (0.05,3.58)	⊕◯◯◯Very low
Lam vs Val					0.63 (0.17,2.42)	⊕◯◯◯Very low
Lam vs VaLowPUFA					0.18 (0.01,6.39)	⊕◯◯◯Very low
HighPUFA vs Top					0.90 (0.05,15.73)	⊕◯◯◯Very low
HighPUFA vs Cyc					0.85 (0.04,16.47)	⊕◯◯◯Very low
HighPUFA vs Can					0.56 (0.03,12.18)	⊕◯◯◯Very low
HighPUFA vs Ven					0.42 (0.02,11.41)	⊕◯◯◯Very low
HighPUFA vs Val					0.60 (0.03,10.80)	⊕◯◯◯Very low
HighPUFA vs VaLowPUFA					0.17 (0.00,13.78)	⊕◯◯◯Very low
Top vs Cyc					0.94 (0.42,2.12)	⊕◯◯◯Very low
Top vs Can					0.62 (0.19,2.00)	⊕◯◯◯Very low
Top vs Ven					0.47 (0.09,2.48)	⊕◯◯◯Very low
Top vs Val					0.67 (0.42,1.06)	⊕⊕◯◯Low
Top vs VaLowPUFA					0.19 (0.01,5.36)	⊕◯◯◯Very low
Cyc vs Can					0.66 (0.17,2.61)	⊕◯◯◯Very low
Cyc vs Ven					0.49 (0.08,3.14)	⊕◯◯◯Very low
Cyc vs Val					0.71 (0.29,1.76)	⊕⊕◯◯Low
Cyc vs VaLowPUFA					0.20 (0.01,6.22)	⊕◯◯◯Very low
Can vs Ven					0.75 (0.10,5.73)	⊕◯◯◯Very low
Can vs Val					1.08 (0.31,3.72)	⊕◯◯◯Very low
Can vs VaLowPUFA					0.31 (0.01,10.48)	⊕◯◯◯Very low
Ven vs Val					1.44 (0.26,8.01)	⊕◯◯◯Very low
Ven vs VaLowPUFA					0.41 (0.01,16.96)	⊕◯◯◯Very low
Val vs VaLowPUFA	0.28 (0.01,7.67)	⊕◯◯◯Very low	1.30 (2409.10)	⊕◯◯◯Very low	0.28 (0.01,7.75)	⊕⊕⊕◯Medium

We followed Cochrane Handbook for GRADE ratings in BMJ(40) and one important network meta-analysis in Lancet(41) for quality assessment

Reference list of supplement materials:

1. Page MJ, McKenzie JE, Bossuyt PM, Boutron I, Hoffmann TC, Mulrow CD et al. The PRISMA 2020 statement: an updated guideline for reporting systematic reviews. Bmj 2021;372:n71.
2. Elkind AH, Satin LZ, Nila A, Keywood C. Frovatriptan use in migraineurs with or at high risk of coronary artery disease. Headache 2004;44(5):403-410.
3. Ryan R, Geraud G, Goldstein J, Cady R, Keywood C. Clinical efficacy of frovatriptan: placebo-controlled studies. Headache 2002;42 Suppl 2:S84-92.
4. Rapoport A, Ryan R, Goldstein J, Keywood C. Dose range-finding studies with frovatriptan in the acute treatment of migraine. Headache 2002;42 Suppl 2:S74-83.
5. Goldstein J, Keywood C, Study G. Frovatriptan for the acute treatment of migraine: a dose-finding study. Headache 2002;42(1):41-48.
6. Cady R, Elkind A, Goldstein J, Keywood C. Randomized, placebo-controlled comparison of early use of frovatriptan in a migraine attack versus dosing after the headache has become moderate or severe. Curr Med Res Opin 2004;20(9):1465-1472.
7. Ramsden CE, Faurot KR, Zamora D, Suchindran CM, MacIntosh BA, Gaylord S et al. Targeted alteration of dietary n-3 and n-6 fatty acids for the treatment of chronic headaches: a randomized trial. Pain 2013;154(11):2441-2451.
8. MacIntosh BA, Ramsden CE, Honvoh G, Faurot KR, Palsson OS, Johnston AD et al. Methodology for altering omega-3 EPA+DHA and omega-6 linoleic acid as controlled variables in a dietary trial. Clin Nutr 2021;40(6):3859-3867.

9. Abdolahi M, Tafakhori A, Togha M, Okhovat AA, Siassi F, Eshraghian MR et al. The synergistic effects of omega-3 fatty acids and nano-curcumin supplementation on tumor necrosis factor (TNF)-alpha gene expression and serum level in migraine patients. Immunogenetics 2017;69(6):371-378.
10. Silberstein S, Lipton R, Dodick D, Freitag F, Mathew N, Brandes J et al. Topiramate treatment of chronic migraine: a randomized, placebo-controlled trial of quality of life and other efficacy measures. Headache 2009;49(8):1153-1162.
11. Dodick DW, Silberstein S, Saper J, Freitag FG, Cady RK, Rapoport AM et al. The impact of topiramate on health-related quality of life indicators in chronic migraine. Headache 2007;47(10):1398-1408.
12. Ziegler DK, Hurwitz A, Preskorn S, Hassanein R, Seim J. Propranolol and amitriptyline in prophylaxis of migraine. Pharmacokinetic and therapeutic effects. Arch Neurol 1993;50(8):825-830.
13. Latsko M, Silberstein S, Rosen N. Frovatriptan as preemptive treatment for fasting-induced migraine. Headache 2011;51(3):369-374.
14. Coffee AL, Sulak PJ, Hill AJ, Hansen DJ, Kuehl TJ, Clark JW. Extended cycle combined oral contraceptives and prophylactic frovatriptan during the hormone-free interval in women with menstrual-related migraines. Journal of women's health (2002) 2014;23(4):310-317.
15. Brandes JL, Poole A, Kallela M, Schreiber CP, MacGregor EA, Silberstein SD et al. Short-term frovatriptan for the prevention of difficult-to-treat menstrual migraine attacks. Cephalalgia 2009;29(11):1133-1148.
16. Silberstein SD, Berner T, Tobin J, Xiang Q, Campbell JC. Scheduled short-term prevention with frovatriptan for migraine occurring exclusively in association with menstruation. Headache 2009;49(9):1283-1297.
17. Wade A, Pawsey S, Whale H, Boyce M, Warrington S. Pharmacokinetics of two 6-day frovatriptan dosing regimens used for the short-term prevention of menstrual migraine: A phase I, randomized, double-blind, placebo-controlled, two-period crossover, single-centre study in healthy female volunteers. Clinical drug investigation 2009;29(5):325-337.
18. Silberstein SD, Elkind AH, Schreiber C, Keywood C. A randomized trial of frovatriptan for the intermittent prevention of menstrual migraine. Neurology 2004;63(2):261-269.
19. Honarvar NM, Soveid N, Abdolahi M, Djalali M, Hatami M, Karzar NH. Anti-Neuroinflammatory Properties of n-3 Fatty Acids and Nano-Curcumin on Migraine Patients from Cellular to Clinical Insight: A Randomized, Double-Blind and Placebo-Controlled Trial. Endocr Metab

Immune Disord Drug Targets 2021;21(2):365-373.
20. Abdolahi M, Jafarieh A, Sarraf P, Sedighiyan M, Yousefi A, Tafakhori A et al. The Neuromodulatory Effects of omega-3 Fatty Acids and Nano-Curcumin on the COX-2/ iNOS Network in Migraines: A Clinical Trial Study from Gene Expression to Clinical Symptoms. Endocr Metab Immune Disord Drug Targets 2019;19(6):874-884.
21. Abdolahi M, Sarraf P, Javanbakht MH, Honarvar NM, Hatami M, Soveyd N et al. A Novel Combination of omega-3 Fatty Acids and Nano-Curcumin Modulates Interleukin-6 Gene Expression and High Sensitivity C-reactive Protein Serum Levels in Patients with Migraine: A Randomized Clinical Trial Study. CNS Neurol Disord Drug Targets 2018;17(6):430-438.
22. Soveyd N, Abdolahi M, Djalali M, Hatami M, Tafakhori A, Sarraf P et al. The Combined Effects of omega -3 Fatty Acids and Nano-Curcumin Supplementation on Intercellular Adhesion Molecule-1 (ICAM-1) Gene Expression and Serum Levels in Migraine Patients. CNS Neurol Disord Drug Targets 2018;16(10):1120-1126.
23. Pfaffenrath V, Diener HC, Isler H, Meyer C, Scholz E, Taneri Z et al. [Effectiveness and tolerance of amitriptyline oxide in chronic tension headache--a multicenter double-blind study versus amitriptyline versus placebo]. Nervenarzt 1993;64(2):114-120.
24. Ferrara LA, Pacioni D, Di Fronzo V, Russo BF, Speranza E, Carlino V et al. Low-lipid diet reduces frequency and severity of acute migraine attacks. Nutr Metab Cardiovasc Dis 2015;25(4):370-375.
25. Maghsoumi-Norouzabad L, Mansoori A, Abed R, Shishehbor F. Effects of omega-3 fatty acids on the frequency, severity, and duration of migraine attacks: A systematic review and meta-analysis of randomized controlled trials. Nutr Neurosci 2018;21(9):614-623.
26. Su KP, Tseng PT, Lin PY, Okubo R, Chen TY, Chen YW et al. Association of Use of Omega-3 Polyunsaturated Fatty Acids With Changes in Severity of Anxiety Symptoms: A Systematic Review and Meta-analysis. JAMA Netw Open 2018;1(5):e182327.
27. Rothrock JF, Adams AM, Lipton RB, Silberstein SD, Jo E, Zhao X et al. FORWARD Study: Evaluating the Comparative Effectiveness of OnabotulinumtoxinA and Topiramate for Headache Prevention in Adults With Chronic Migraine. Headache 2019;59(10):1700-1713.
28. Cady RK, Schreiber CP, Porter JA, Blumenfeld AM, Farmer KU. A multi-center double-blind pilot comparison of onabotulinumtoxinA and topiramate for the prophylactic treatment of chronic migraine. Headache 2011;51(1):21-32.
29. Mathew NT, Jaffri SF. A double-blind comparison of onabotulinumtoxina (BOTOX) and topiramate (TOPAMAX) for the prophylactic

treatment of chronic migraine: a pilot study. Headache 2009;49(10):1466-1478.
30. Blumenfeld AM, Schim JD, Chippendale TJ. Botulinum toxin type A and divalproex sodium for prophylactic treatment of episodic or chronic migraine. Headache 2008;48(2):210-220.
31. Ramsden CE, Zamora D, Faurot KR, MacIntosh B, Horowitz M, Keyes GS et al. Dietary alteration of n-3 and n-6 fatty acids for headache reduction in adults with migraine: randomized controlled trial. Bmj 2021;374:n1448.
32. Tajmirriahi M, Sohelipour M, Basiri K, Shaygannejad V, Ghorbani A, Saadatnia M. The effects of sodium valproate with fish oil supplementation or alone in migraine prevention: A randomized single-blind clinical trial. Iran J Neurol 2012;11(1):21-24.
33. Kelishadi MR, Naeini AA, Khorvash F, Askari G, Heidari Z. The beneficial effect of Alpha-lipoic acid supplementation as a potential adjunct treatment in episodic migraines. Scientific reports 2022;12(1):271.
34. Rezaei Kelishadi M, Alavi Naeini A, Askari G, Khorvash F, Heidari Z. The efficacy of alpha-lipoic acid in improving oxidative, inflammatory, and mood status in women with episodic migraine in a randomised, double-blind, placebo-controlled clinical trial. International journal of clinical practice 2021;75(9):e14455.
35. Magis D, Ambrosini A, Sandor P, Jacquy J, Laloux P, Schoenen J. A randomized double-blind placebo-controlled trial of thioctic acid in migraine prophylaxis. Headache 2007;47(1):52-57.
36. Cavestro C, Bedogni G, Molinari F, Mandrino S, Rota E, Frigeri MC. Alpha-Lipoic Acid Shows Promise to Improve Migraine in Patients with Insulin Resistance: A 6-Month Exploratory Study. J Med Food 2018;21(3):269-273.
37. Rist PM, Buring JE, Cook NR, Manson JE, Kurth T. Effect of Vitamin D and/or Marine n-3 Fatty Acid Supplementation on Changes in Migraine Frequency and Severity. The American journal of medicine 2021;134(6):756-762 e755.
38. Mann JD, Faurot KR, MacIntosh B, Palsson OS, Suchindran CM, Gaylord SA et al. A sixteen-week three-armed, randomized, controlled trial investigating clinical and biochemical effects of targeted alterations in dietary linoleic acid and n-3 EPA+DHA in adults with episodic migraine: Study protocol. Prostaglandins Leukot Essent Fatty Acids 2018;128:41-52.
39. Hershey AD, Powers SW, Coffey CS, Eklund DD, Chamberlin LA, Korbee LL et al. Childhood and Adolescent Migraine Prevention (CHAMP) study: a double-blinded, placebo-controlled, comparative effectiveness study of amitriptyline, topiramate, and placebo in the prevention of

childhood and adolescent migraine. Headache 2013;53(5):799-816.
40. Puhan MA, Schunemann HJ, Murad MH, Li T, Brignardello-Petersen R, Singh JA et al. A GRADE Working Group approach for rating the quality of treatment effect estimates from network meta-analysis. Bmj 2014;349:g5630.
41. Cipriani A, Furukawa TA, Salanti G, Chaimani A, Atkinson LZ, Ogawa Y et al. Comparative efficacy and acceptability of 21 antidepressant drugs for the acute treatment of adults with major depressive disorder: a systematic review and network meta-analysis. Lancet 2018;391(10128):1357-1366.

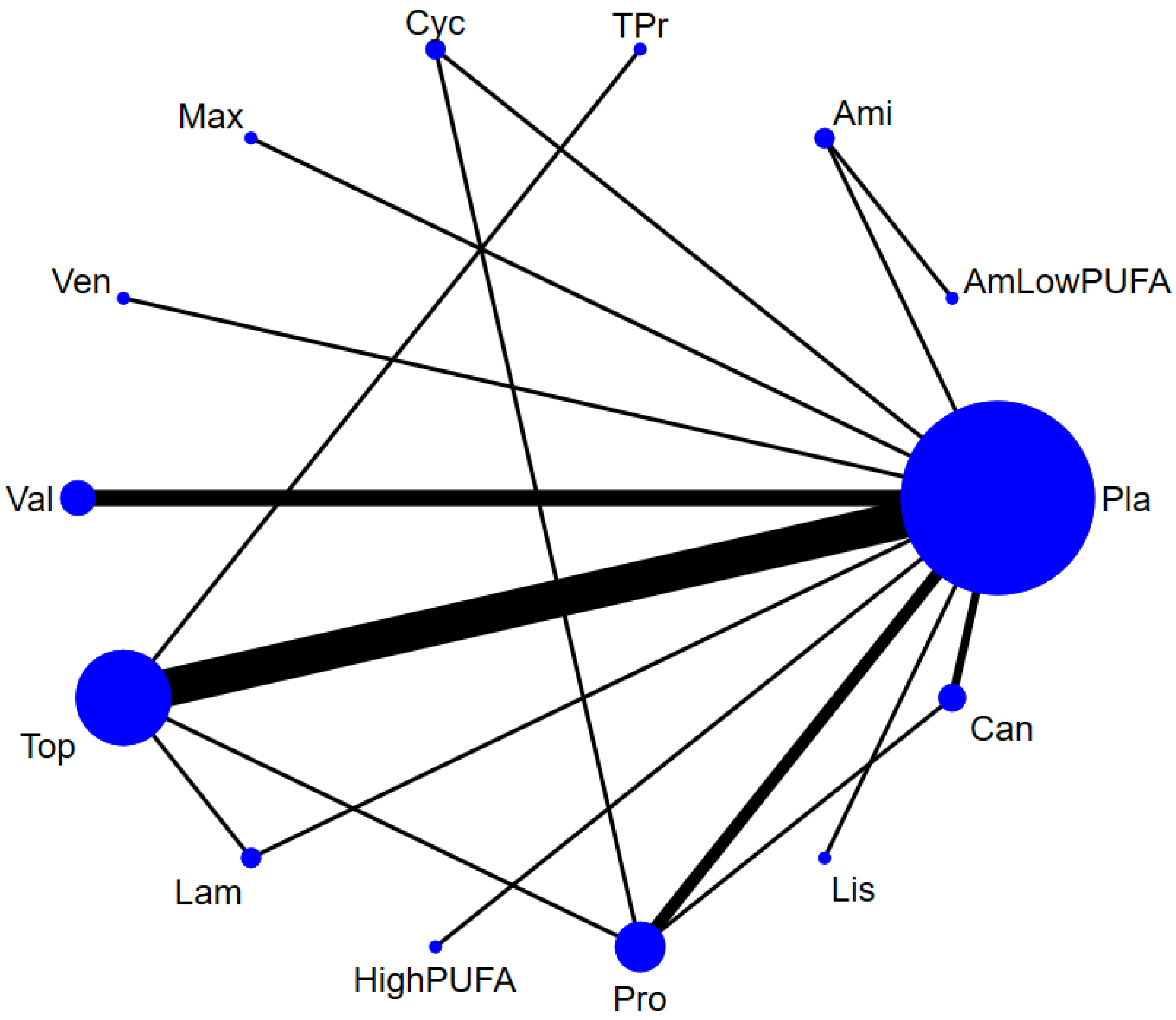

eFigure 1A network structure of primary outcome: migraine frequency-subgroup of adult

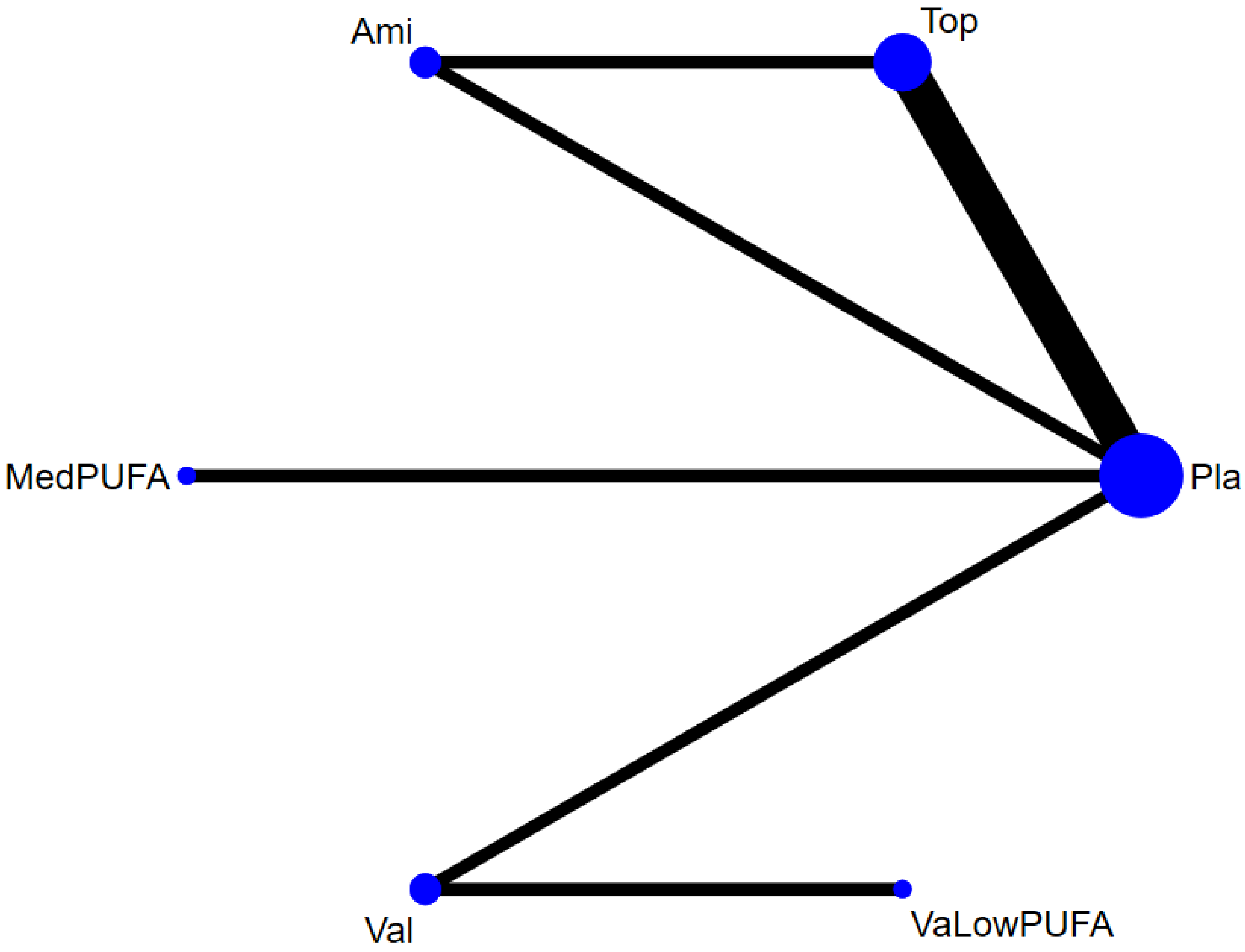

eFigure 1B network structure of primary outcome: migraine frequency-subgroup of child

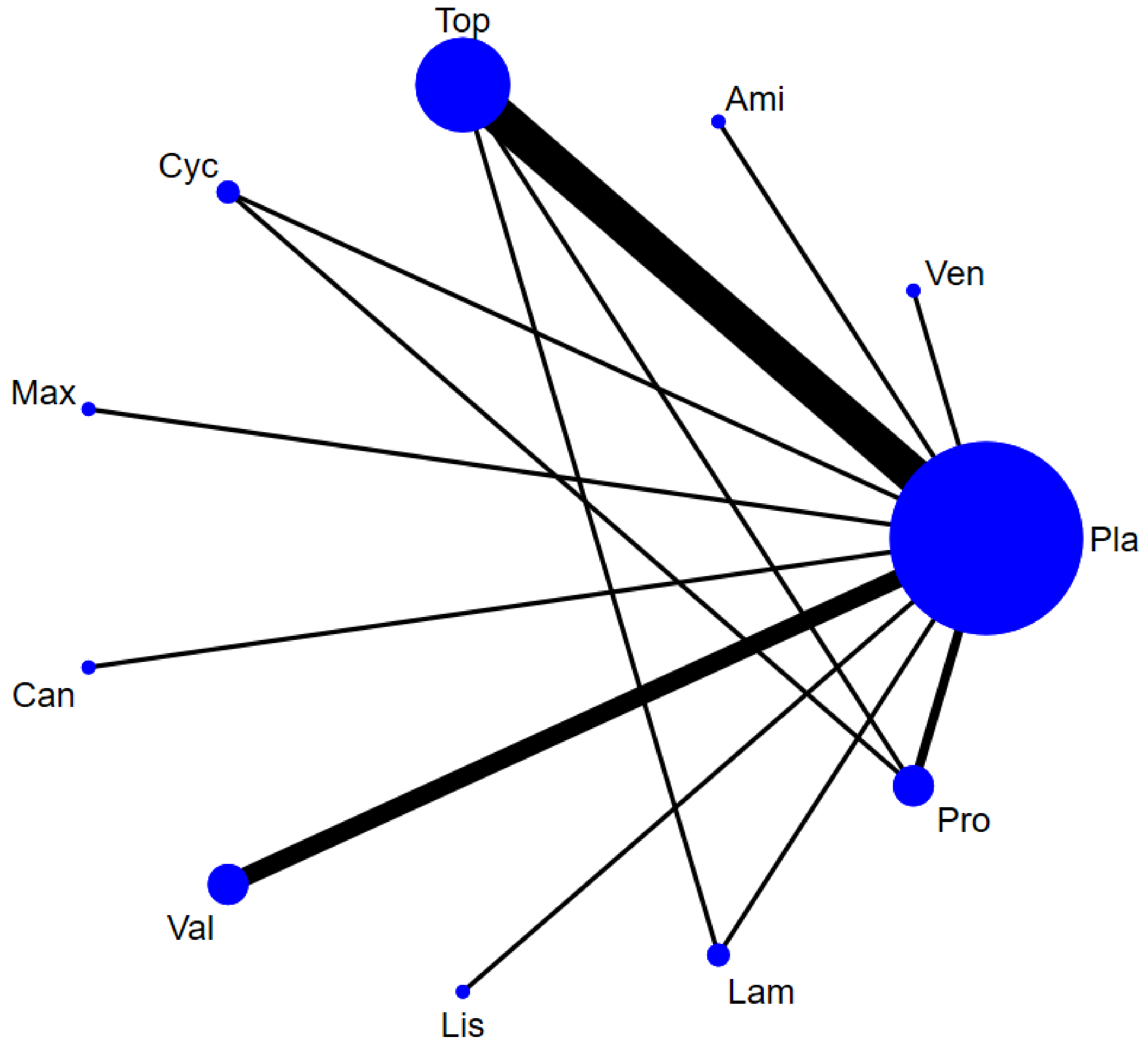

eFigure 1C network structure of primary outcome: migraine frequency-subgroup of episodic migraine

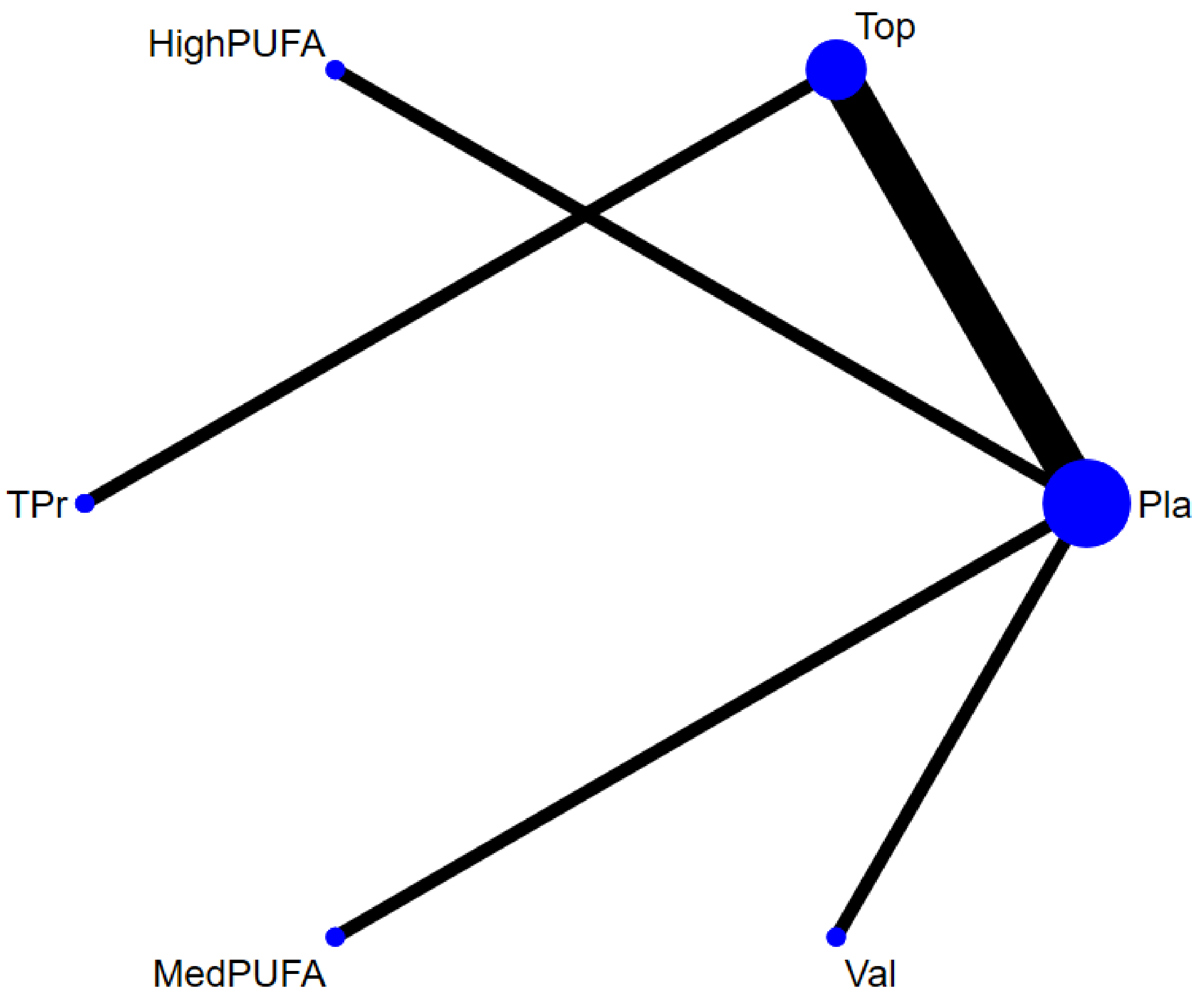

eFigure 1D network structure of primary outcome: migraine frequency-subgroup of chronic migraine

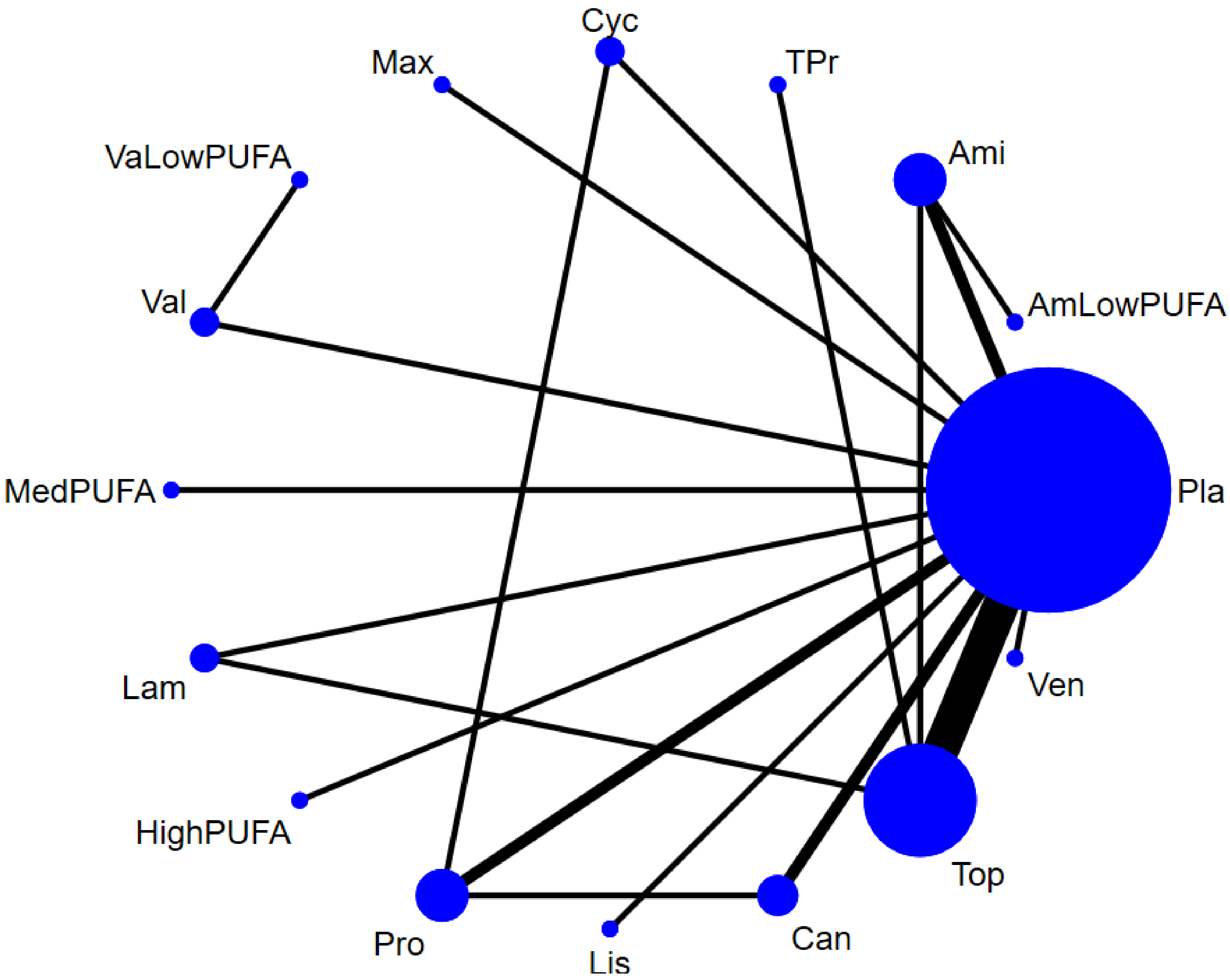

eFigure 1E network structure of primary outcome: migraine frequency-excluding high risk trials

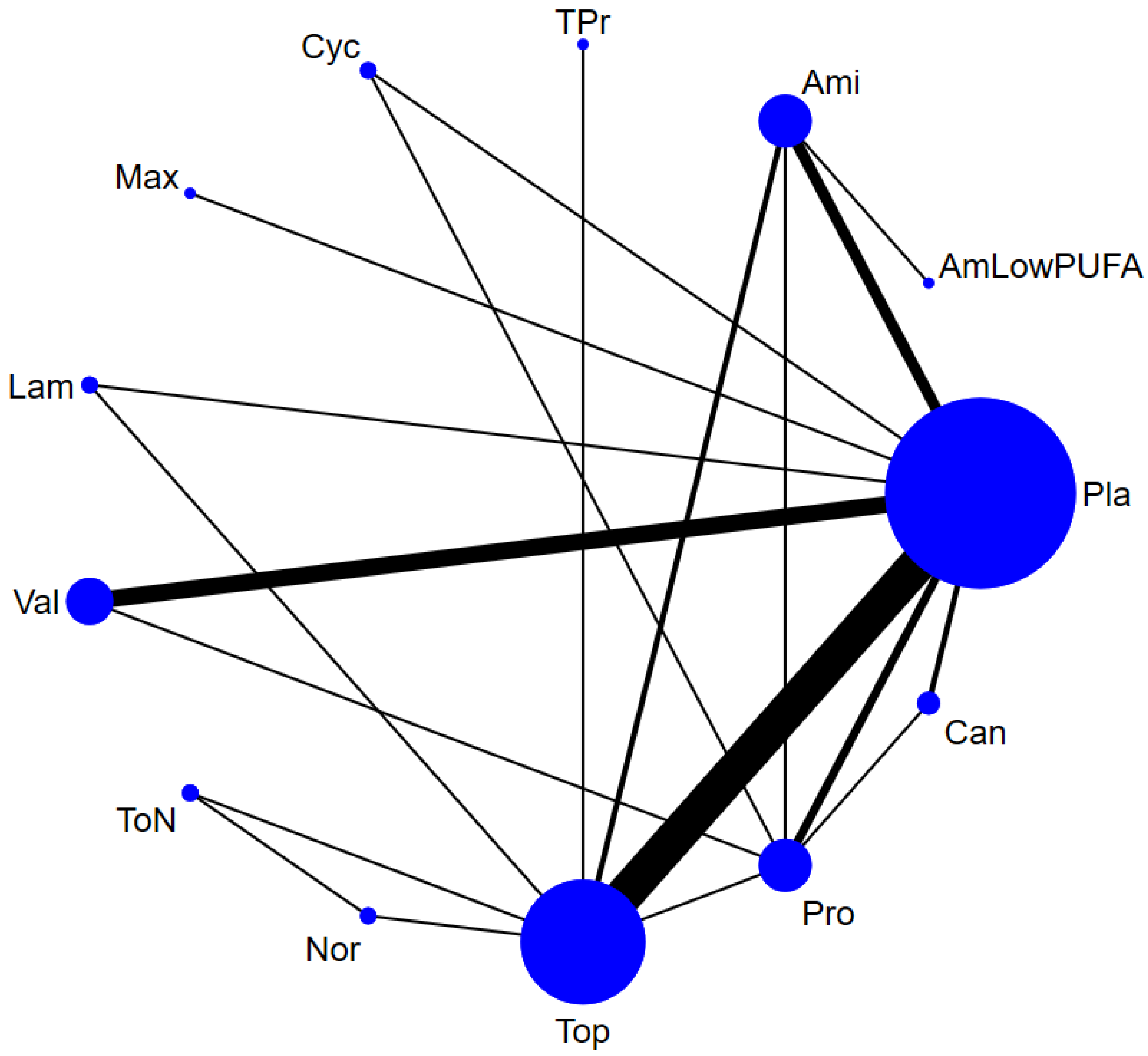

eFigure 1F network structure of secondary outcome: response rate

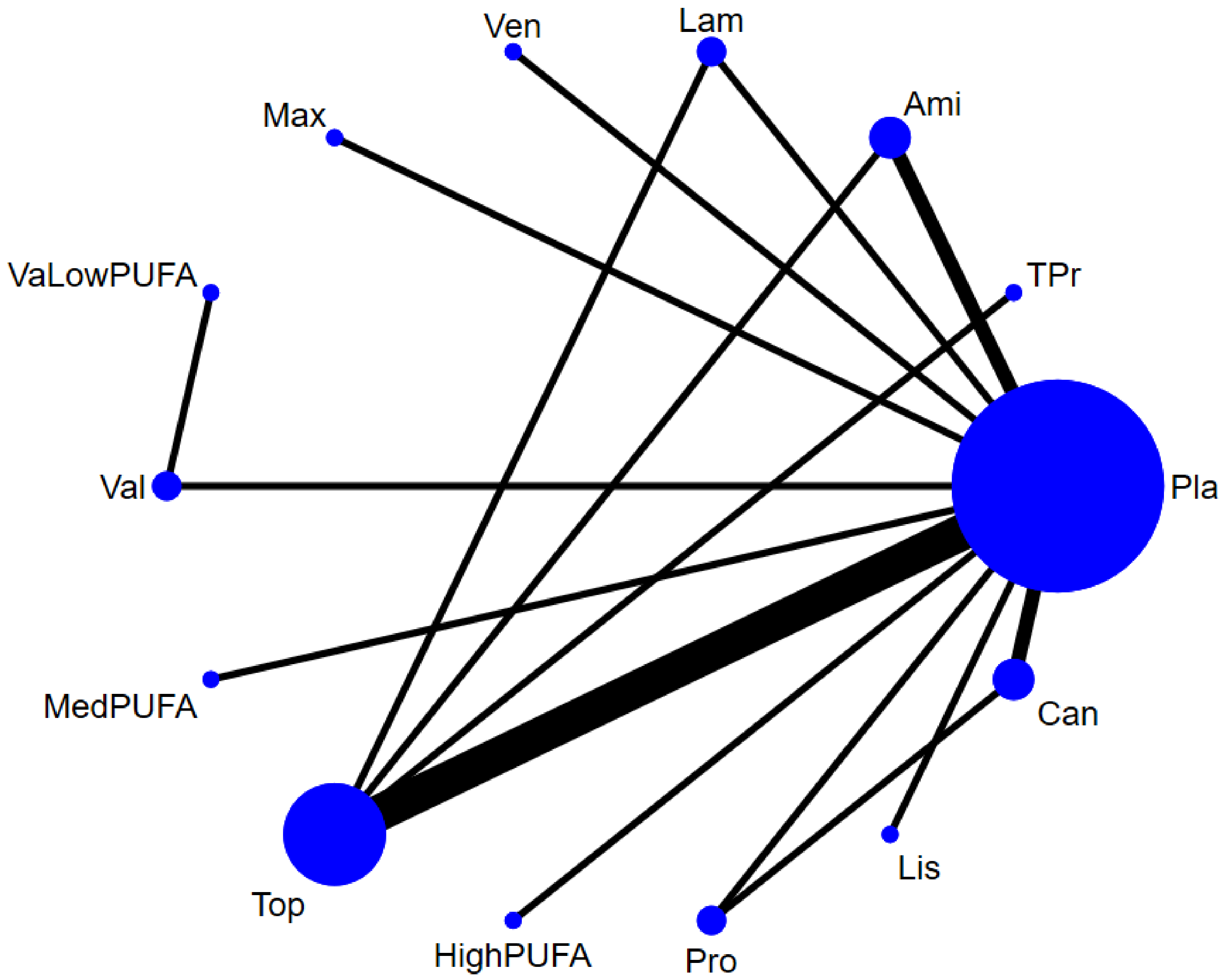

eFigure 1G network structure of secondary outcome: migraine severity

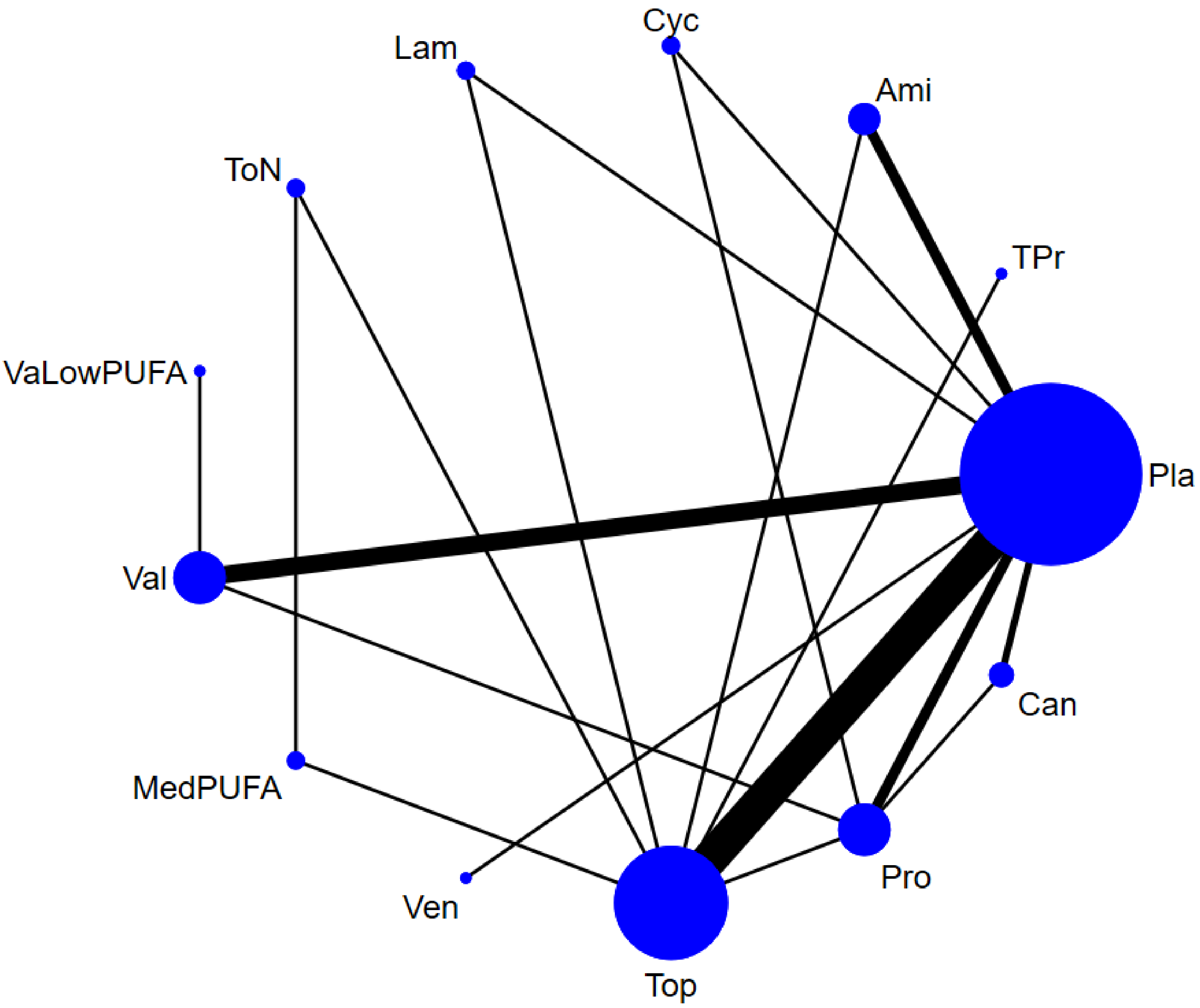

eFigure 1H network structure of safety profile: rate of any adverse event

Figure legend of eFigure 1A-1H

Lines between nodes represent direct comparisons between trials, and circle size is proportional to the size of the population that received each treatment. Line thickness is proportional to the number of trials connected to the network.

Abbreviation: 95%CI: 95% confidence interval; Ami: amitriptyline; AmLowPUFA: low dosage n3PUFA + amitriptyline; AMSTAR: assessing the methodological quality of systematic review; Bot: Botox-A; Can: candesartan; CGRP: calcitonin gene-related peptide; Cyc: cyclandelate; DHA: docosahexaenoic acid; EPA: eicosapentaenoic acid; ES: effect size; HighPUFA: high dosage n3PUFA; Lam: lamotrigine; Lis: lisinopril; Max: Maxepa (omega-3 polyunsaturated fatty acids, EPA/DHA: 180mg/120mg x 6 pills); MedPUFA: medium dosage n3PUFA; Mem: memantine; NAM: network meta-analysis; Nor: nortriptyline; OR: odds ratio; Pla: Placebo; PRISMA: Preferred Reporting Items for Systematic Reviews and Meta-Analyses; Pro: propranolol; PUFA: polyunsaturated fatty acid; RCT: randomized controlled trial; SMD: standardized mean difference; SUCRA: surface under the cumulative ranking curve; ToN: topiramate + nortriptyline; Top: topiramate; TPr: topiramate + propranolol; TVGT: trigeminal nerve-trigeminocervical complex-ventroposteromedial thalamic nucleus; Val: valproate; VaLowPUFA: low dosage n3PUFA + valproate; Ven: venlafaxine

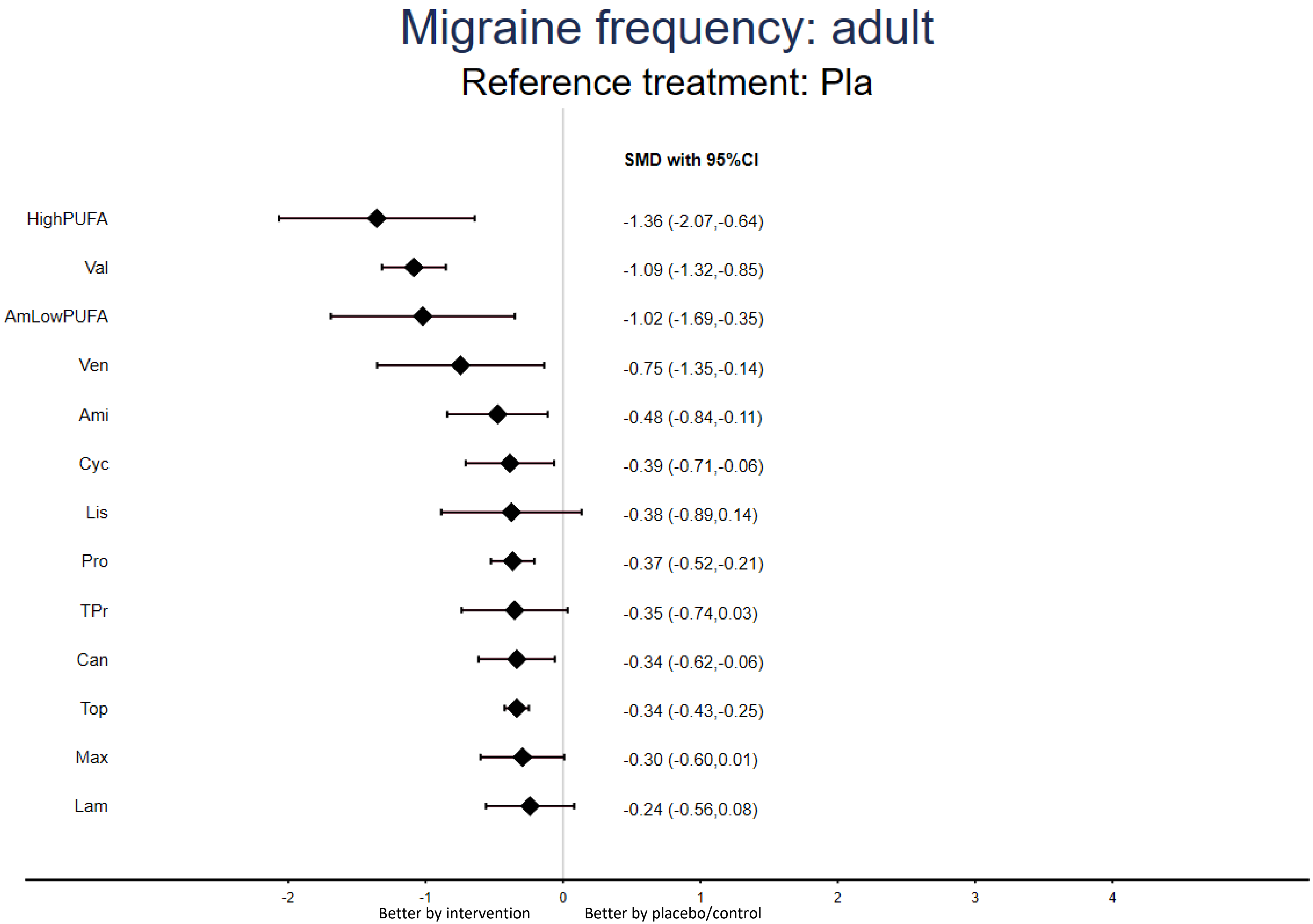

eFigure 2A forest plot of primary outcome: migraine frequency-subgroup of adult

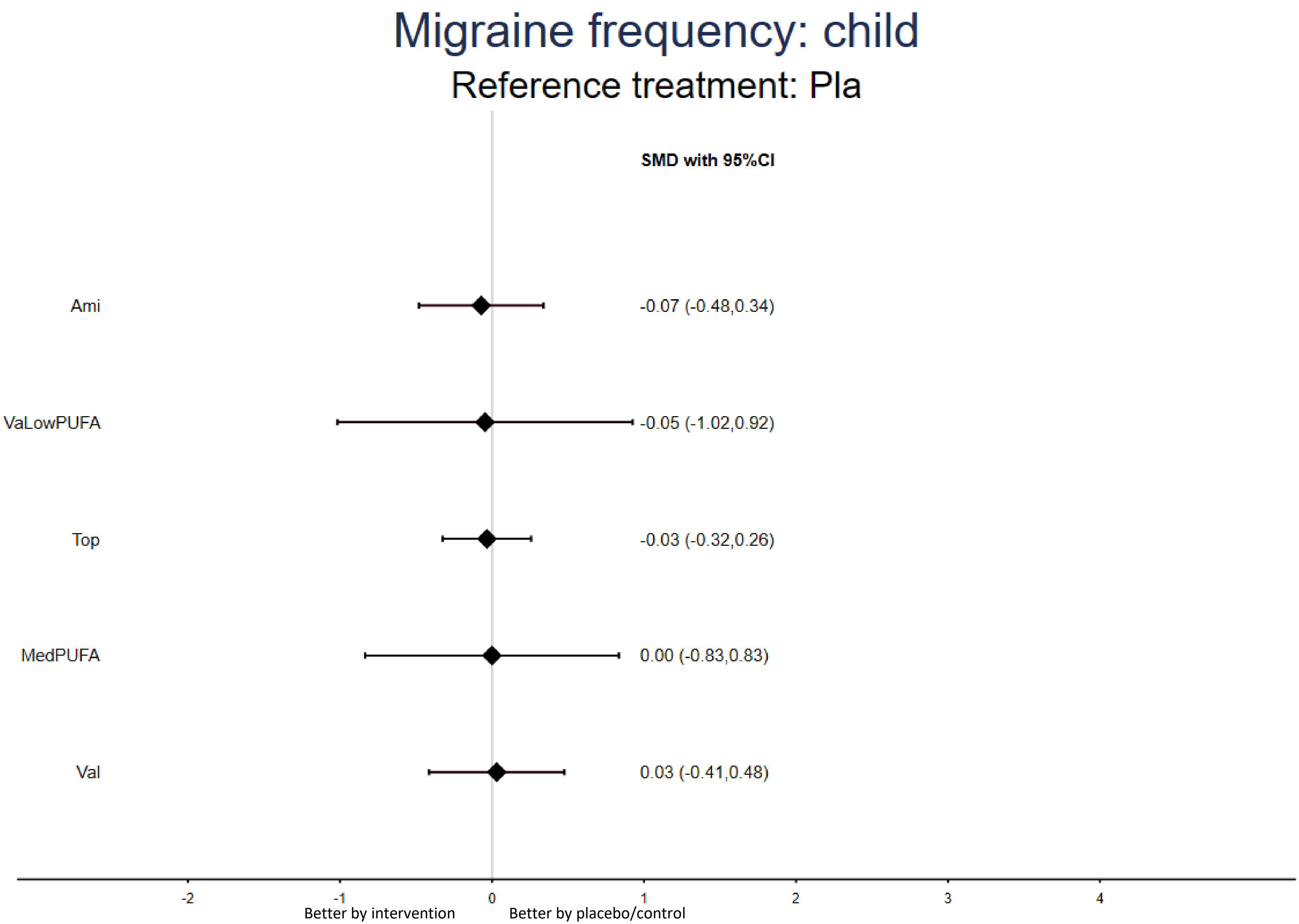

eFigure 2B forest plot of primary outcome: migraine frequency-subgroup of child

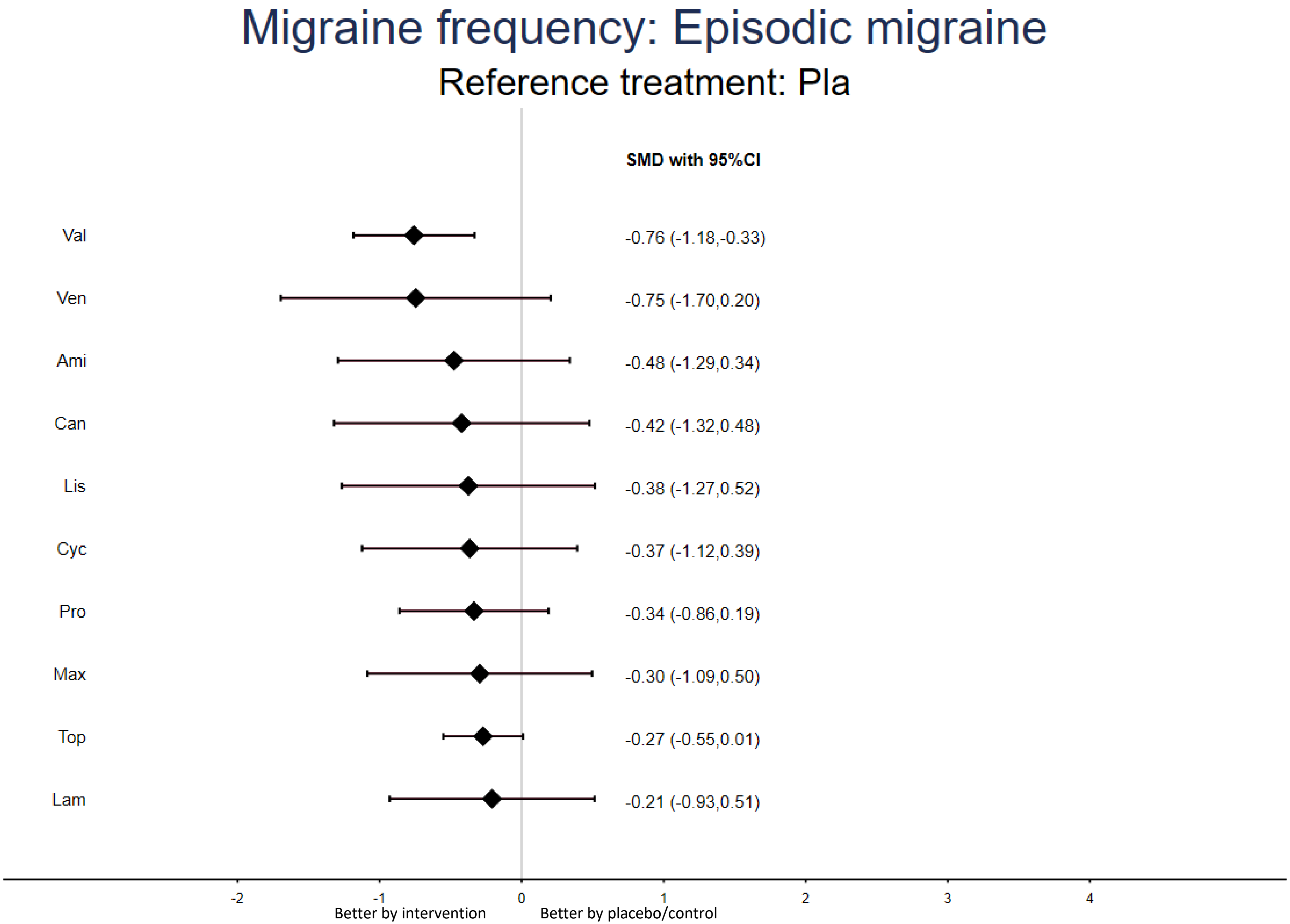

eFigure 2C forest plot of primary outcome: migraine frequency-subgroup of episodic migraine

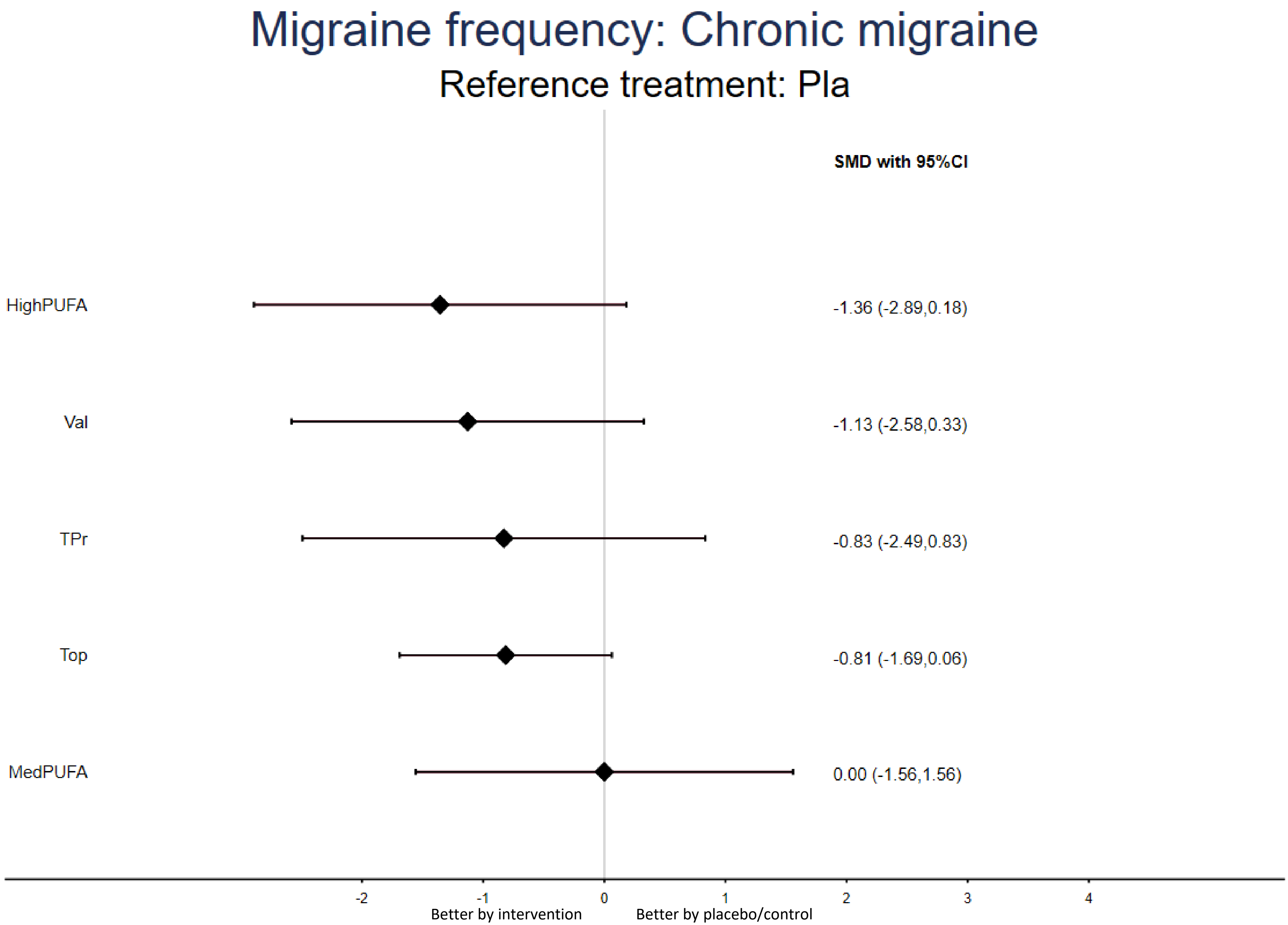

eFigure 2D forest plot of primary outcome: migraine frequency-subgroup of chronic migraine

Migraine frequency: excluding high risk studies

Reference treatment: Pla

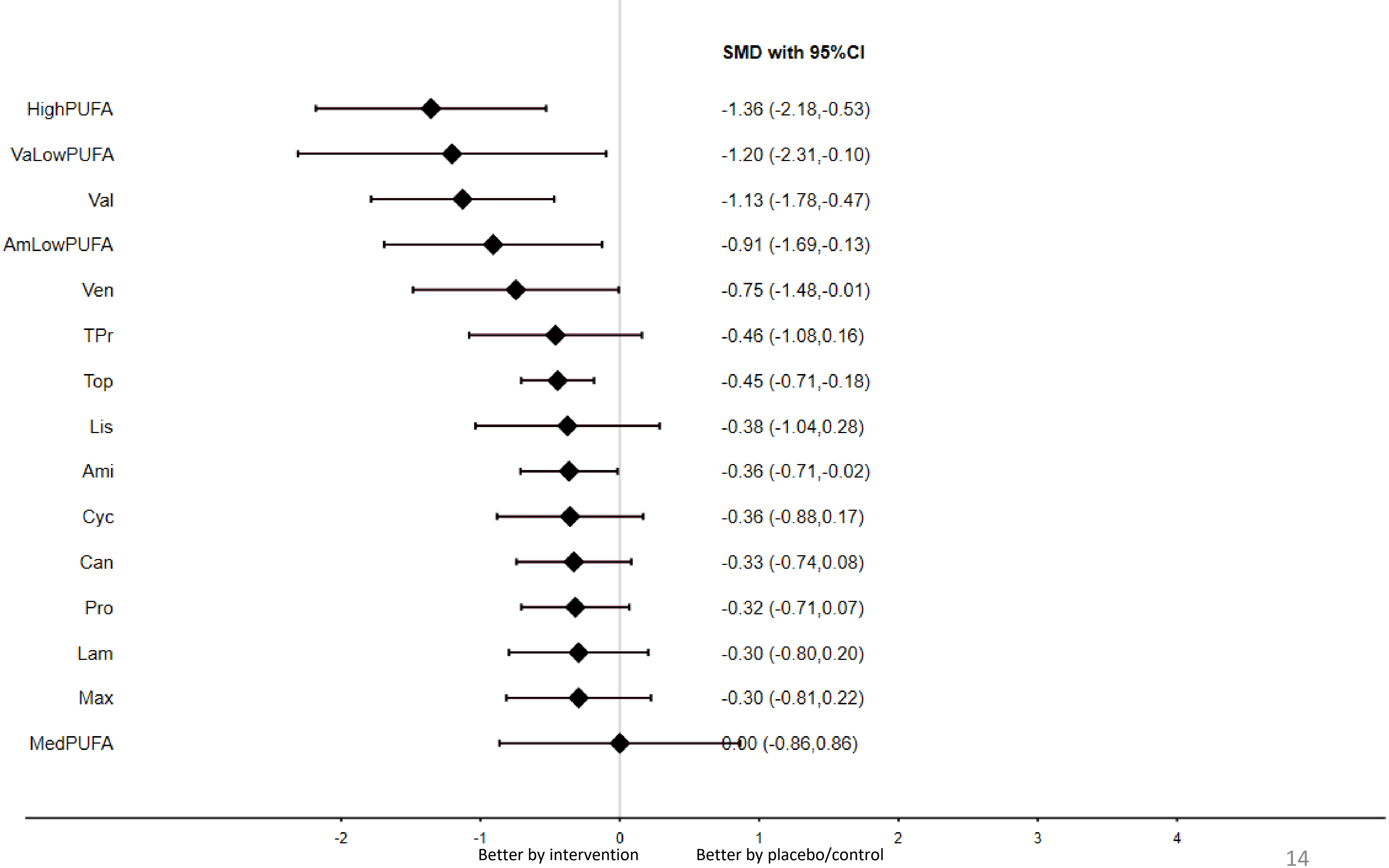

eFigure 2E forest plot of primary outcome: migraine frequency-excluding high risk trials

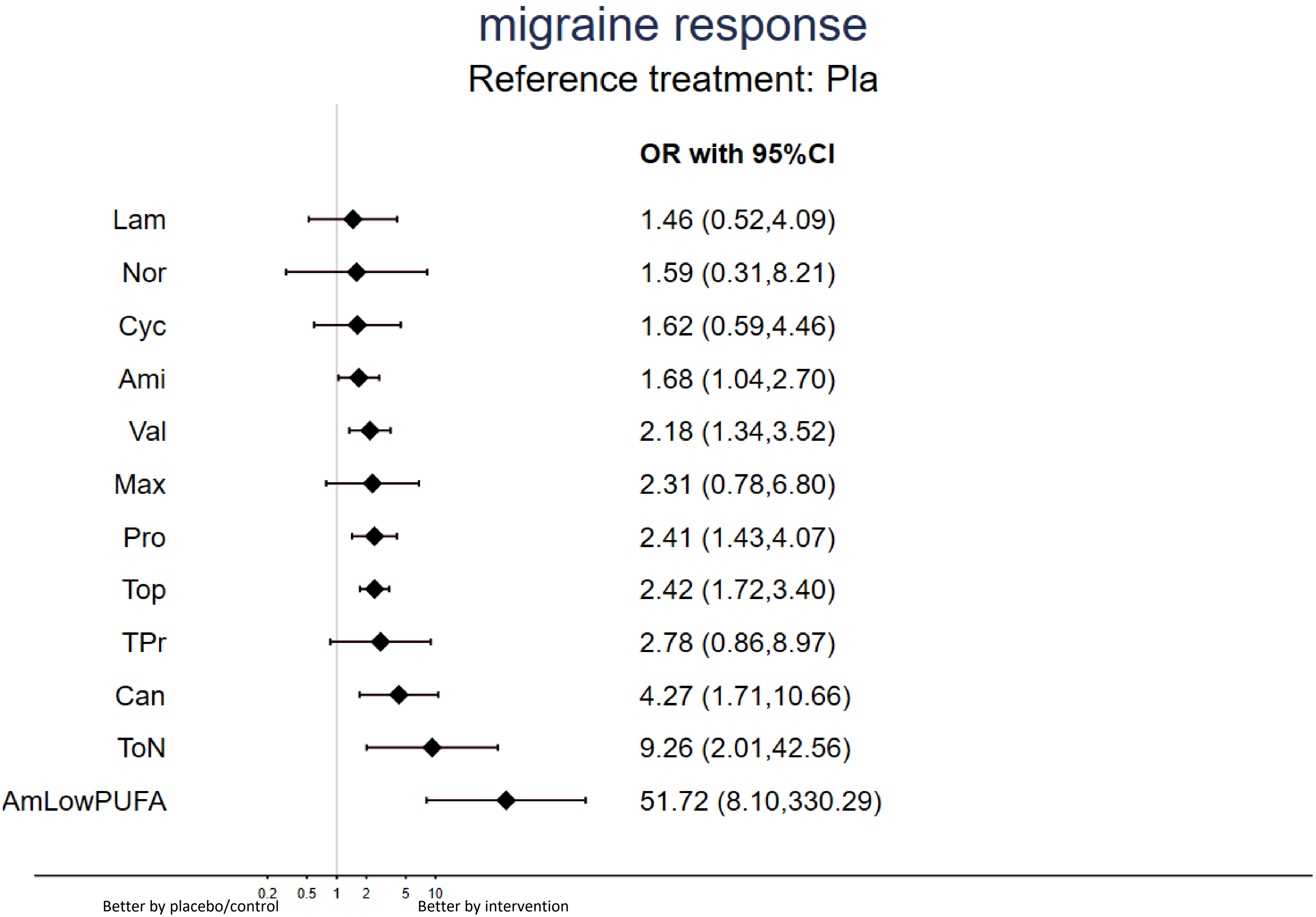

eFigure 2F forest plot of secondary outcome: response rate

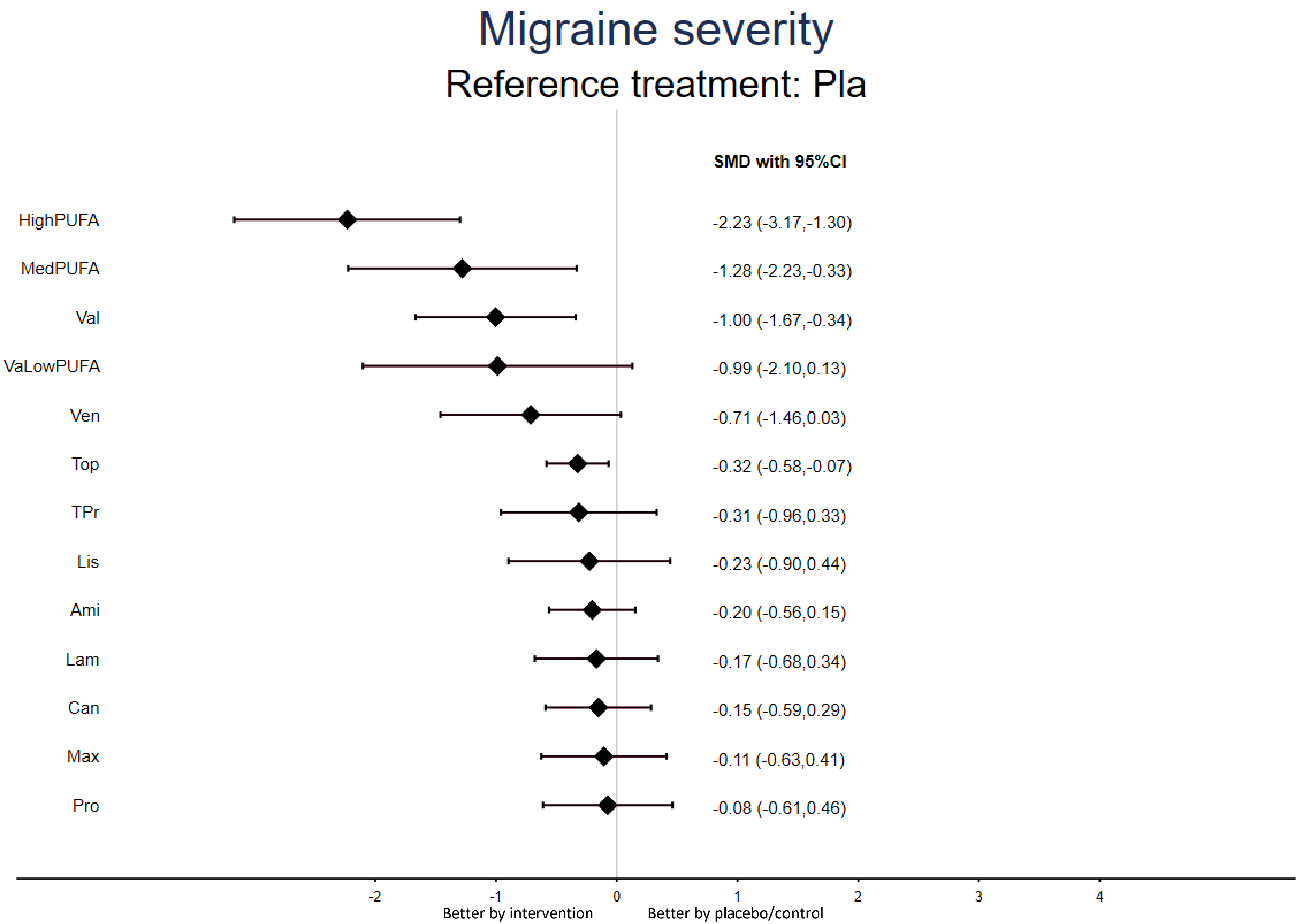

eFigure 2G forest plot of secondary outcome: migraine severity

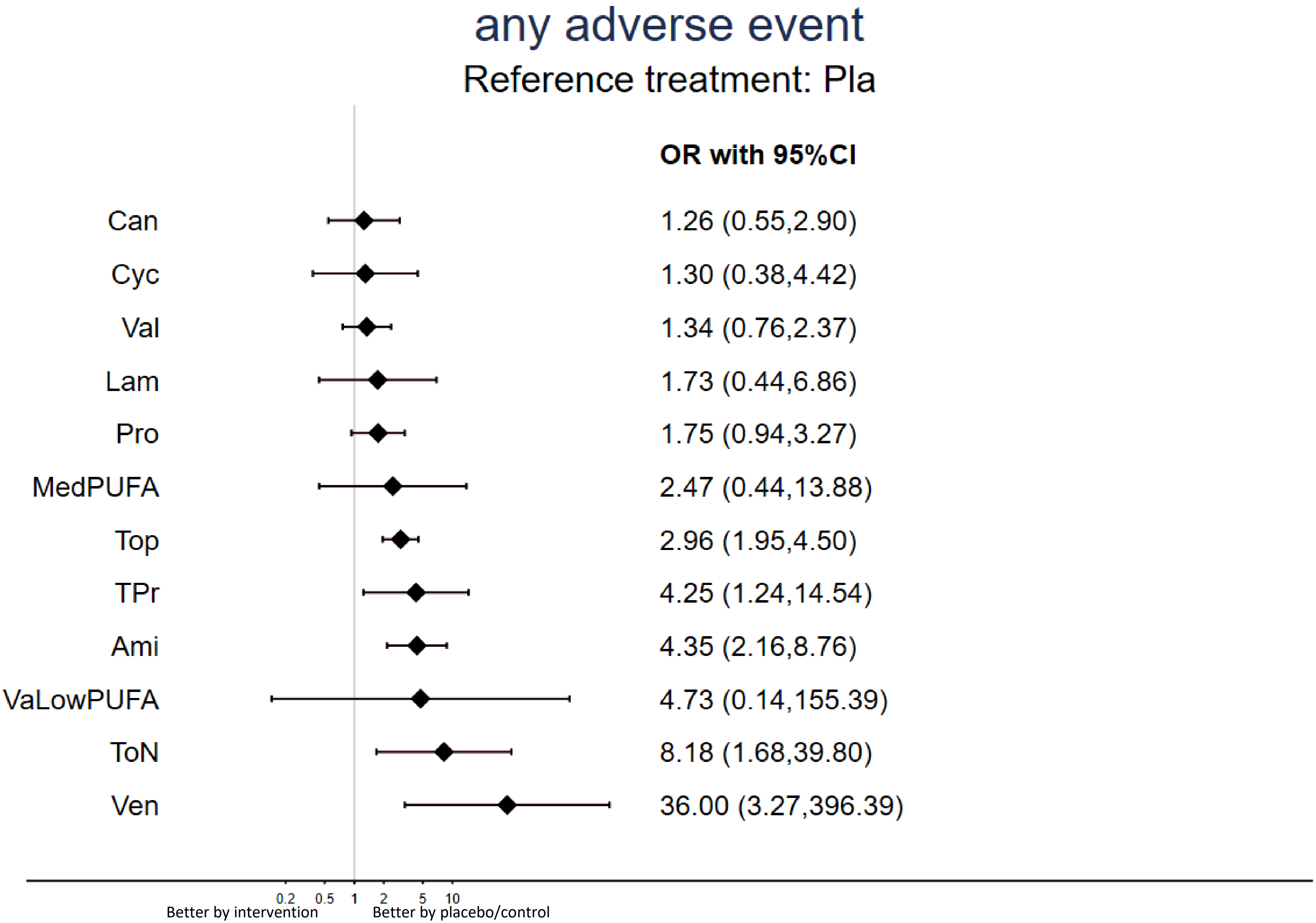

eFigure 2H forest plot of safety profile: rate of any adverse event

Figure legend of eFigure 2A-2H

Abbreviation: 95%CI: 95% confidence interval; Ami: amitriptyline; AmLowPUFA: low dosage n3PUFA + amitriptyline; AMSTAR: assessing the methodological quality of systematic review; Bot: Botox-A; Can: candesartan; CGRP: calcitonin gene-related peptide; Cyc: cyclandelate; DHA: docosahexaenoic acid; EPA: eicosapentaenoic acid; ES: effect size; HighPUFA: high dosage n3PUFA; Lam: lamotrigine; Lis: lisinopril; Max: Maxepa (omega-3 polyunsaturated fatty acids, EPA/DHA: 180mg/120mg x 6 pills); MedPUFA: medium dosage n3PUFA; Mem: memantine; NAM: network meta-analysis; Nor: nortriptyline; OR: odds ratio; Pla: Placebo; PRISMA: Preferred Reporting Items for Systematic Reviews and Meta-Analyses; Pro: propranolol; PUFA: polyunsaturated fatty acid; RCT: randomized controlled trial; SMD: standardized mean difference; SUCRA: surface under the cumulative ranking curve; ToN: topiramate + nortriptyline; Top: topiramate; TPr: topiramate + propranolol; TVGT: trigeminal nerve-trigeminocervical complex-ventroposteromedial thalamic nucleus; Val: valproate; VaLowPUFA: low dosage n3PUFA + valproate; Ven: venlafaxine

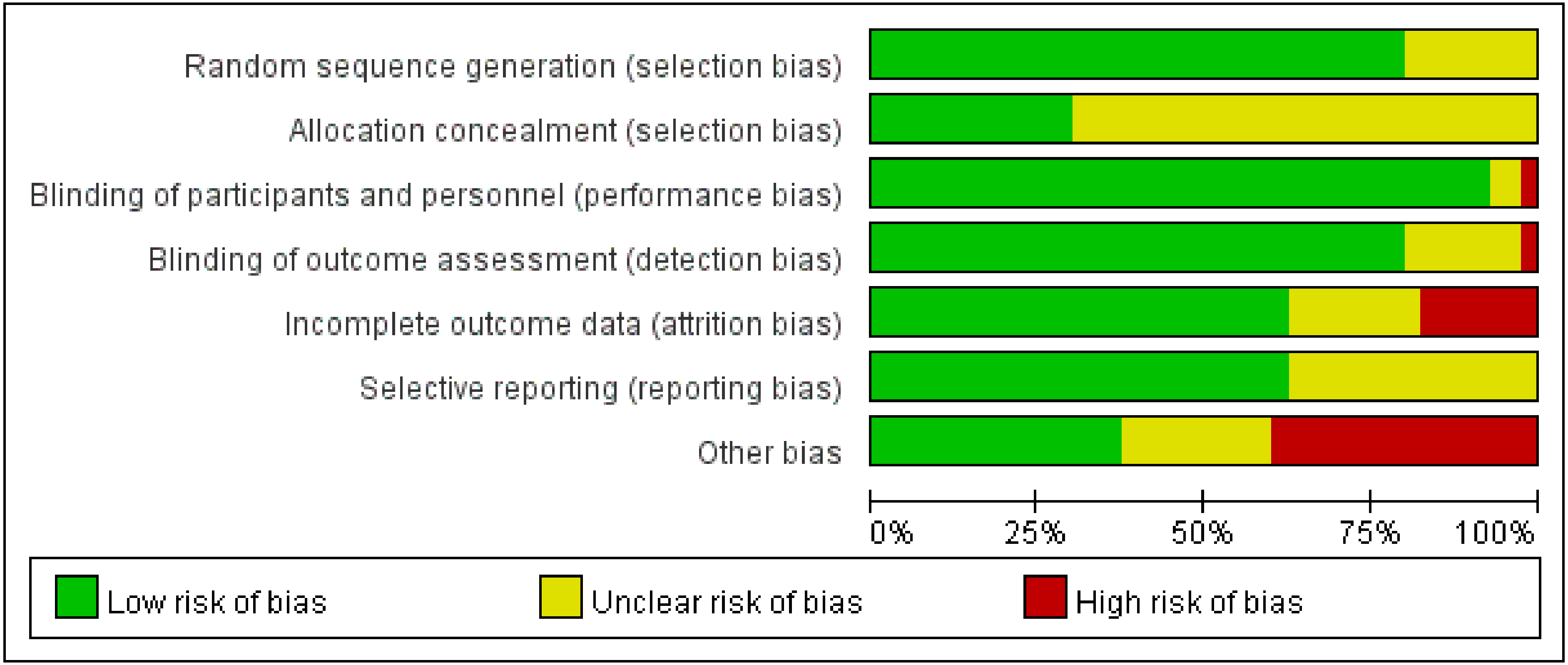

eFigure 3A overview of risk of bias

Study	Random sequence generation (selection bias)	Allocation concealment (selection bias)	Blinding of participants and personnel (performance bias)	Blinding of outcome assessment (detection bias)	Incomplete outcome data (attrition bias)	Selective reporting (reporting bias)	Other bias
Abdolahi, M. (2021)	+	?	+	+	+	+	?
Apostol, G. (2008)	+	?	+	+	?	+	−
Brandes, J.L. (2004)	+	+	+	+	+	+	−
Couch, J.R. (1979)	+	?	+	+	+	?	+
Couch, J.R. (2011)	+	?	+	+	?	+	+
Diener, H.C. (1996)	?	?	+	+	+	?	?
Diener, H.C. (2004)	?	?	+	?	?	+	−
Diener, H.C. (2007)	+	?	+	+	+	+	?
Dodick, D.W. (2009)	+	+	+	+	−	?	−
Ebrahimi-Monfared, M. (2017)	+	?	+	+	?	+	+
Edwards, K.R. (2003)	+	+	?	?	+	+	−
Fayyazi, A. (2016)	+	?	+	+	+	+	?
Freitag, F.G. (2002)	+	+	+	+	−	?	−
Goncalves, A.L. (2016)	+	?	+	+	?	+	+
Gupta, P. (2007)	+	+	+	+	+	?	+
Harel, Z. (2002)	+	?	+	+	+	?	+
Jensen, R. (1994)	+	+	?	?	+	+	−
Kaniecki, R.G. (1997)	+	?	−	−	−	+	−
Klapper, J. (1997)	?	?	+	+	+	?	−
Krymchantowski, A.V. (2012)	+	?	+	+	−	+	+
Lewis, D. (2009)	+	+	+	+	+	?	−
Lipton, R.B. (2011)	+	+	+	+	+	?	−
Mathew, N.T. (1995)	?	?	+	+	+	+	−
Mei, D. (2004)	+	?	+	?	−	+	+
Ozyalcin, S.N. (2005)	?	?	+	+	+	?	+
Powers, S.W. (2017)	+	?	+	+	+	+	?
Pradalier, A. (2001)	+	?	+	+	+	?	+
Schrader, H. (2001)	+	+	+	+	+	+	?
Silberstein, S.D. (2004)	+	+	+	+	?	+	−
Silberstein, S.D. (2006)	?	?	+	?	−	+	−
Silberstein, S.D. (2007)	+	?	+	+	?	+	+
Silberstein, S.D. (2012)	+	?	+	+	+	+	?
Silvestrini, M. (2003)	+	?	+	+	+	?	+
Soares, A.A. (2018)	?	?	+	+	+	+	?
Storey, J.R. (2001)	?	?	+	?	+	+	−
Stovner, L.J. (2014)	+	+	+	+	+	+	?
Tronvik, E. (2003)	+	?	+	+	+	?	+
Winner, P. (2005)	+	+	+	+	+	?	+
Winner, P. (2006)	+	?	+	?	−	?	−
Ziegler, D.K. (1987)	+	?	+	+	?	+	+

eFigure 3B detailed risk of bias in each study

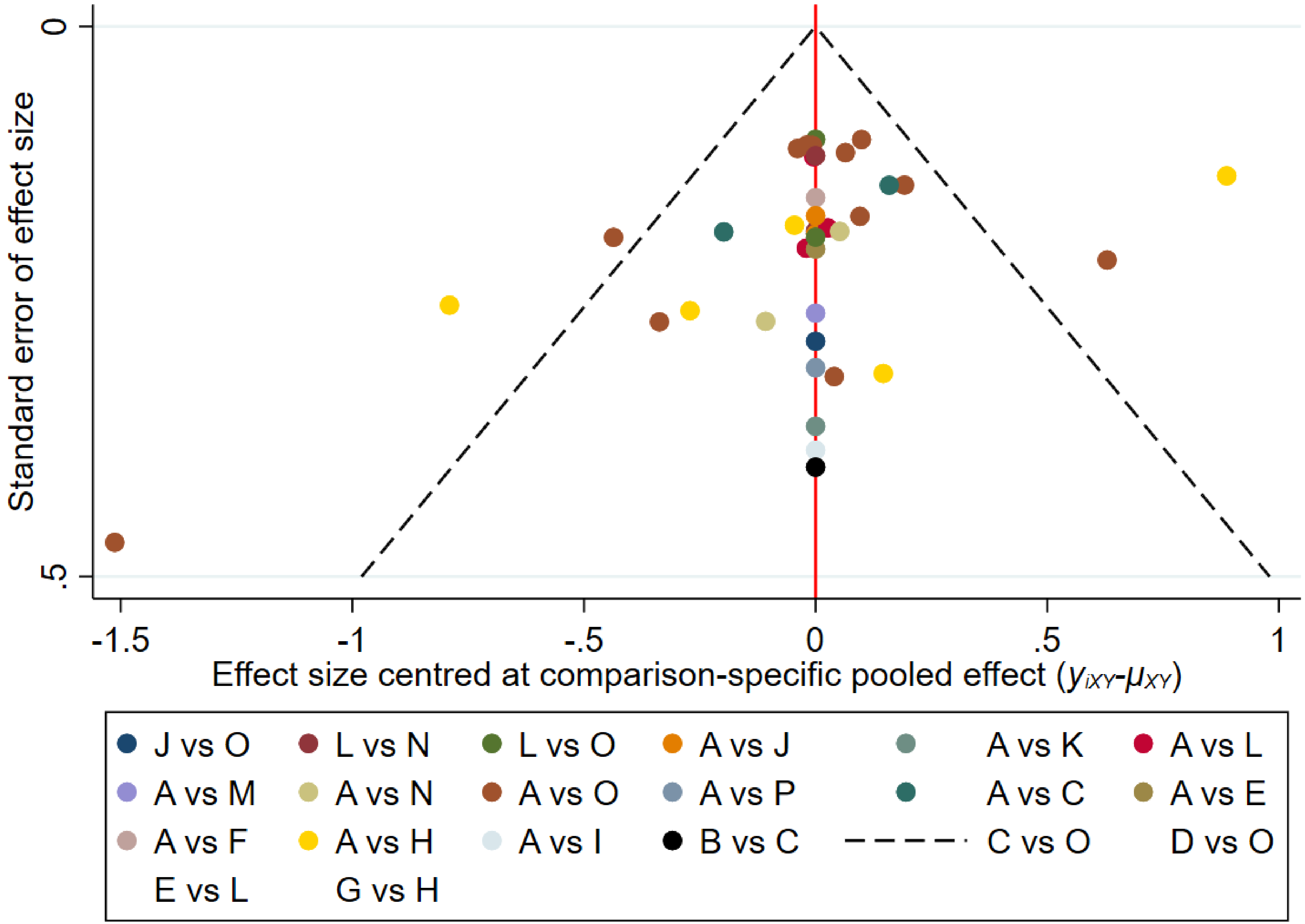

eFigure 4A Funnel plot of primary outcome: migraine frequency

Treatments used in eFigure 4A

A:	Pla
B:	AmLowPUFA
C:	Ami
D:	TPr
E:	Cyc
F:	Max
G:	VaLowPUFA
H:	Val
I:	MedPUFA
J:	Lam
K:	HighPUFA
L:	Pro
M:	Lis
N:	Can
O:	Top
P:	Ven

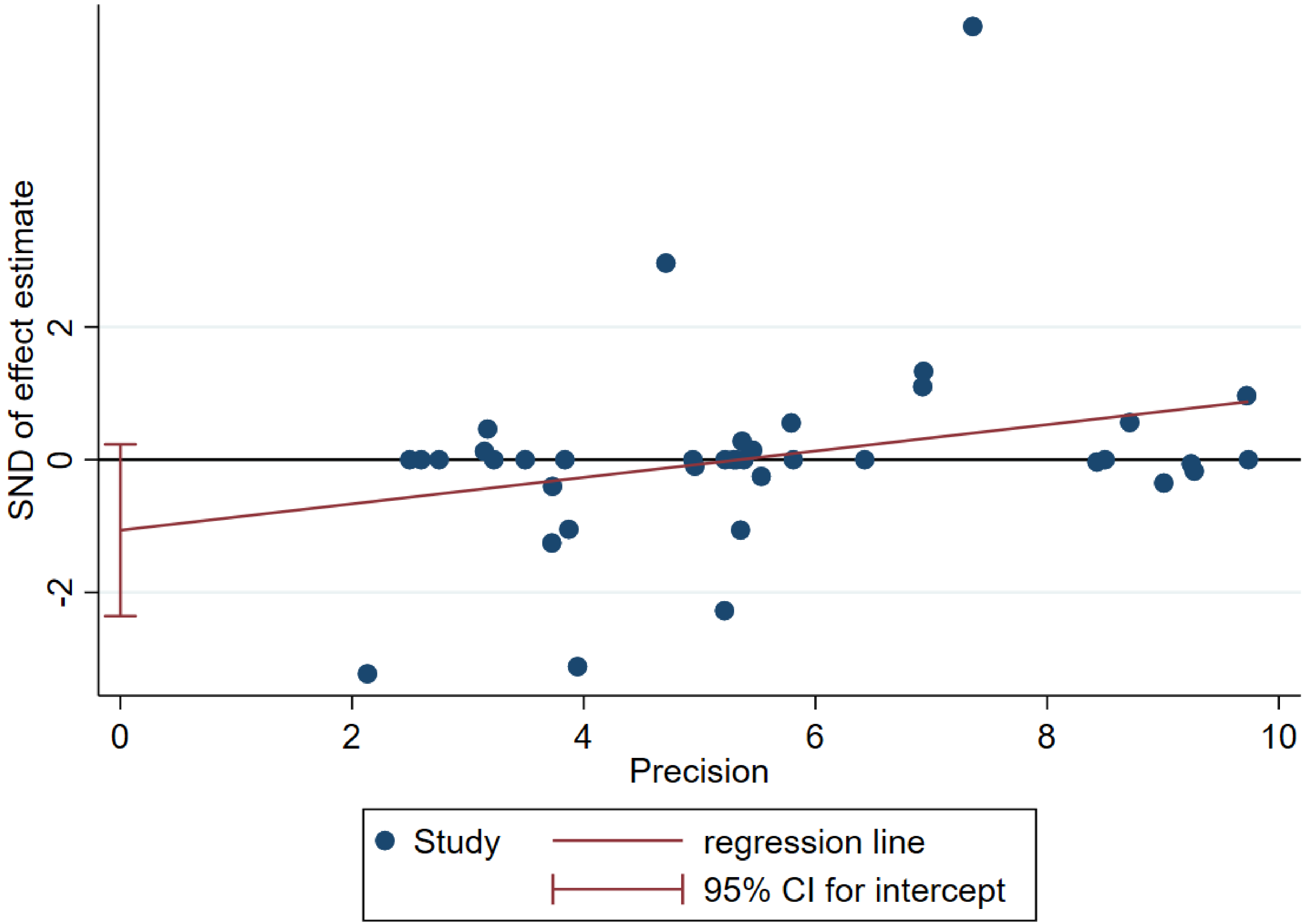

eFigure 4B Egger's regression of primary outcome: migraine frequency

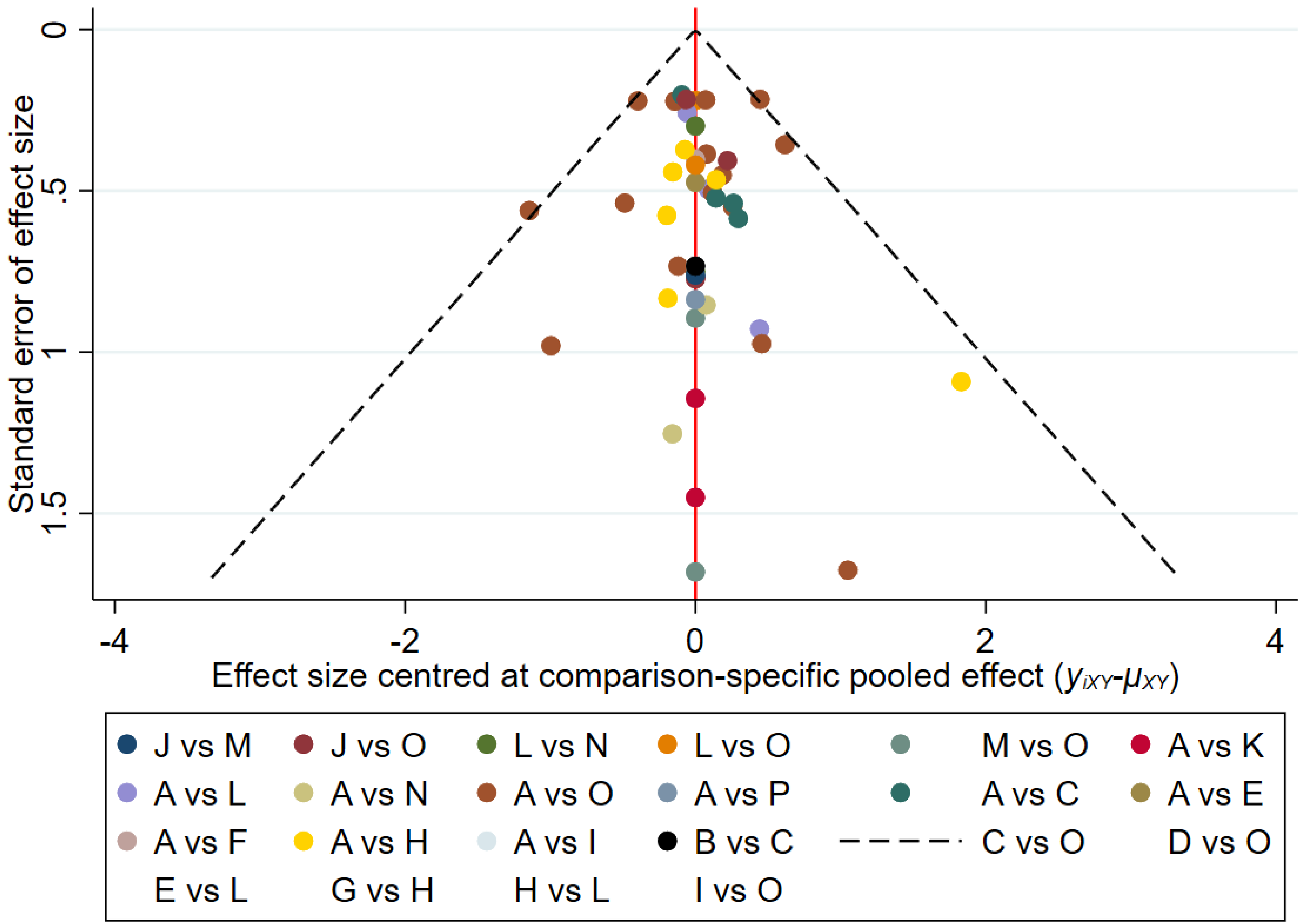

eFigure 4C Funnel plot of primary outcome: acceptability in aspect of drop out rate

Treatments used in eFigure 4C

A:	Pla
B:	AmLowPUFA
C:	Ami
D:	TPr
E:	Cyc
F:	Max
G:	VaLowPUFA
H:	Val
I:	Lam
J:	ToN
K:	HighPUFA
L:	Pro
M:	Nor
N:	Can
O:	Top
P:	Ven

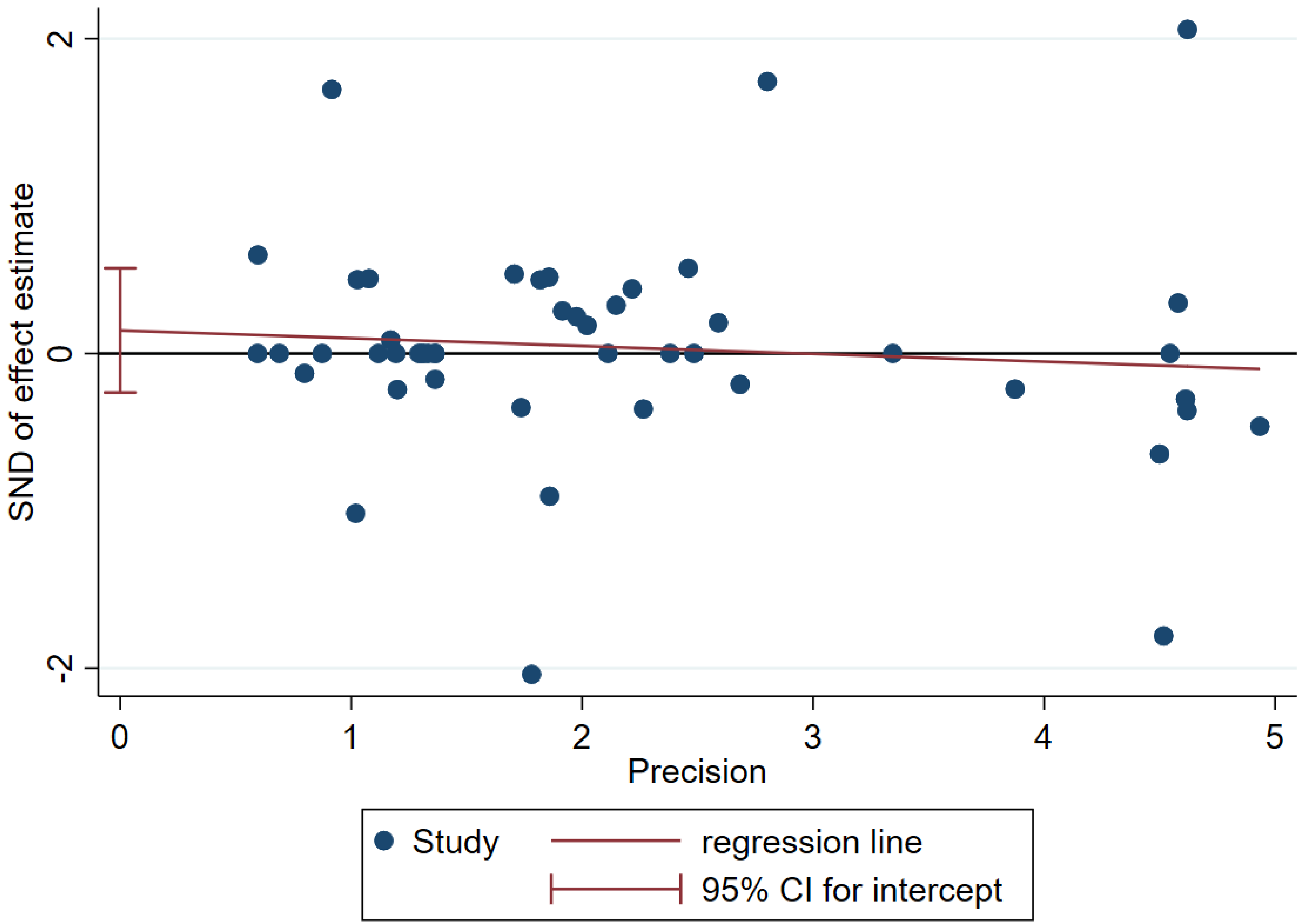

eFigure 4D Egger's regression of primary outcome: acceptability in aspect of drop out rate

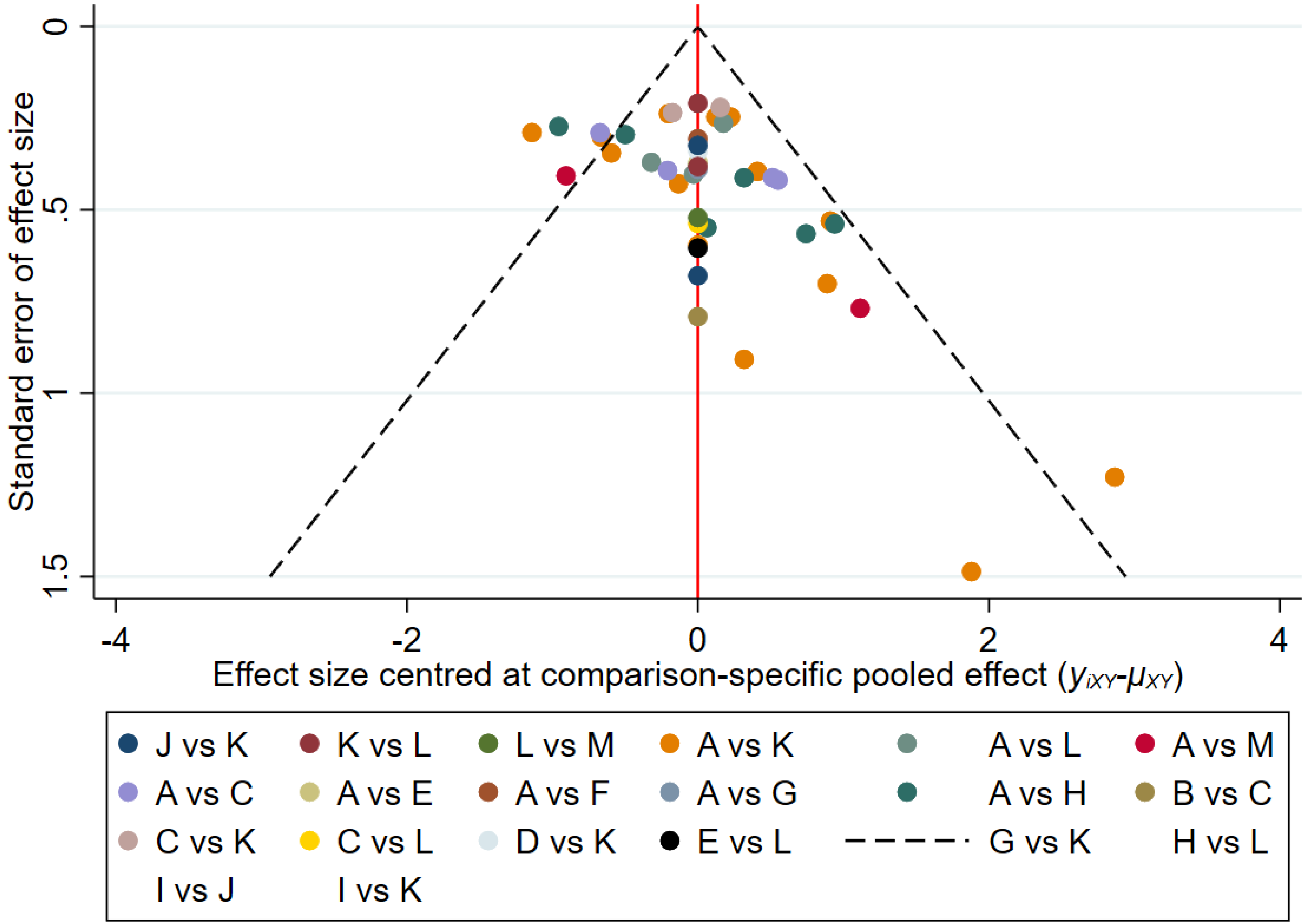

eFigure 4E Funnel plot of secondary outcome: response

Treatments used in eFigure 4E

A:	Pla
B:	AmLowPUFA
C:	Ami
D:	TPr
E:	Cyc
F:	Max
G:	Lam
H:	Val
I:	ToN
J:	Nor
K:	Top
L:	Pro
M:	Can

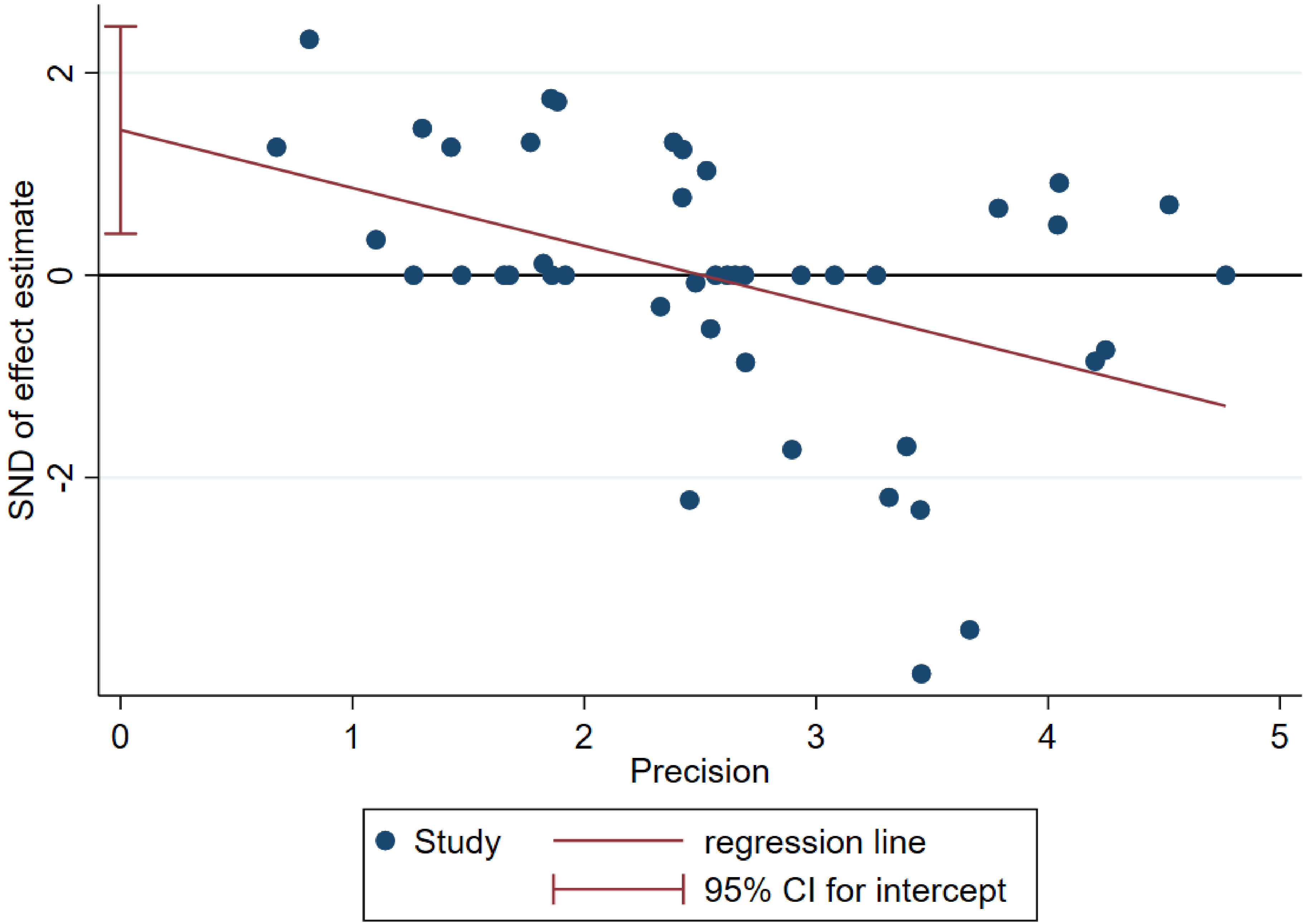

eFigure 4F Egger's regression of secondary outcome: response

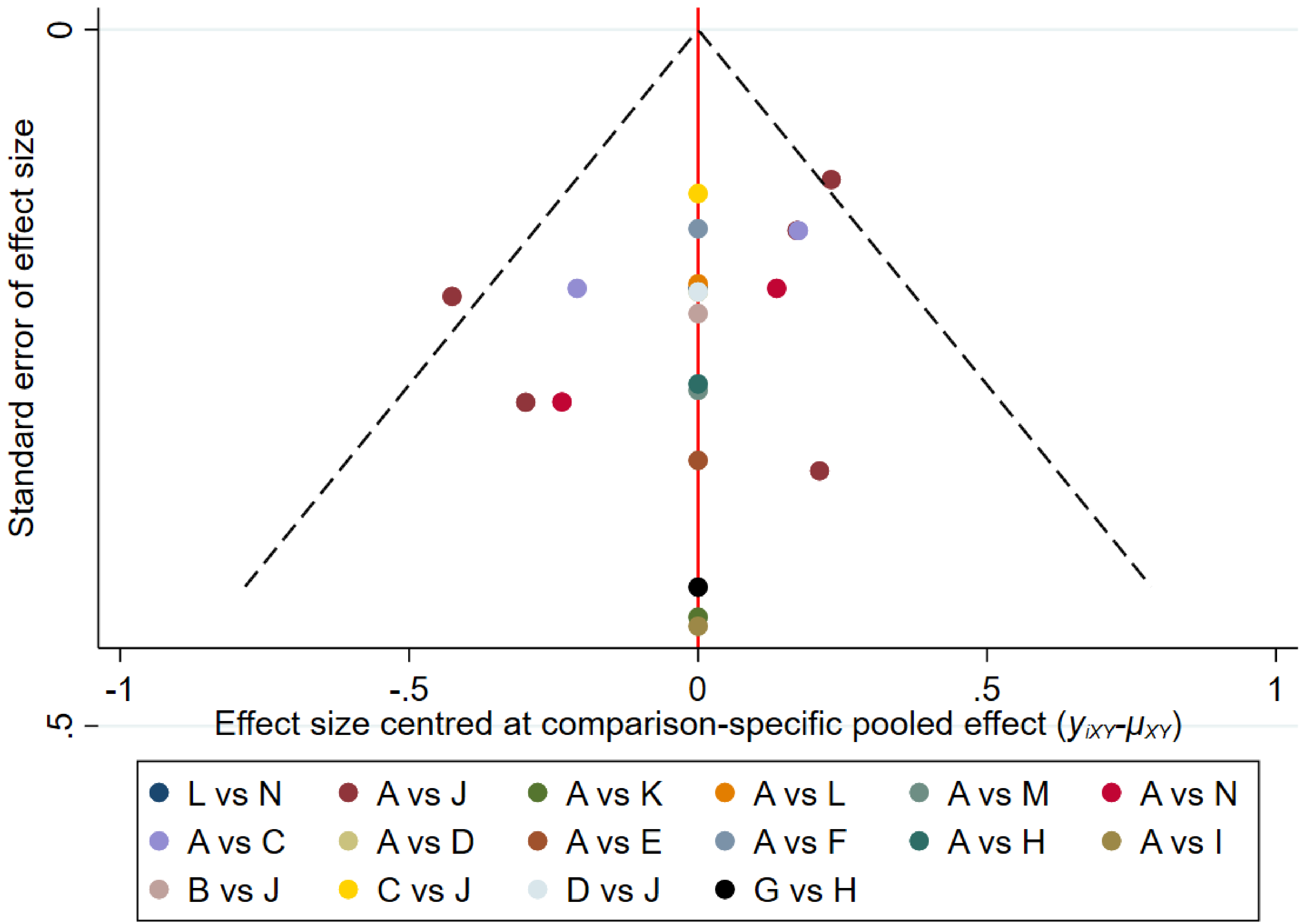

eFigure 4G Funnel plot of secondary outcome: migraine severity

Treatments used in eFigure 4G

A:	Pla
B:	TPr
C:	Ami
D:	Lam
E:	Ven
F:	Max
G:	VaLowPUFA
H:	Val
I:	MedPUFA
J:	Top
K:	HighPUFA
L:	Pro
M:	Lis
N:	Can

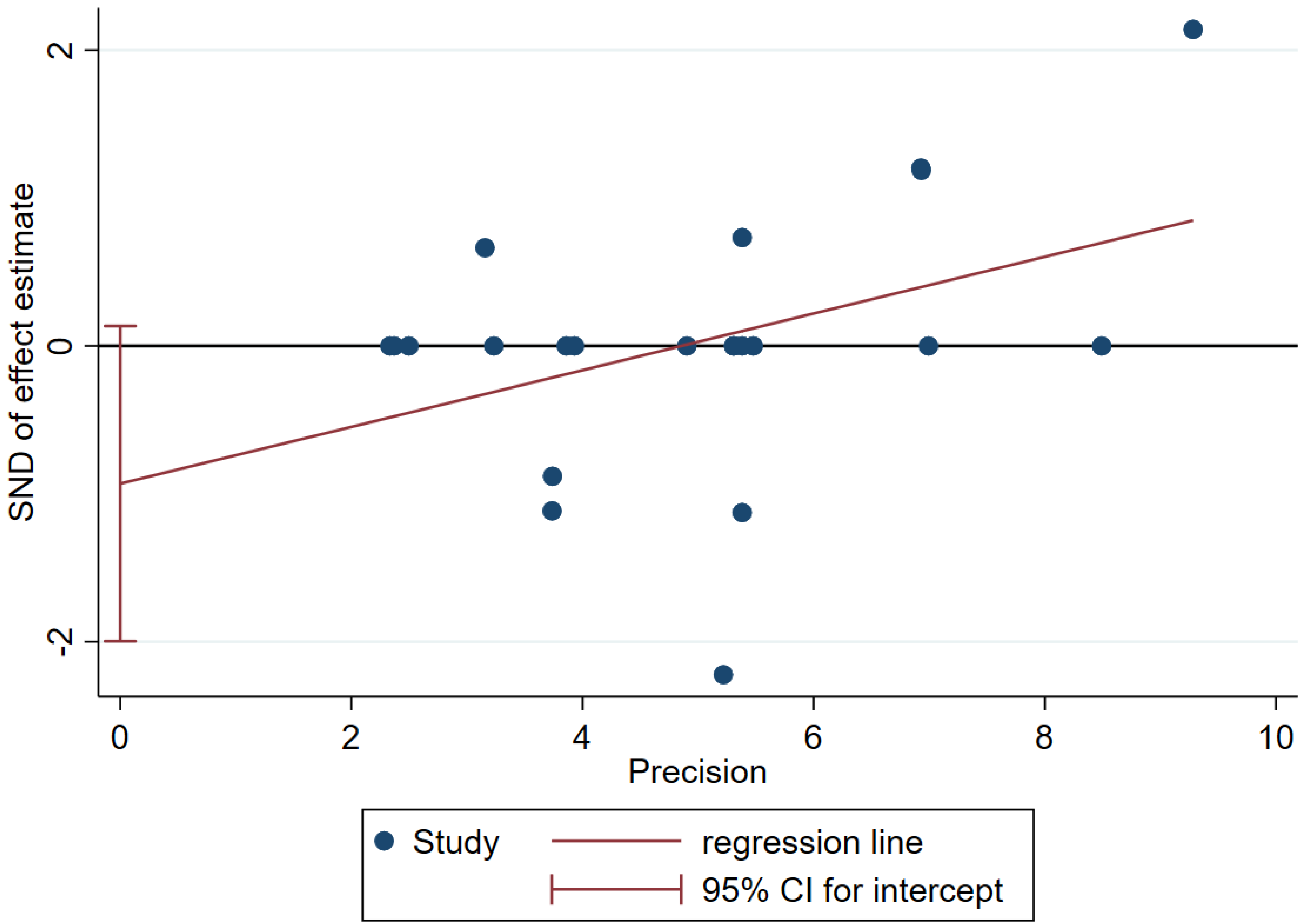

eFigure 4H Egger's regression of secondary outcome: migraine severity

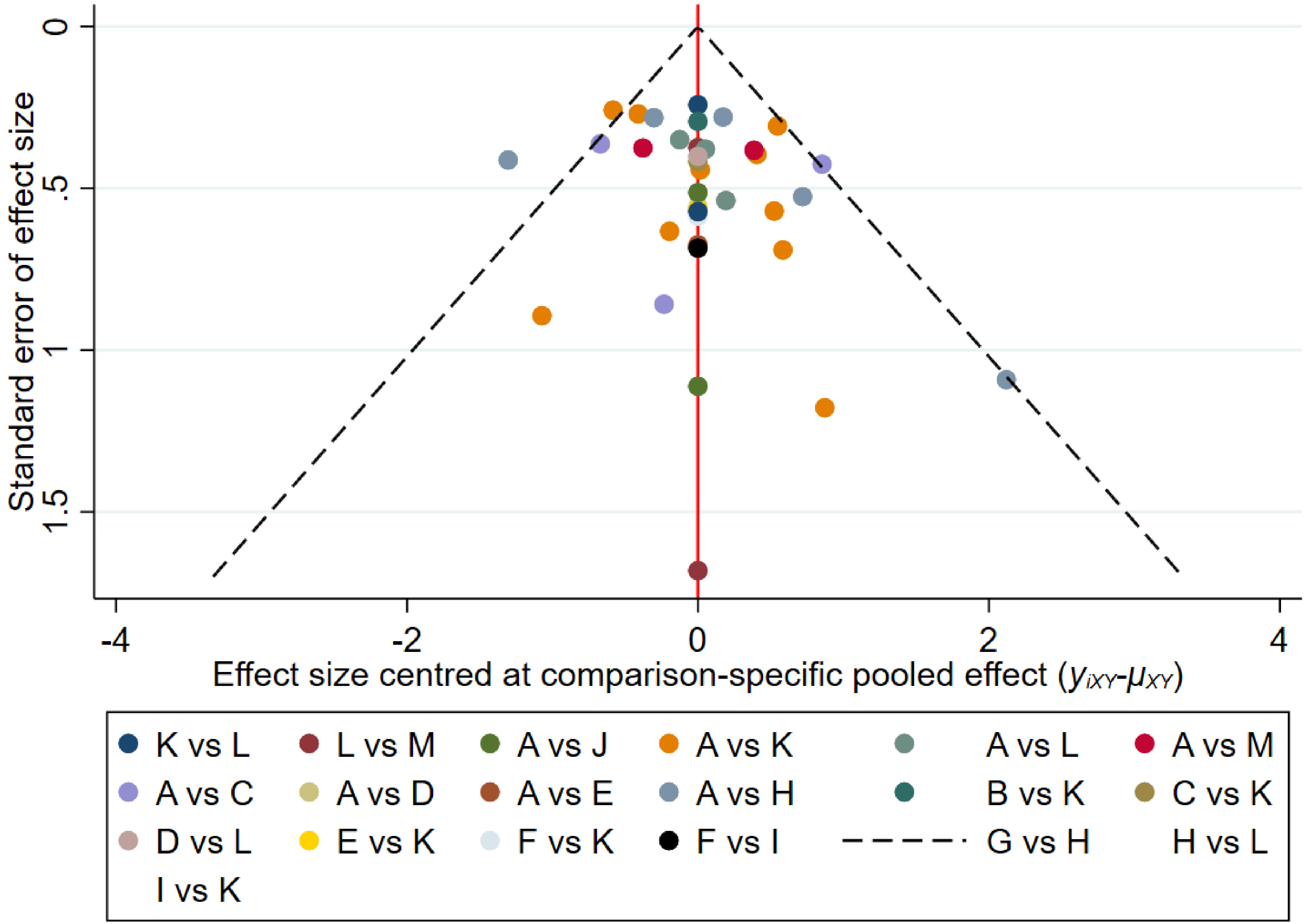

eFigure 4I Funnel plot of safety profile: rate of any adverse event

Treatments used in eFigure 4I

A:	Pla
B:	TPr
C:	Ami
D:	Cyc
E:	Lam
F:	ToN
G:	VaLowPUFA
H:	Val
I:	MedPUFA
J:	Ven
K:	Top
L:	Pro
M:	Can

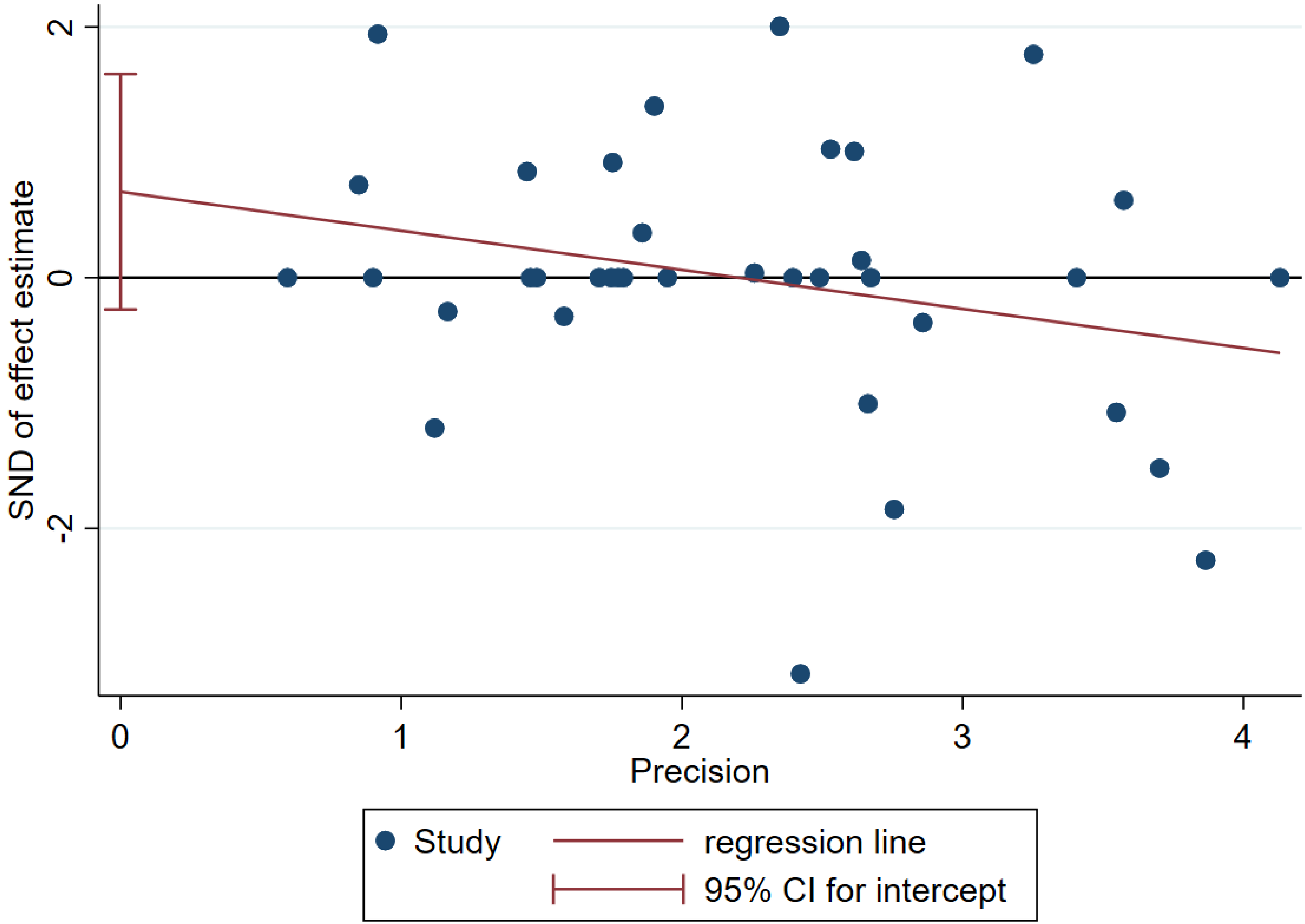

eFigure 4J Egger's regression of safety profile: rate of any adverse event

Figure legend of eFigure 4A-4J

Abbreviation: 95%CI: 95% confidence interval; Ami: amitriptyline; AmLowPUFA: low dosage n3PUFA + amitriptyline; AMSTAR: assessing the methodological quality of systematic review; Bot: Botox-A; Can: candesartan; CGRP: calcitonin gene-related peptide; Cyc: cyclandelate; DHA: docosahexaenoic acid; EPA: eicosapentaenoic acid; ES: effect size; HighPUFA: high dosage n3PUFA; Lam: lamotrigine; Lis: lisinopril; Max: Maxepa (omega-3 polyunsaturated fatty acids, EPA/DHA: 180mg/120mg x 6 pills); MedPUFA: medium dosage n3PUFA; Mem: memantine; NAM: network meta-analysis; Nor: nortriptyline; OR: odds ratio; Pla: Placebo; PRISMA: Preferred Reporting Items for Systematic Reviews and Meta-Analyses; Pro: propranolol; PUFA: polyunsaturated fatty acid; RCT: randomized controlled trial; SMD: standardized mean difference; SUCRA: surface under the cumulative ranking curve; ToN: topiramate + nortriptyline; Top: topiramate; TPr: topiramate + propranolol; TVGT: trigeminal nerve-trigeminocervical complex-ventroposteromedial thalamic nucleus; Val: valproate; VaLowPUFA: low dosage n3PUFA + valproate; Ven: venlafaxine
